# Severe kidney dysfunction in sialidosis mice reveals an essential role for neuraminidase 1 in reabsorption

**DOI:** 10.1172/jci.insight.166470

**Published:** 2023-10-23

**Authors:** Ikhui Kho, Ekaterina P. Demina, Xuefang Pan, Irene Londono, Christopher W. Cairo, Luisa Sturiale, Angelo Palmigiano, Angela Messina, Domenico Garozzo, Roth-Visal Ung, Fabrice Mac-Way, Éric Bonneil, Pierre Thibault, Mathieu Lemaire, Carlos R. Morales, Alexey V. Pshezhetsky

**Affiliations:** 1 CHU Sainte-Justine Research Center, University of Montreal, Montreal, Québec, Canada.; 2 Department of Anatomy and Cell Biology, McGill University, Montreal, Québec, Canada.; 3 Department of Chemistry, University of Alberta, Edmonton, Alberta, Canada.; 4 CNR, Institute for Polymers, Composites and Biomaterials, Catania, Italy.; 5 CHU de Québec Research Center, L’Hôtel-Dieu de Québec Hospital, Faculty and Department of Medicine, University Laval, Québec City, Québec, Canada.; 6 Institute for Research in Immunology and Cancer, University of Montreal, Montreal, Québec, Canada.; 7 Division of Nephrology, The Hospital for Sick Kids, Faculty of Medicine, University of Toronto, Ontario, Canada.; 8 Cell Biology Program, SickKids Research Institute, Toronto, Ontario, Canada.

**Keywords:** Genetics, Nephrology, Chronic kidney disease, Glycobiology, Lysosomes

## Abstract

Sialidosis is an ultra-rare multisystemic lysosomal disease caused by mutations in the neuraminidase 1 (*NEU1*) gene. The severe type II form of the disease manifests with a prenatal/infantile or juvenile onset, bone abnormalities, severe neuropathology, and visceromegaly. A subset of these patients present with nephrosialidosis, characterized by abrupt onset of fulminant glomerular nephropathy. We studied the pathophysiological mechanism of the disease in 2 NEU1-deficient mouse models, a constitutive *Neu1*-knockout, *Neu1*^ΔEx3^, and a conditional phagocyte-specific knockout, *Neu1*^Cx3cr1*Δ*Ex3^. Mice of both strains exhibited terminal urinary retention and severe kidney damage with elevated urinary albumin levels, loss of nephrons, renal fibrosis, presence of storage vacuoles, and dysmorphic mitochondria in the intraglomerular and tubular cells. Glycoprotein sialylation in glomeruli, proximal distal tubules, and distal tubules was drastically increased, including that of an endocytic reabsorption receptor megalin. The pool of megalin bearing O-linked glycans with terminal galactose residues, essential for protein targeting and activity, was reduced to below detection levels. Megalin levels were severely reduced, and the protein was directed to lysosomes instead of the apical membrane. Together, our results demonstrated that desialylation by NEU1 plays a crucial role in processing and cellular trafficking of megalin and that NEU1 deficiency in sialidosis impairs megalin-mediated protein reabsorption.

## Introduction

Sialic acids are found attached to the terminal ends of glycoproteins and glycolipids on the surface of all mammalian cells, forming a glycocalyx that functions as a barrier and mediates cell-cell interaction (1, 2). In kidneys, sialic acids are remarkably enriched at the membranes of the glomerular endothelial cells and at the basement membrane, supporting the glomerular filtration barrier and maintaining cellular structural integrity (3, 4). Aberrant glomerular sialylation caused by genetic or environmental factors is linked to multiple kidney diseases. For example, glomerular hyposialylation is observed in severe pneumococcal infections (5), as well as in about 50% of patients with a sialic acid transporter defect (6).

A similar pathology is described in multiple animal models of kidney disease, including mice exposed to pneumococcal neuraminidase (NanA) (7), puromycin amino nucleoside (8), or polylysine (9) or mice with genetic defects that disrupt sialic acid metabolism: e.g., *Cmas* (10), *C1galt1* (11), *ST3GAL1* (12, 13), or acetylglucosamine 2-epimerase/N-acetylmannosamine kinase (*Gne*) (14). Specifically, glomerular hyposialylation in the animal models of kidney damage, induced by puromycin amino nucleoside, results in effacement of podocyte foot processes and proteinuria (4, 8). Glomerulopathy is also observed in mouse models of the genetic deficiency in GNE, the key enzyme of sialic acid synthesis (15). Treatment of *Gne*-deficient mice with the sialic acid precursor, N-acetylmannosamine, ameliorates hyposialylation, reduces albuminuria, and partially restores glomerular architecture (15). Significant differences in sialylation of kidney proteins were also observed in several rodent models of chronic kidney disease (16).

On the other hand, kidney pathology is also associated with a deficiency of neuraminidase 1 (NEU1), which cleaves terminal sialic acids from glycan chains in glycoproteins. A genome-wide association study based on samples from over 1 million individuals revealed a significant association (*P* < 10^–14^) between a G > A variant in the 3′ untranslated region of the *NEU1* gene and decreased glomerular filtration rate (17), later replicated (*P* < 10^–36^) by an independent study (18). The G > A variant is present in all populations sampled in the Genome Aggregation Database with allele frequencies ranging from 3% to 16%; however, its functional impact is unclear.

Pathogenic genetic *NEU1* variants cause sialidosis (Online Mendelian Inheritance in Man [OMIM] #256550), a rare autosomal recessive disease with a prevalence of less than 1/1,000,000 live births. NEU1 deficiency blocks catabolism of sialylated glycoproteins and oligosaccharides and results in progressive lysosomal accumulation and urinary excretion of sialylated oligosaccharides and glycopeptides, eventually leading to multisystem dysfunction (19, 20).

Sialidosis is divided into 2 subtypes with different onset age and severity. Type I manifests as a relatively mild, late-onset disease with patients suffering from myoclonus, progressive vision failure, and mild cognitive impairment (20). Type II is an early-onset, infantile/juvenile form, in which patients display abnormal somatic features, hepatosplenomegaly, and substantially impaired intellectual and adaptive functioning (21). A subset of type II patients, presenting with a severe nephrotic syndrome, are described as having nephrosialidosis (21, 22). Previous work identified severe pathological alterations in the glomeruli of patients with nephrosialidosis, including diffused fusion of podocyte foot processes and vacuolization of podocytes (23). These vacuoles accumulate glycoconjugates containing terminal sialic acid, α-linked mannose, and *N*-acetylgalactosamine residues (24, 25). These glycoconjugates are also released by podocytes in the urine ultrafiltrate, resulting in a drastically increased concentration of sialylated oligosaccharides in the urine of patients with sialidosis (20, 23) and *Neu1*-null mice (26).

Notably, the glomerular lesions alone do not fully explain the pathophysiology of nephrosialidosis. When compared with controls, the urine of patients is enriched not only with albumin (a marker of glomerular dysfunction) but also with low–molecular weight proteins (LMWPs) and soluble metabolites (21, 22) that are freely filtered at the glomeruli and reabsorbed at the proximal tubule. Previous work has established that in addition to its presence on lysosomes, NEU1 is present on the cell surface, where it desialylates multiple protein targets, and that NEU1 deficiency results in dramatic hypersialylation of membrane proteins (27, 28). Integrating all these findings, we hypothesized that the pathological kidney phenotype observed in mice and humans with NEU1 deficiency is complex and implicates hypersialylation-dependent defects in both glomeruli and proximal tubules.

In the current work, we demonstrate that NEU1 deficiency in constitutive knockout (KO) *Neu1*^ΔEx3^ and novel conditional phagocyte-specific *Neu1*^Cx3cr1*Δ*Ex3^ KO mice results in hypersialylation of endocytic receptor megalin, which disrupts its normal targeting to the tubular apical membranes and impairs protein reabsorption processes in the proximal tubules.

## Results

### Systemic pathology in Neu1^Cx3cr1ΔEx3^ and Neu1^ΔEx3^ strains.

Recent studies revealed that disease-associated microglia phagocytic cells play an essential role in the pathology of lysosomal storage diseases, including sialidosis (reviewed in ref. 29). To assess the contribution of NEU1-deficient phagocytic cells to systemic pathology in sialidosis, we have generated a phagocyte-specific conditional *Neu1*-KO (*Neu1*^Cx3cr1*Δ*Ex3^) and compared it with a previously described constitutive *Neu1*-KO mouse (*Neu1*^ΔEx3^) (30). *Neu1*^Cx3cr1*Δ*Ex3^ mice were generated by producing a *Neu1^loxPEx3^* strain with the *Neu1* exon 3, flanked with the *loxP* sites, and crossing it with the mouse, expressing the Cre recombinase under the control of the chemokine CX3C motif receptor 1 (*Cx3cr1*) gene promoter (Supplemental Figure 1; supplemental material available online with this article; https://doi.org/10.1172/jci.insight.166470DS1). This resulted in the deletion of exon 3 in all *Cx3cr1*-expressing cells (Supplemental Figure 1).

Homozygous *Neu1*^Cx3cr1*Δ*Ex3^ mice showed normal appearance and general behavior indistinguishable from that of WT or heterozygous animals until the age of 20 weeks. Mice were fertile and produced normal litter sizes. Neurological assessment (gait, posture, avoidance response, righting reflex, inverted wire screen test) conducted on the group of 10 *Neu1*^Cx3cr1*Δ*Ex3^ and WT mice at the age of 8 weeks did not reveal signs of overt neuromuscular pathology.

Heterozygous *Neu1*^ΔEx3^ breeding pairs also produced average litters (3–8 pups), and homozygous *Neu1*^ΔEx3^ mice were born at an expected Mendelian frequency of about 20%. They, however, showed slower growth and smaller size than their heterozygous or WT littermates, resembling the previously described phenotype (26). Male *Neu1*^ΔEx3^ mice were sterile, while female *Neu1*^ΔEx3^, crossed with heterozygous males, could infrequently produce a small-size (1–2 pups) first litter but never a second litter.

**Figure 1:**
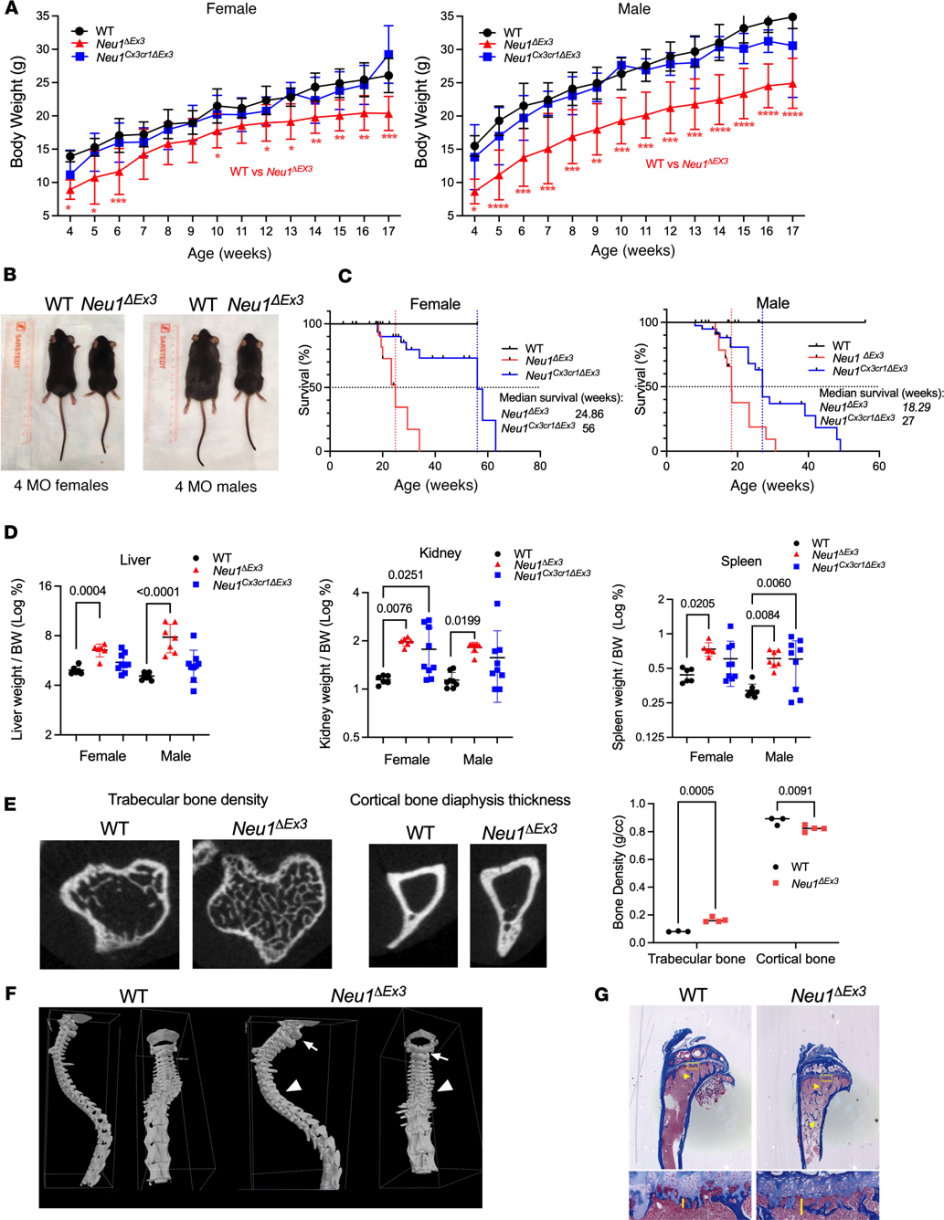
Pathophysiological phenotypes of *Neu1*^ΔEx3^ and *Neu1*^Cx3cr1*Δ*Ex3^ mice. (**A**) Male and female *Neu1^ΔEx3^* mice have a significantly reduced body mass compared with WT mice of the same age. Body mass was measured weekly, from 4 to 17 weeks of age. *P* values were calculated using 2-way ANOVA with a Bonferroni post hoc test. *P < 0.05, **P < 0.01, ***P < 0.001, ****P < 0.0001. (**B**) Representative images of 4-month-old *Neu1^ΔEx3^* mice and their sex-matched WT littermates. (**C**) Kaplan-Meier plots showing the survival of *Neu1^ΔEx3^* and *Neu1^Cx3cr1ΔEx3^* mice and their WT counterparts. (**D**) *Neu1^ΔEx3^* mice present with visceromegaly of the kidney, liver, and spleen in both males and females. *Neu1^Cx3cr1ΔEx3^* mice present a similar trend with significant differences from WT littermates, observed for spleens of males and kidneys of females. *P* values were calculated using 1-way ANOVA with a Dunnett post hoc test. (**E**–**G**) Bone abnormalities in 4-month-old *Neu1^ΔEx3^* mice. (**E**) Micro-CT scan of tibia showed increased mineral density of the trabecular bone and reduced mineral content and density in the cortical diaphysis bone in *Neu1^ΔEx3^* compared with the WT mice. *P* values were calculated with 2-tailed *t* test. (**F**) A reconstructed 3D image of the spine showed thick and flattened spinous process (white arrow) of the cervical vertebra and short transverse process of the thoracic vertebra (white arrowhead). (**G**) Histology analysis reveals increased primary spongiosa (squares and vertical lines) and trabecular bone (arrows) in the *Neu1^ΔEx3^* mice.

The weight of male *Neu1*^ΔEx3^ mice was reduced by about 30%, and female *Neu1*^ΔEx3^ mice by about 20%, as compared with age- and sex-matched WT mice (Figure 1A). At the age of 4 months, *Neu1*^ΔEx3^ mice were visibly smaller than their heterozygous and WT littermates (Figure 1B). In contrast, *Neu1*^Cx3cr1*Δ*Ex3^ mice of both sexes showed no significant difference in weight (Figure 1A) or size from their WT littermates.

Starting from 4 weeks, both male and female *Neu1*^ΔEx3^ mice appeared physically unwell, displaying a hunched posture, mobility issues, and slower response to touch. Abnormal motor movements and clumsy gait were also noted. *Neu1*^ΔEx3^ mice also showed hydrocephalus (5% of mice), dental malocclusion, and a tendency to develop rectal prolapse seen in approximately 55% of mice (Supplemental Figure 2). With age, homozygous mice of both strains developed a severely distended bladder filled with urine (Supplemental Figure 2) and had to be euthanized. Other causes for euthanasia included asthenia, severe weight loss, and hydrocephalus. *Neu1*^ΔEx3^ male mice had the shortest median life span (18.3 weeks), followed by female *Neu1*^ΔEx3^ mice (24.9 weeks), male *Neu1*^Cx3cr1*Δ*Ex3^ mice (27 weeks), and female *Neu1*^Cx3cr1*Δ*Ex3^ mice (56 weeks) (Figure 1C). Pathological examination revealed enlargement of liver, kidneys, and spleen and reduced intra-abdominal fat in *Neu1*^ΔEx3^ compared with WT mice (Figure 1D and Supplemental Figure 2). *Neu1*^Cx3cr1*Δ*Ex3^ mice showed a similar trend; however, due to a large variability, a significant weight increase was observed only for kidneys of female *Neu1*^Cx3cr1*Δ*Ex3x^ and spleens of male *Neu1*^Cx3cr1*Δ*Ex3^ mice (Figure 1D).

Since bone abnormalities represent a frequent clinical feature in patients with sialidosis, we conducted a micro-CT scan of the *Neu1*^ΔEx3^ mice’s tibias, which detected increased mineral density of the trabecular bone whereas mineral content and density in the cortical diaphysis bone were reduced (Figure 1E). A reconstructed 3D image of the spine also showed thick and flattened spinous process of the cervical vertebra and short transverse process of the thoracic vertebra (Figure 1F). Despite being smaller in size and weight, the KO mice showed increased primary and secondary spongiosa bone volume, whereas the growth plate thickness seemed not to differ from those in WT (Figure 1G and Supplemental Table 1).

### Neu1^ΔEx3^ and Neu1^Cx3cr1ΔEx3^ mice show deficiency of NEU1 activity and increased lysosomal biogenesis in tissues.

**Figure 2:**
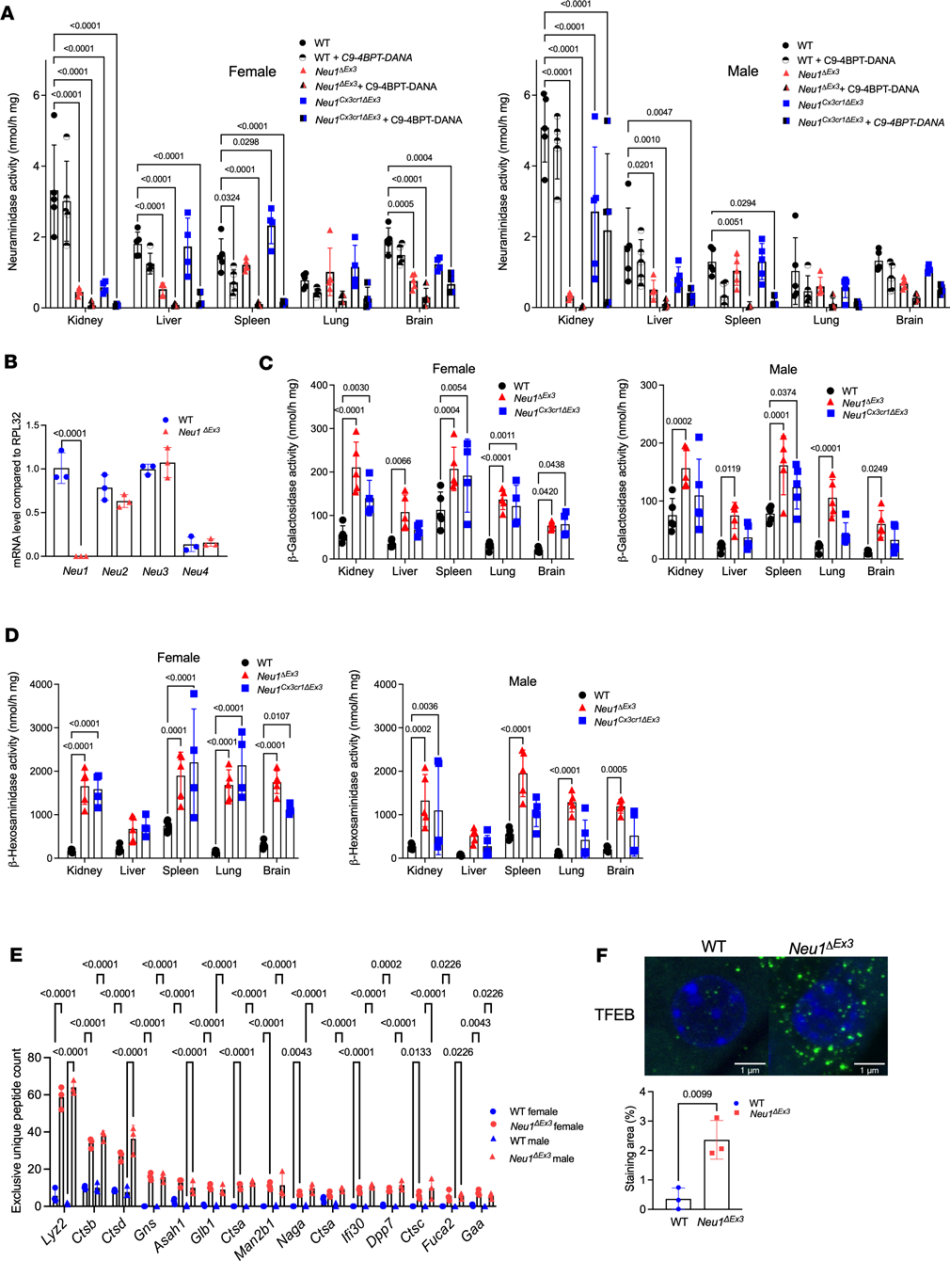
*Neu1*^ΔEx3^ and *Neu1*^Cx3cr1*Δ*Ex3^ homozygous mice show deficiency of NEU1 activity and increased lysosomal biogenesis in kidney tissues. (**A**) Total neuraminidase and NEU1 enzyme activity were measured in the tissue homogenates of 4-month-old WT, *Neu1^ΔEx3^*, and *Neu1^Cx3cr1ΔEx3^* homozygous male and female mice, using fluorogenic substrate, 4-MU NANA, in the absence and in the presence of the NEU3/NEU4 inhibitor, C9-4BPT-DANA. Residual NEU1 activity was reduced to below detection levels in all studied tissues, except for the brain, where the NEU1 activity was reduced in *Neu1^ΔEx3^* but not in *Neu1^Cx3cr1ΔEx3^* mice. *P* values were calculated using 1-way ANOVA with Dunnett’s post hoc test. (**B**) mRNA levels of *Neu1*, *Neu2*, *Neu3*, and *Neu4* were measured in the kidneys of 3 mice per genotype using quantitative reverse transcription PCR (RT-PCR). (**C** and **D**) Elevated levels of lysosomal β-galactosidase and β-hexosaminidase activities, characteristic of increased lysosomal biogenesis, were found in all studied tissues of *Neu1^ΔEx3^* mice as well as in the kidney, spleen, lungs, and brain of female *Neu1^Cx3cr1ΔEx3^* mice and showed a trend toward an increase in the tissues of males. *P* values were calculated using 1-way ANOVA with Tukey’s post hoc test. All graphs show individual data, means, and SD of experiments performed using tissues from 5 mice per genotype. (**E**) Increased levels of lysosomal proteins in kidney of *Neu1^ΔEx3^* mice. Bar graph shows exclusive unique peptide counts for 15 most abundant lysosomal proteins. Proteomic analyses were performed using kidney protein extracts from 3 mice per sex per genotype. *P* values for the exclusive unique peptide counts were calculated using 2-way ANOVA with Holm-Šídák post hoc test. (**F**) Immunohistochemical analysis shows increased TFEB levels (shown in green) in the nuclei of endothelial cells in proximal tubules of *Neu1^ΔEx3^* mice. DAPI (blue) was used as nuclear counterstain. Bar graph shows quantification (individual data, means, and SD, *n* = 3) of TFEB/DAPI-labeled areas by ImageJ software (NIH). *P* values were calculated by unpaired 2-tailed *t* test.

Total acidic neuraminidase (NEU1, NEU3, and NEU4 together), specific NEU1, total β-hexosaminidase, and acidic β-galactosidase enzyme activities were measured with fluorogenic 4-methylumbelliferyl (4-MU) substrates in the homogenates of kidney, liver, spleen, lungs, and brain of WT, *Neu1*^ΔEx3^, and *Neu1*^Cx3cr1*Δ*Ex3^ mice. NEU1 enzymatic activity, measured in the presence of a specific NEU3/NEU4 inhibitor, C9-4BPT-DANA (31), was below detection limit in tissues of *Neu1*^ΔEx3^ mice (Figure 2A). Surprisingly, *Neu1*^Cx3cr1*Δ*Ex3^ mice also showed similarly reduced NEU1 activity levels in all studied tissues except for the brain, where the activity was reduced to ~30%–50% of normal, and liver (~20% of normal, Figure 2A). By comparing levels of total neuraminidase and NEU1 activity, we conclude that NEU1 is the major (>90%) source of neuraminidase activity in the kidney. NEU1 is a relatively minor component in the spleen (<20% of total), as well as in the brain, lungs, and liver (~30% of total neuraminidase activity). Notably, mRNA levels for *Neu2*–*Neu4* isoenzymes were similar in the kidney of *Neu1*^ΔEx3^ and WT mice, indicating that genetic depletion of *Neu1* did not cause changes in the expression of other neuraminidases (Figure 2B).

Activities of nontargeted lysosomal glycosidases (β-hexosaminidases A and B, and β-galactosidase) showed a significant increase in all tissues of *Neu1*^ΔEx3^ mice except for the liver, where total β-hexosaminidase activity showed a trend for an increase (Figure 2, C and D). These effects are expected and result from lysosomal storage and elevated lysosomal biogenesis. Lysosomal β-galactosidase and β-hexosaminidase enzyme activities were significantly increased in kidney, lungs, spleen, and brain of *Neu1*^Cx3cr1*Δ*Ex3^ female mice. Lysosomal β-galactosidase activity was also significantly increased in the spleen of male *Neu1*^Cx3cr1*Δ*Ex3^ mice, while lysosomal β-hexosaminidase activity was elevated in kidney. In the other tissues of male *Neu1*^Cx3cr1*Δ*Ex3^ mice, both enzymes showed only a trend for increased activity due to wider variations between individual mice (Figure 2, C and D). Drastically increased levels of lysosome-associated membrane protein 1 (LAMP-1) in the tissues of both *Neu1*^ΔEx3^ and *Neu1*^Cx3cr1*Δ*Ex3^ mice, revealed by immunochemistry (Supplemental Figure 3), were also suggestive of increased lysosomal biogenesis.

To evaluate this further, we conducted a nontargeted proteomics analysis of kidney tissues of 3 male and 3 female 4-month-old *Neu1*^ΔEx3^ and WT mice. Prior to the tryptic digestion, protein samples were treated or not with peptide:N-glycosidase F (PNGaseF) to reveal peptides potentially bearing N-linked glycan chains. The immunoblot analysis of untreated and PNGaseF-treated protein extracts, using anti–LAMP-1 antibodies (Supplemental Figure 4), revealed that in the treated samples from both WT and *Neu1*^ΔEx3^ mice the LAMP-1–immunoreactive band showed a positive electrophoretic mobility shift corresponding to a difference in size of –25 kDa as compared with untreated samples, consistent with a removal of N-linked glycan chains (32). The same analysis also verified a ~5-fold increase in LAMP-1 band intensity (Supplemental Figure 4). The liquid chromatography-tandem mass spectrometry (LC-MS/MS) analysis identified 1,841 proteins in the kidney extracts (FDR ≤ 1%). A total of 101 proteins were absent or reduced in female and 75 in male *Neu1*^ΔEx3^ mice, whereas 150 proteins were increased or present only in female and 243 in male *Neu1*^ΔEx3^ mice compared with WT (Supplemental Figure 4 and Supplemental Table 2). These proteins were classified according to their biological function and linked to a particular metabolic or signaling pathway using automated Gene Ontology (GO) terms annotation (Supplemental Figure 4) (33). The group with the main increase in the kidney of *Neu1*^ΔEx3^ mice contained lysosomal soluble and membrane proteins (25% of increased proteins in female and 16% in male mice, 101 proteins in total; Figure 2E, Supplemental Figure 4, and Supplemental Table 2), consistent with induced lysosomal biogenesis. To test this further, we analyzed levels and localization of transcription factor EB (TFEB) protein, the master regulator of lysosomal gene expression (reviewed in ref. 34), by immunofluorescence microscopy (Figure 2F). These experiments revealed significantly increased levels of TFEB in the nuclei of endothelial cells of proximal tubules, the phenomenon reported for multiple tissues with lysosomal storage and known to cause increased expression of the lysosomal genes.

### Neu1^ΔEx3^ and Neu1^Cx3cr1ΔEx3^ mice show prominent tubuloglomerular pathology.

**Figure 3:**
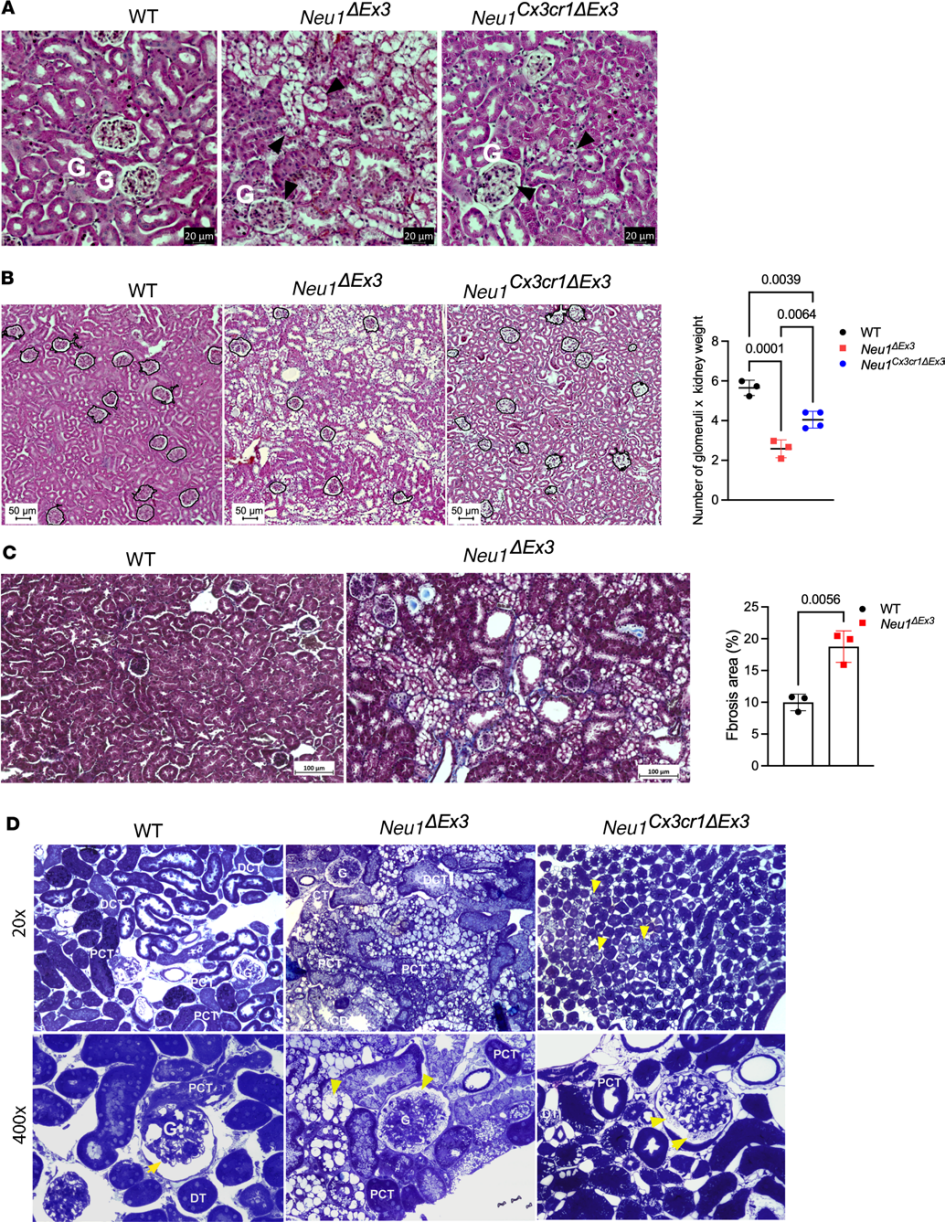
Light microscopy images of cortical and medullary regions of kidney from WT, *Neu1^ΔEx3^*, and *Neu1^Cx3cr1ΔEx3^* mice stained with H&E, Masson’s trichrome, and toluidine blue. (**A**) Normal glomeruli (G) and renal tubular structures are observed in the kidneys of WT mice. In *Neu1^ΔEx3^* kidney, severe accumulation of storage materials is present in the glomerular cells, and in surrounding tubules (black arrowheads), leading to morphological changes. Deformed tubules with vacuolized epithelial cells are also present in the kidney of *Neu1^Cx3cr1ΔEx3^* mice. Scale bar: 20 μm. (**B**) A significant loss of nephrons was observed in the kidney cortex of 4-month-old *Neu1^ΔEx3^* mice. Panels show representative images with nephrons circled, and the graph shows individual values (number of nephrons/regions of interest selected at the same positions from the cortex and multiplied by the kidney weight to account for kidney enlargement occurring in NEU1-deficient mice due to lysosomal storage), means, and SD obtained from 3 WT, 3 *Neu1^ΔEx3^*, and 4 *Neu1^Cx3cr1ΔEx3^* male and female mice. *P* values were calculated using 1-way ANOVA with Tukey’s post hoc test. Scale bar: 50 μm. (**C**) Masson’s trichrome staining reveals collagen deposits (blue) in the tubulointerstitial areas and parietal epithelium of the Bowman’s capsule in *Neu1^ΔEx3^* mouse characteristic of renal fibrosis. Scale bar: 100 μm. (**D**) WT mouse kidney have normal morphology and do not present buildup of lysosomal vacuoles in intraglomerular cells (G), proximal tubular cells (PT), distal tubular cells (DT), and cells of collecting ducts (CD). Conversely, the kidney of *Neu1^ΔEx3^* mice show a prominent accumulation of vacuoles in intraglomerular cells (G), and, presumably, podocytes (yellow arrow). The epithelial cells of DCT and CD exhibit a prominent accumulation of vacuoles. In *Neu1^Cx3cr1ΔEx33^* mice, both the cortex and medulla were mildly affected. In the cortex, both intraglomerular cells and DT show a moderate accumulation of lysosomal vacuoles. In the medulla, the descending portions of the loop of Henle are normal. PCT, proximal convoluted tubule; DCT, distal convoluted tubule.

Our further analysis was focused on kidney pathology to provide insights into the mechanism underlying kidney dysfunction in patients with nephrosialidosis. We first conducted light microscopy examination of sagittal H&E-stained kidney sections, which showed several pathological changes in kidneys of both *Neu1*^ΔEx3^ and *Neu1*^Cx3cr1*Δ*Ex3^ mice: deformed tubules, vacuolized endothelial cells, and condensed glomeruli (Figure 3A). However, we did not observe bilateral hydronephrosis or other signs of acute kidney injury that would be expected to result from the urinary retention occurring in both *Neu1*^ΔEx3^ and *Neu1*^Cx3cr1*Δ*Ex3^ strains. The analysis also revealed a significant (by a factor of 60%–70%) reduction in the number of glomeruli in *Neu1*-null mice (Figure 3B). Surprisingly, this dramatic change was not accompanied by a drastic decline of renal function since serum creatinine levels were similar. We reasoned that our calculations were likely biased because *Neu1*^ΔEx3^ mice are smaller than their WT littermates, while they have larger kidneys. This latter phenomenon is largely due to the presence of storage materials throughout the kidneys, which increases kidney size without parallel scaling of the number of glomeruli. Thus, we first approximated the overall number of glomeruli for each kidney (by multiplying the average number of glomeruli by the kidney weight); then, we adjusted for discrepant body sizes by dividing this value by the mouse body weight at sacrifice. After these normalizations, the number of glomeruli was only mildly reduced in *Neu1*-null mice (WT 1.00 ± 0.061; *Neu1*^ΔEx3^ 0.796 ± 0.053; *P* = 0.034) but not in the conditional KO strain. Also, Masson’s trichrome staining detected mild-to-moderate collagen deposition in the tubulointerstitial areas and parietal epithelium of the Bowman’s capsule indicative of renal fibrosis (Figure 3C).

To identify specific structural abnormalities, we studied semithin kidney sections, stained with toluidine blue, by high-resolution light microscopy, as well as thin sections, contrasted with uranyl acetate, by transmission electron microscopy (TEM). Toluidine blue–stained sections showed a severe buildup of lysosomal vacuoles in the intraglomerular region and in the epithelial cells of proximal and distal convoluted tubules in *Neu1*^ΔEx3^ mice, which was not detected in the WT controls (Figure 3D). In *Neu1*^Cx3cr1*Δ*Ex3^ mice, glomerular cells were similarly affected; however, in contrast with *Neu1*^ΔEx3^ mice, only the DCTs showed moderate accumulation of lysosomal vacuoles, while the PCTs and loop of Henle in the medulla showed no signs of vacuolization (Figure 3D).

**Figure 4:**
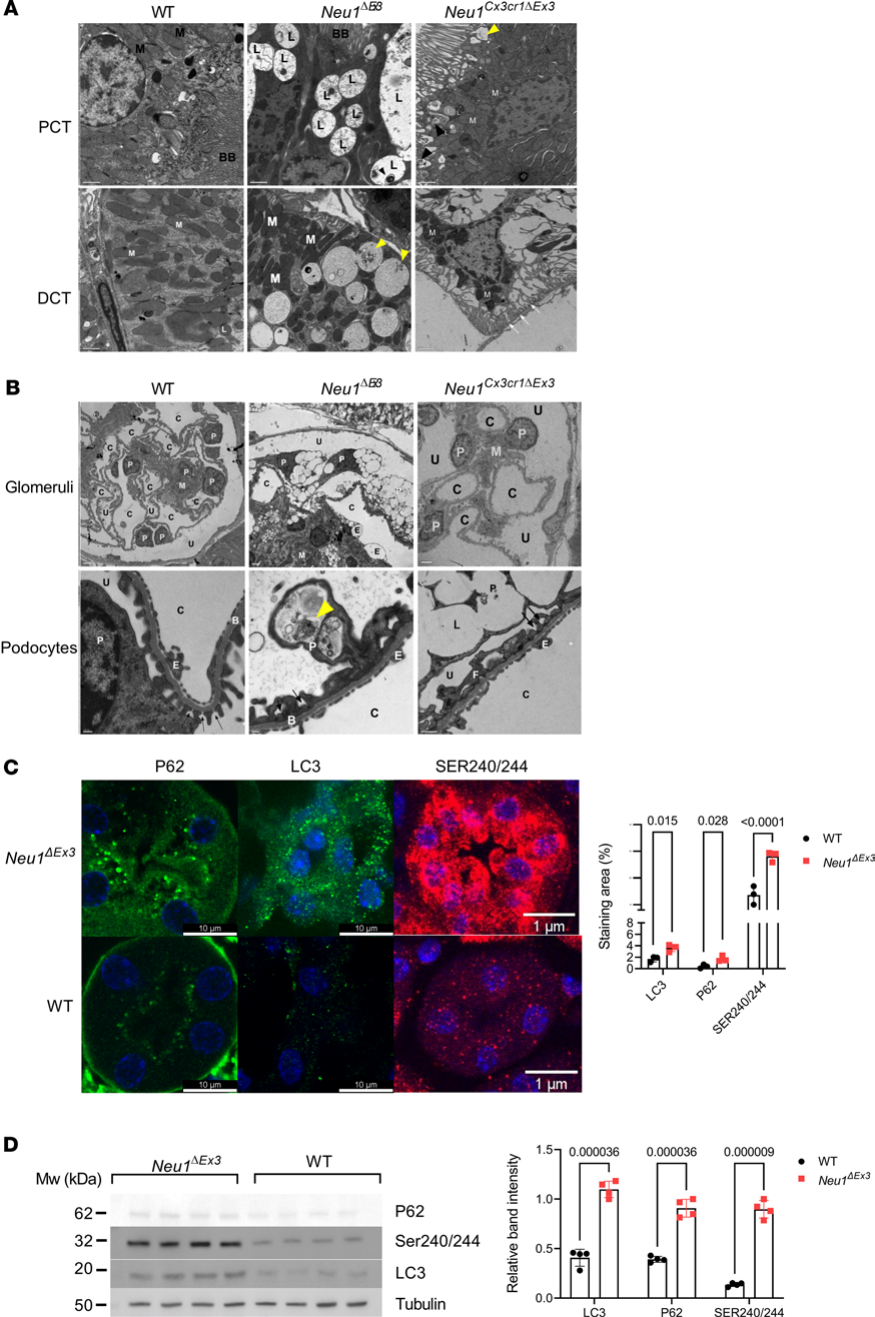
Pathological changes in glomerular and tubular cells in *Neu1*^ΔEx3^ and *Neu1*^Cx3cr1*Δ*Ex3^ mice. (**A**) Kidneys of *Neu1^ΔEx3^* mice contain numerous enlarged electron-lucent lysosomes (L) filled with multilamellar structures (black arrowhead) in the proximal convoluted tubule (PCT) and multivesicular bodies (yellow arrowheads) in the lysosomal compartments of the distal convoluted tubules (DCT). Small irregularly shaped mitochondria with fragmented cristae (M) are found throughout the PCT and DCT of *Neu1^ΔEx3^* and *Neu1^Cx3cr1ΔEx3^* mice. In the DCT of *Neu1^Cx3cr1ΔEx3^* mice, mitochondria are dissociated from the distorted basolateral plasma membrane (white arrows). (**B**) In the glomeruli of *Neu1^ΔEx3^* mice, the podocytes (P) and mesangial cells (M) are severely vacuolated. Higher magnification of podocytes shows multivesicular structures and osmiophilic deposits (yellow arrowhead). The podocyte foot processes (F), which form a discontinuous lining for the inner aspect of the WT glomerular basement membrane, are widely effaced in both *Neu1^ΔEx3^* and *Neu1^Cx3cr1ΔEx3^* mice (black arrows). All transmission electron microscopy panels show representative images taken for 3 WT, 3 *Neu1^ΔEx3^*, and 2 *Neu1^Cx3cr1ΔEx3^* mice. Scale bars equal 1 μm (**A**), 2 μm in glomeruli and 0.2 μm in high-magnification images of podocytes (**B**). C, capillaries; U, urinary space; E, endothelium. (**C**) Endothelial cells of the proximal convoluted renal tubules in *Neu1^ΔEx3^* mice reveal accumulation of P62^+^ and LC3^+^ puncta, consistent with impaired autophagy and increased phosphorylation (Ser240/244) of S6 ribosomal protein substrate of mTOR complex 1 (mTORC1). Scale bars: 10 μm (left and middle), 1 μm (right). Graph shows relative areas stained with antibodies against P62, LC3, and Ser240/244 S6 quantified with ImageJ. Individual values, means, and SD are shown (*n* = 3). *P* values were calculated with multiple unpaired 2-tailed *t* tests. (**D**) Immunoblotting of kidney proteins verifies increase in P62, LC3, and Ser240/244 levels. Graph shows bands intensities, quantified with ImageJ software and normalized to the intensities of tubulin immunoreactive bands. Individual values, means, and SD are shown (*n* = 3). *P* values were calculated with multiple unpaired 2-tailed *t* tests.

Further examination of kidney cortices by TEM verified the existence of severe structural defects in the renal tubules and glomeruli. In *Neu1*^ΔEx3^ mice, the PCT, identified by the presence of the brush border (BB), displayed numerous lysosomal vacuoles containing multilamellated structures (Figure 4A). The PCT of *Neu1*^Cx3cr1*Δ*Ex3^ mice displayed mitochondria with fragmented cristae and vacuoles with multilamellar structures adjacent to the BB. However, in contrast to the PCT of *Neu1*^ΔEx3^ mice, no enlarged lysosomes were observed in the PCT of *Neu1*^Cx3cr1*Δ*Ex3^ mice. DCTs in the *Neu1*^ΔEx3^ mice showed multiple electron-lucent lysosomes with multivesicular bodies (Figure 4A). The mitochondria in the DCTs of *Neu1*^ΔEx3^ mice were pleomorphic, small, and disorganized with absent cristae, while in the *Neu1*^Cx3cr1*Δ*Ex3^ mice, numerous mitochondria were detached from the basolateral membrane.

Severe pathological changes were also detected in the glomeruli of *Neu1*^ΔEx3^ mice, which had multiple lysosomes located throughout the cell body of podocytes, mesangial cells, and glomerular parietal epithelial cells (Figure 4B). In the *Neu1*^Cx3cr1*Δ*Ex3^ kidney, most glomeruli appeared healthy, but some showed a pathology similar to that seen in the *Neu1*^ΔEx3^ mice, with vacuolation of the mesangial cells and podocytes. At higher magnification, multivesicular bodies and some osmiophilic structures, resembling lipid or protein aggregates, were detected in the lysosomal compartments of *Neu1*^ΔEx3^ podocytes. In contrast, *Neu1*^Cx3cr1*Δ*Ex3^ podocytes exhibited multiple clear, fused vacuoles. In both *Neu1*^ΔEx3^ and *Neu1*^Cx3cr1*Δ*Ex3^ glomeruli, the filtration slits between podocyte foot processes (pedicles) displayed effacement that was absent in WT kidneys (Figure 4B). Glomerular endothelial cells appeared grossly normal, with normal fenestrations.

Since the presence of pleomorphic mitochondria and vacuoles with the content resembling secondary storage materials of protein and lipid nature in glomerular and tubular cells was consistent with an autophagy block, we have analyzed kidney tissues for the presence of puncta positive for autophagosome proteins P62 and LC3 (Figure 4C). While in the tissues of WT mice both proteins were not detected, consistent with normal autophagy flux, in the endothelial cells of proximal tubules of *Neu1*^ΔEx3^ mice, we observed coarse LC3-positive and P62-positive puncta indicative of autophagy block caused by inability of autophagosomes to fuse with lysosomes (35). The increased LC3 and P62 levels were confirmed by immunoblotting of total kidney proteins (Figure 4D). Both immunofluorescence microscopy and immunoblot experiments also revealed increased levels of phosphorylated (S240/S244) S6 ribosomal protein, the substrate of mTORC1, a key regulator of autophagy and lysosome biogenesis (Figure 4, C and D). This indicated that mTORC1 activity against this substrate was increased in kidney of *Neu1*^ΔEx3^ mice and, specifically, in the cells of proximal tubules.

Since tissue infiltration with immune cells was previously reported for patients with sialidosis, kidney tissues were examined by immunohistochemistry for the presence of CD68-positive macrophages. Our results demonstrated that the kidneys of both *Neu1*^ΔEx3^ and *Neu1*^Cx3cr1*Δ*Ex3^ mice exhibited high numbers of CD68-positive cells, whereas none were detectable in WT controls (Supplemental Figure 3). Importantly, in mice of both strains, macrophages showed an amoeba-like morphology that is typical for activated cells.

The extent of kidney tissue abnormalities in *Neu1*^ΔEx3^ and *Neu1*^Cx3cr1*Δ*Ex3^ mice revealed by light microscopy and TEM suggested that it would likely be accompanied by abnormalities in kidney function(s). To verify proteinuria was present, urine samples, collected from 4-month-old WT, *Neu1*^ΔEx3^, and *Neu1*^Cx3cr1*Δ*Ex3^ mice using metabolic cages, were analyzed by SDS-PAGE. The gels showed a prominent protein 65 kDa band, characteristic of albumin, in the urine of *Neu1*^ΔEx3^ mice. This band was absent in the urine of age- and sex-matched WT and *Neu1*^Cx3cr1*Δ*Ex3^ mice (Supplemental Figure 5A). Analysis with a urine dipstick also revealed increased protein and glucose levels and reduction of pH in the urine of *Neu1*^ΔEx3^ mice (Supplemental Figure 5B). At the same time, the specific gravity of urine of *Neu1*^ΔEx3^ and *Neu1*^Cx3cr1*Δ*Ex3^ mice was in the normal range (1.030, similar to that of WT mice), indicating that these mice did not develop a urinary concentrating defect with polyuria (Supplemental Figure 5B). To test whether glucosuria was associated with glycemia, we measured blood glucose levels in 4-month-old, fasted mice of both strains and detected elevated glucose levels in *Neu1*^ΔEx3^ mice, and in male, but not in female, *Neu1*^Cx3cr1*Δ*Ex3^ mice (Supplemental Figure 5C). Finally, ELISA test verified increased urine albumin levels and increased urine albumin-to-creatinine ratio (UACR) levels in 4-month-old *Neu1*^ΔEx3^ and 5-month-old *Neu1*^Cx3cr1*Δ*Ex3^ mice (Supplemental Figure 5D).

### Glycomic profiling reveals protein hypersialylation in Neu1^ΔEx3^ mouse kidneys.

**Figure 5:**
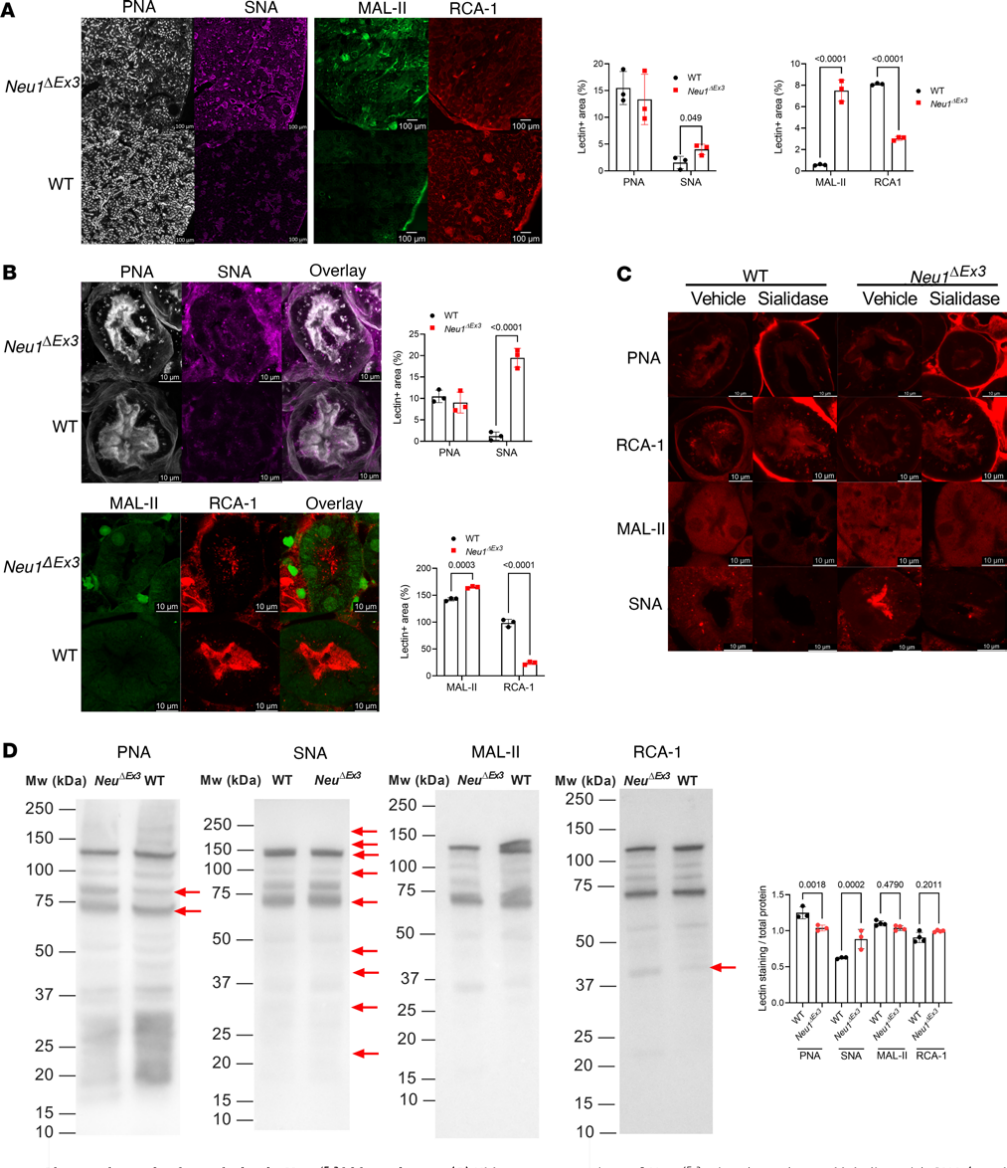
Abnormal protein glycosylation in *Neu1*^ΔEx3^ kidney tissues. (**A**) Kidney cortex sections of *Neu1^ΔEx3^* mice show elevated labeling with SNA (purple) and MAL-II (green) lectins and reduced labeling with RCA-1 (red) lectin. PNA labeling (white) shows a nonsignificant trend toward a decrease. (**B**) SNA (purple) and MAL-II (green) labeling is drastically increased in the proximal convoluted renal tubules of *Neu1^ΔEx3^* mice, RCA-1 labeling (red) shows a decrease and PNA labeling (white) a nonsignificant trend toward a decrease. (**C**) Treatment of kidney tissues with exogenous pan-specific bacterial *Arthrobacter ureafaciens* sialidase increases PNA and RCA-1 labeling and reduces MAL-II and SNA labeling in the proximal convoluted renal tubules of both *Neu1^ΔEx3^* and WT mice, confirming specificity of the assay. Images were taken with Leica confocal microscope SP8-DLS. Scale bars equal 100 μm (**A**) and 10 μm (**B** and **C**). Graphs show lectin-positive areas (individual values, means, and SD, *n* = 3). Quantifications were performed by ImageJ software, and *P* values were calculated using multiple 2-tailed *t* tests. (**D**) Lectin blotting of kidney proteins shows changes in glycosylation of proteins in tissues of *Neu1^ΔEx3^* mice compared with WT mice. Panels show images of representative lectin blots. Red arrows mark protein bands with decreased affinity for PNA and increased affinity for SNA. Graphs show combined intensities (individual values, means, and SD) of protein bands stained with lectins and normalized by combined intensities of Ponceau staining. Quantifications were performed by ImageJ software, and *P* values were calculated using a 2-tailed *t* test.

To test whether NEU1 deficiency altered protein sialylation in kidney tissues, we stained kidney sections with fluorescently labeled *Sambucus nigra* lectin (SNA) that binds preferentially to sialic acids attached to terminal galactose in α-2,6 and, to a lesser degree, α-2,3 position. We also used *Maackia amurensis* lectin 2–4 (MAL-II), specific for α-2,3–linked Sia residues; *Arachis hypogaea* (peanut) agglutinin (PNA), specific for terminal β-galactose residues in O-linked glycans; and *Ricinus communis* agglutinin I (RCA-1), specific for terminal β-galactose residues in N-linked glycans. The labeling with SNA and MAL-II was drastically increased throughout the kidney and specifically in the proximal renal tubules of *Neu1*^ΔEx3^ compared with WT mice (Figure 5, A and B). In contrast, labeling with RCA-1, usually exposed in complex glycans, after neuraminidase treatment, was reduced, while PNA labeling showed a nonsignificant trend toward reduction (Figure 5, A and B). Treatment of kidney tissues with exogenous pan-specific *Arthrobacter ureafaciens* sialidase increased PNA and RCA-1 labeling and reduced MAL-II and SNA labeling in the proximal convoluted renal tubules of both *Neu1*^ΔEx3^ and WT mice, verifying specificity of the assay (Figure 5C). These results, suggesting hypersialylation of kidney tissues in *Neu1*^ΔEx3^ mice, were partially verified by lectin blotting, indicating that multiple proteins extracted from *Neu1*^ΔEx3^ mice kidneys had increased affinity to SNA and decreased affinity to PNA (Figure 5D). Intensity of MAL-II or RCA-1 labeling of total kidney proteins was not significantly different for WT and *Neu1*^ΔEx3^ mice.

To verify the results of lectin staining and to compare structures of protein N-glycans in WT and NEU1-deficient mice, we analyzed the N-glycome of kidney tissues from 4-month-old WT and *Neu1*^ΔEx3^ mice. The first line of matrix-assisted laser desorption/ionization time-of-flight mass spectrometry (MALDI-TOF MS) examination revealed the occurrence of sialylated glycans in tissues of *Neu1*^ΔEx3^ mice that were absent in WT counterparts (Supplemental Figure 6, A and B, and Supplemental Table 3). These species, most likely, represent free oligosaccharides stored in the lysosomes of kidney tissues or secreted in the urine of *Neu1*-KO mice, since their corresponding human analogs were frequently identified in the urine of patients with sialidosis (36). Besides, the major structure at *m/z* 2,607.2, analyzed by MALDI-TOF MS/MS (Supplemental Figure 6C), showed the presence of a single N-acetylglucosamine (GlcNAc) at the reducing glycan end, instead of the chitobiose disaccharide GlcNAc-GlcNAc, which is expected to be found in N-linked glycans.

**Figure 6:**
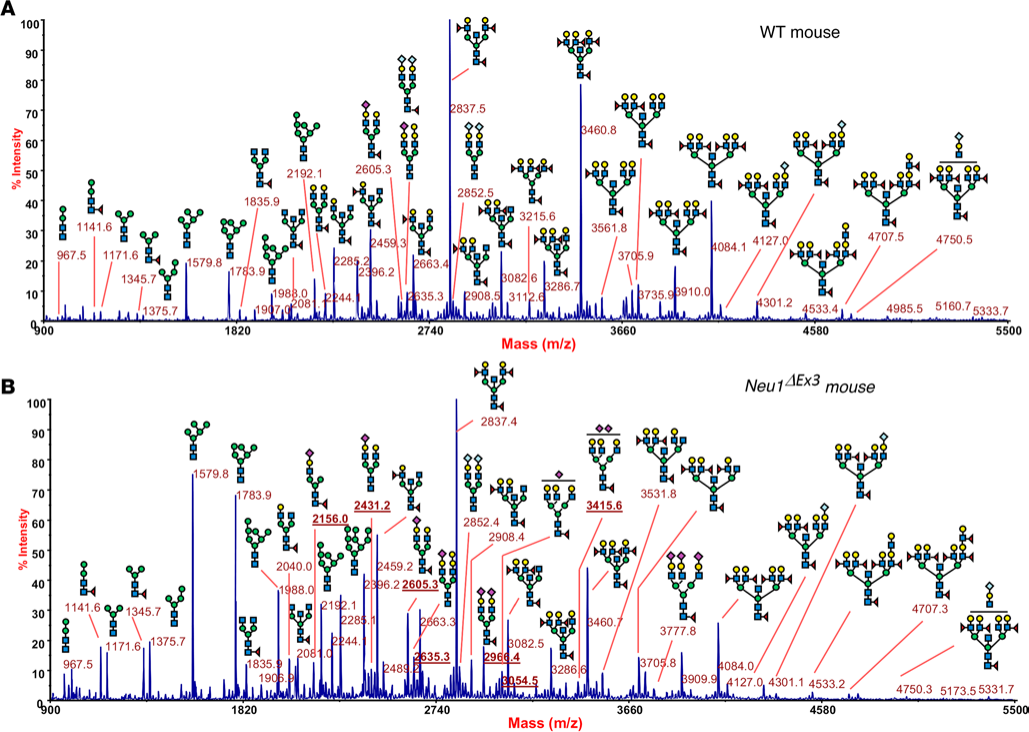
MALDI-TOF MS analysis of mouse kidney proteins shows changes in the profile of N-glycans. MALDI-TOF profiles (mass range between *m/z* 900 and 5,500) of permethylated N-glycans from kidney tissue glycoproteins representative for samples from WT (**A**) and *Neu1^ΔEx3^* (**B**) female mice showing increased amounts of sialylated structures (underlined *m/z* values). Structures of the glycan species were corroborated by MS/MS analyses. Species were detected as [M+Na]^+^ molecular ions (monoisotopic masses). Graphical representation of glycans is based on the third edition of the *Essentials of Glycobiology* (74): GlcNAc, blue square; mannose, green circle; galactose, yellow circle; Neu5Ac, purple diamond; Neu5Gc, light blue diamond; fucose, red triangle.

Thus, kidney protein pellets were additionally washed with deionized distilled H_2_O to remove soluble free oligosaccharides (further studied separately by MS and MS/MS, Supplemental Table 3), and the N-glycome analyses were repeated. MALDI-TOF MS profiles of permethylated N-glycans of proteins from WT mice (Figure 6A) were essentially similar to the profiles from unwashed tissues. They presented 3 major peaks at *m/z* 2,837.5, 3,460.8, and 4,084.1, corresponding to bisected, core-fucosylated N-glycan structures bearing 2, 3, and 4 antennae, respectively, each terminated with a Lewis-X epitope, as verified by MALDI-TOF MS/MS fragmentation analysis. Minor peaks corresponded to paucimannose, oligomannose, and core-fucosylated bisected structures, the latter being predominantly terminated with galactose. Sialylated glycoforms were found in traces and were assigned at lower molecular weight to biantennary structures bearing Neu5Gc or Neu5Ac (e.g., *m/z* 2,605.3, 2,635.3, 2,852.4, and 2,966.4) and over *m/z* 4,000 to bisected structures whose antennae were mainly terminated with Lewis-X motifs or Neu5Gc (e.g., *m/z* 4,127.0 and 4,301.2). The relative intensities of N-linked glycans were changed in *Neu1*^ΔEx3^ compared with WT mice (Figure 6B). In particular, we observed a drastic accumulation of paucimannose and oligomannose structures and occurrence/increase of sialylated, not bisected, complex N-glycans bearing Neu5Ac (Figure 6B, underlined *m/z* values; e.g., 2,156, 2,431.2, 2,605.3, 2,635.3, 2,966.4, 3,054.5, and 3,415.6). Importantly, MALDI-TOF MS profiles obtained from male and female *Neu1*^ΔEx3^ mice were clearly distinguishable, with males showing an intense peak at *m/z* 2,852.4 (corresponding to a biantennary disialo-glycoform bearing 1 Neu5Gc at each antenna), which occurred to a much lesser extent in female littermates (Supplemental Figure 7). Table 1 and Supplemental Table 4 list all the sialylated glycoforms accumulated in *Neu1*^ΔEx3^ kidney tissues and provide a relative quantitative comparison between WT and *Neu1*^ΔEx3^ mice. To analyze sialic acid linkages, we also performed N-glycoprofiling by HILIC-UPLC-FLR-ESI-MS (37) capable of distinguishing between α2,3- and α2,6-linked sialic acids (38). These analyses verified major increases of oligomannose and sialylated N-glycans in *Neu1*^ΔEx3^ mice (Supplemental Figure 8A) and indicated that the ratio of α2,3- to α2,6-linked sialic acids was similar in WT and in *Neu1*^ΔEx3^ mice, as illustrated by the representative extracted ion current for the disialo-biantennary core-fucosylated structure with *m/z* 1,341.0150 (Supplemental Figure 8B).

### Aberrant glycosylation and trafficking of endocytic receptor megalin in kidney cells of Neu1^ΔEx3^ mice.

An endocytic receptor megalin (lipoprotein receptor-related protein 2, LRP2), responsible for the uptake of numerous urinary metabolites, is one of the main glycoproteins on apical membranes of proximal tubules. This protein bears multiple O-linked and N-linked glycans with terminal β-galactose residues, which are essential for the intracellular stability of LRP receptors (39). Glycosylation defects of megalin are known to affect its function and expression, leading to kidney dysfunction and proteinuria (40).

**Figure 7:**
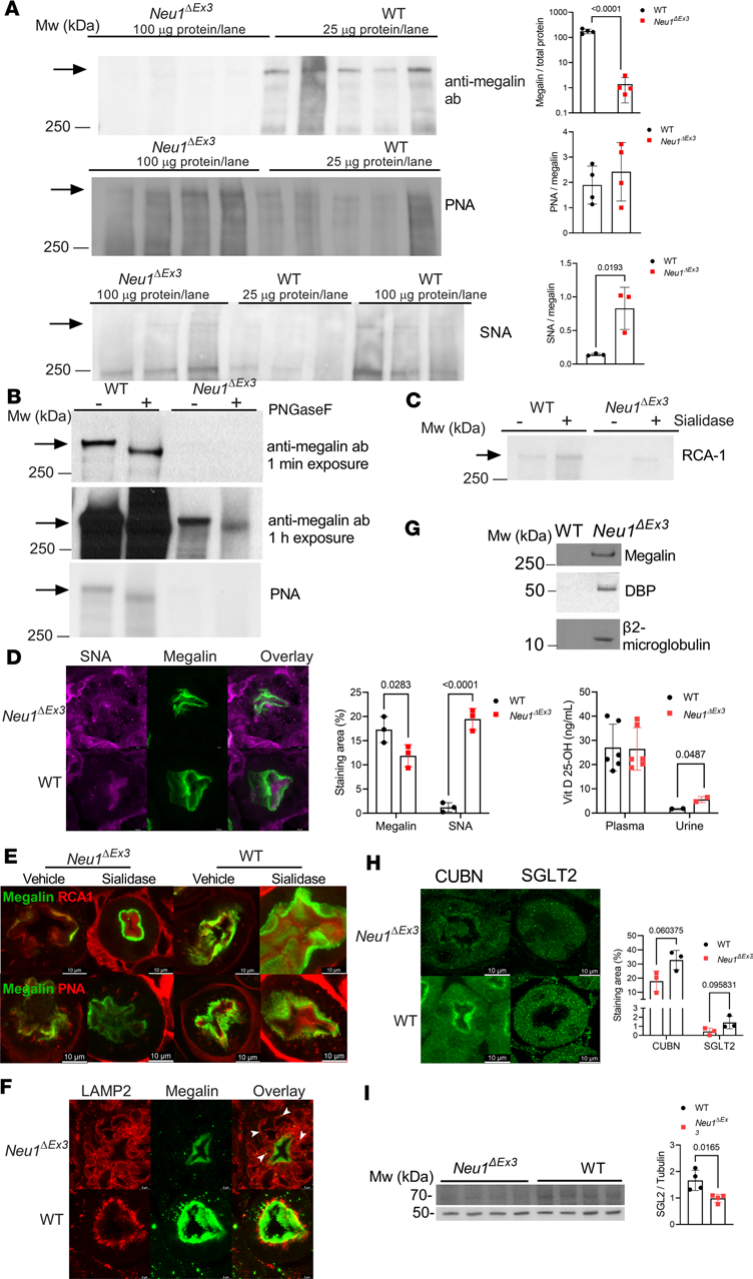
Aberrant glycosylation of megalin affects its abundance and trafficking in the kidney of *Neu1*^ΔEx3^ mice. (**A**) Immunoblot shows reduction of megalin in *Neu1^ΔEx3^* mouse kidney. Lectin blots show that megalin affinity to SNA substantially increases, suggestive of protein hypersialylation. A total of 100 or 25 μg of kidney protein extract from *Neu1^ΔEx3^* and WT mice was analyzed. Arrows mark megalin position. (**B**) In the WT kidney, megalin shows equal intensity of PNA staining, before and after PNGaseF treatment, suggesting that the protein contains mainly O-linked glycans with terminal galactose residues. In *Neu1^ΔEx3^* kidney, the PNGaseF-treated protein does not show affinity to PNA, suggesting the absence of glycans with terminal galactose. (**C**) Megalin in WT kidney is recognized by RCA-1 specific for N-linked glycans with terminal galactose residues. In *Neu1^ΔEx3^* kidney, megalin is recognized by RCA-1 only after treatment with bacterial sialidase, consistent with oversialylation masking galactose residues. (**D**) In proximal renal tubules of *Neu1^ΔEx3^* kidney, megalin (green) colocalizes with SNA (magenta), suggesting its hypersialylation. 3D images were acquired using SP8-DLS high-resolution confocal microscope. Colocalization of megalin and SNA was analyzed by LasX software (Supplemental Videos 1 and 2). (**E**) RCA-1 colocalizes with megalin in WT but not in *Neu1^ΔEx3^* kidney. RCA-1 and PNA staining is increased after sialidase treatment. (**F**) In proximal tubules of WT kidney, megalin (green) is found on the apical membrane; in *Neu1^ΔEx3^* kidney, it is found inside enlarged LAMP2^+^ lysosomes (white arrows and Supplemental Videos 3 and 4). (**G**) Megalin, β2-microglobulin (β2-MG), vitamin D–binding protein (DBP), and 25-OH vitamin D are detected in urine of *Neu1^ΔEx3^* mice. (**H**) Cubilin (CUBN) and solute carrier SGLT2 show a trend toward reduction on the apical surface of the proximal tubules of *Neu1^ΔEx3^* kidney. (**I**) Immunoblotting verifies reduction of SGLT2 protein in *Neu1^ΔEx3^* kidney homogenates. Fluorescence and band intensities were quantified with ImageJ software. Microphotographs in **D** and **F** were taken at 630× original magnification with 5× zoom. Individual data, means, and SD (*n* = 3) are shown. *P* values were calculated using unpaired multiple *t* test.

Since the presence of albumin in the urine of *Neu1*^ΔEx3^ mice suggested a reabsorption defect potentially related to deficiencies of megalin expression or processing, we analyzed levels of megalin in kidney tissues by immunoblotting and observed drastically decreased levels of megalin in tissues of *Neu1*^ΔEx3^ compared with WT mice (Figure 7A). To understand whether reduced renal levels of megalin in *Neu1*^ΔEx3^ mice are associated with changes in glycosylation, we performed PNA and SNA lectin blots and determined significantly increased affinity of the megalin band for SNA consistent with oversialylation, while PNA labeling of the protein was not changed. However, when we repeated PNA blot with the samples pretreated with endoglycosidase PNGaseF to remove N-linked glycans, we observed that megalin-immunoreactive protein bands in WT kidney extracts, either untreated or treated with PNGaseF, were equally stained with PNA. These results are consistent with O-linked glycans with terminal β-galactose residues being the major glycoform recognized by PNA on megalin in murine kidneys (Figure 7B). In contrast, in *Neu1*^ΔEx3^ kidney extracts, the megalin band was not stained with PNA after PNGaseF treatment, suggesting that the β-galactose residues on O-linked glycans were now masked by sialylation (Figure 7B). In the untreated samples from WT kidney, the megalin band was recognized by RCA-1, and the labeling intensity was further increased after the treatment with *Arthrobacter ureafaciens* sialidase. In samples from *Neu1*^ΔEx3^ kidney, the megalin band was recognized by RCA-1 only in sialidase-treated samples, consistent with oversialylation of N-linked glycan chains (Figure 7C).

To validate this further, we conducted analysis of proximal tubule in kidney tissues of WT and *Neu1*^ΔEx3^ mice, costained with anti-megalin antibodies and either SNA (for sialic acid) or PNA (for galactose), by high-resolution SP8-DLS confocal microscopy (Figure 7D). 3D confocal images rendered with the LasX program showed the proximity of the SNA signals and anti-megalin antibodies in the *Neu1*^ΔEx3^ tissues, indicating that megalin was sialylated (Figure 7D and Supplemental Videos 1 and 2). In contrast, in WT tissues, colocalization was not observed for anti-megalin antibodies and SNA, suggesting that megalin was mainly present in its asialo form. In addition, analysis of tissues labeled with RCA-1 or PNA lectins and anti-megalin antibodies showed a reduced intensity of RCA-1 and PNA labeling colocalizing with megalin in proximal tubules of *Neu1*^ΔEx3^ mice compared with WT mice, which was restored by sialidase treatment (Figure 7E).

We further used high-resolution SP8-DLS confocal microscopy to analyze tissues labeled for megalin and the lysosomal marker LAMP-1 and found a drastic difference in localization of megalin in proximal tubules of WT and *Neu1*^ΔEx3^ mice. In the WT mouse kidney, the majority of megalin was, as expected, found on the apical membranes of proximal tubules. In contrast, in the *Neu1*^ΔEx3^ mouse kidney, most of the megalin protein was found inside LAMP-1–positive luminal structures, indicating its trafficking to enlarged lysosomes (Figure 7F and Supplemental Videos 3 and 4). Finally, immunoblot analysis of urine samples from *Neu1*^ΔEx3^ mice revealed high levels of megalin as well as of LMWPs that are known to be reabsorbed via binding to megalin: β2-microglobulin (β2-MG) and vitamin D–binding protein (DBP). In contrast, and as expected, urine samples from WT mice did not contain any of these proteins. This suggests that megalin sheds off apical membranes of the proximal tubule cells and that this results in defective reabsorption of LMWPs (Figure 7G). We further tested if megalin deficiency and increased urinary secretion of DBP were associated with altered levels of 25-OH vitamin D by measuring its levels in blood plasma and urine with ELISA. We found no significant difference in 25-OH vitamin D between *Neu1*-KO and WT mice in plasma; however, urinary levels were significantly increased in *Neu1*^ΔEx3^ compared with WT mice (Figure 7G).

To test whether other endocytic receptors and solute carriers, present at the apical membrane of proximal tubules, are also reduced in the *Neu1*^ΔEx3^ mouse kidney, we searched our proteomics data for the proteins associated with the corresponding GO terms and identified several proteins decreased or absent in kidney of *Neu1*^ΔEx3^ mice (Supplemental Figure 4 and Supplemental Table 2). This list of proteins included a megalin-binding endocytic receptor cubilin (*Cubn*), sodium/glucose cotransporter 2 (*Sglt2*), solute carrier family 22 member 6 (*Slc22a6*, kidney-specific organic transport protein 1), solute carrier family 22 member 12 (*Slc22a12*, urate anion exchanger 1), solute carrier family 22 member 2 (*Slc22a2*, organic cation transporter 2), and prostaglandin E_2_ receptor. Proteomics analysis also verified the reduction of megalin (*Lrp-2*) in the *Neu1*^ΔEx3^ mouse kidney (Supplemental Table 2). The reduction of CUBN and SGLT2 on the apical surface of proximal renal tubule of *Neu1*^ΔEx3^ mice was further directly verified by immunofluorescence microscopy and immunoblotting (Figure 7, H and I).

## Discussion

During the process of blood filtration through the kidney, water, solutes, and proteins pass through the filtration membrane, followed by recovery of proteins in the proximal renal tubule in a so-called renal protein reabsorption process. The process of reabsorption ensures that albumin, LMWPs, bicarbonate, glucose, phosphate, amino acids, and other key nutrients are recovered by the organism and not lost with the urine. Reclamation of albumin and LMWPs is mainly mediated by 2 endocytosis receptors, megalin (also known as LRP2) and CUBN, forming a complex at the apical membranes of epithelia (41). LRP2/CUBN receptors are found on the surface of epithelial cells in most tissues (reviewed by ref. 42) but are most highly expressed in PCTs (43, 44). Both proteins belong to the class of scavenger receptors that recognize and bind multiple ligands, including vitamins, carrier proteins, enzymes, and hormones (42). Patients with various forms of Fanconi renotubular syndrome secrete LMWPs (such as DBP, retinol-binding protein, and β2-MG) in the urine because of proximal tubular dysfunction (45, 46). Megalin-null mice have high perinatal mortality rate because of impaired renal function, respiratory complications, and holoprosencephaly (47), suggesting that the protein also plays important roles in embryonic development.

Our results demonstrate that NEU1 plays a crucial role in maintaining normal glycosylation and trafficking of megalin, essential for proper renal reabsorption process. The constitutive *Neu1*-KO mice develop kidney dysfunction, resulting in low–molecular weight proteinuria, glucosuria, and elevated UACR. These abnormalities coincide with urinary retention that most likely occurs due to acute outflow tract obstruction and requires euthanasia of the animals. *Neu1*-null males show accelerated development of urinary retention and low–molecular weight proteinuria when compared with females.

The documentation of glucosuria in *Neu1*-null animals is intriguing because it could be due to abnormal function of the sodium-glucose cotransporter SGLT2, which our study shows to be reduced in the proximal tubules of *Neu1*-KO mice. In the context of normal SGLT1 and SGLT2 functions, glucosuria will occur when serum glucose concentration is higher than the maximal glucose reabsorption capacity of the kidney (or renal glucose transport maximum). Given that *CathA^S190-Neo^* mice (galactosialidosis model), which have approximately 90% reduction in tissue NEU1 activity, develop type 2 diabetes (48, 49), we sought to document if hyperglycemia was also observed in *Neu1*-null mice. While *Neu1*-null mice did indeed have higher serum glucose than WT animals, the levels observed are not high enough to cause glucosuria (50). Thus, glucosuria observed in *Neu1*-null mice most likely occurs due to abnormal glucose reabsorption at the proximal tubule. While recent data (51) reveal that SGLT2 possesses an O-linked glycan and 2 predicted N-linked glycans, the impact of SGLT2 glycosylation on its targeting and/or ability to transport glucose has not yet been investigated.

The other notable finding documented in the urine samples of both *Neu1*-deficient strains is the low urinary pH (5.0 vs. 6.5 for controls) despite exposure to similar mouse chow and environmental conditions. These data suggest that both *Neu1*-deficient strains must have much higher acid loads when compared with WT and that tubular acid-base regulation at the proximal tubule (via bicarbonate reabsorption) and the distal tubule (via H^+^ secretion) are intact. We plan to address the etiology of the acid load in future studies.

Similar symptoms of kidney disfunction and urinary retention, although attenuated with average life span of 5 months in males and 8 months in females, were also observed in the conditional *Neu1*^Cx3cr1*Δ*Ex3^ mice, where we attempted to deplete NEU1 specifically in the phagocytic mononuclear cells. Further analysis, however, revealed that NEU1 was reduced to below detection levels in all studied tissues of *Neu1*^Cx3cr1*Δ*Ex3^ mice with an exception of liver and brain. These results suggest that inactivation of the *Neu1* gene occurs in majority of cell types, and not only in mononuclear phagocytic cells, which is consistent with the previous data, showing that the expression of *Cx3cr1* is not limited to immune cells but happens in a wide variety of tissues and organs (52). However, we also cannot exclude that NEU1 is mainly produced by tissue macrophages, followed by its exocytosis (possibly as a part of extracellular vesicles) and uptake by other types of cells such as glomerular cells and the cells of PCT and DCT of the kidney. Experiments aimed at understanding if the longer survival of *Neu1*^Cx3cr1*Δ*Ex3^ mice occurs because of the slightly higher NEU1 residual levels in all tissues or is due to the retained NEU1 activity in the brain neurons are currently underway in our lab.

Our further analyses revealed multiple structural abnormalities in the kidney tissues of both *Neu1*^ΔEx3^ and *Neu1*^Cx3cr1*Δ*Ex3^ strains, including reduced density of nephrons associated with renal fibrosis, macrophage infiltration, enlarged lysosomes containing storage materials in the PCT and DCT and podocytes, mitochondrial defects, and effaced podocyte foot processes. These pathological changes, also reported for the kidney of patients with sialidosis and for another mouse model of sialidosis (26), are thought to be caused by disruption of NEU1-mediated lysosomal catabolism of glycoproteins and sialylated oligosaccharides and their storage in lysosomes of affected tissues. Indeed, our results demonstrate that the kidney cells of *Neu1*-KO mice display a specific phenotype consisting of autophagy block, and mTORC1 hyperactivation, common for the cells with a pronounced lysosomal storage in multiple lysosomal diseases, including Gaucher, Pompe, and NPC1 (53–58). This increased activity coincides with reduced lysosomal mTORC1 (LAMTOR/Ragulator) activity against TFEB due to the structural changes within the LAMTOR complex, leading to TFEB translocation to the nuclei, drastically increased lysosomal biogenesis, and induction of multiple lysosomal proteins and enzymes. At the same time, we could not confirm a previous report describing hypersialylation of LAMP-1 in NEU1-deficient cells presumably leading to its prolonged half-life and increased lysosomal exocytosis (59).

Since previous results from our lab and others demonstrated that NEU1 is also present on the cell surface, where it is involved in trimming sialic acid residues from glycan chains of glycoproteins, we have analyzed whether protein sialylation is increased in the kidney of NEU1-deficient mice. We found increased affinity of kidney tissues of *Neu1*^ΔEx3^ mice to sialic acid–specific lectins and reduced affinity to galactose-specific lectins, suggestive of significantly increased sialylation. This was further verified by the analysis of kidney protein N-glycome that revealed remarkable increases in disialo- and trisialo-biantennary N-glycans in *Neu1*^ΔEx3^ mice.

Notably, differences in sialylation of kidney proteins were sex specific; male *Neu1*^ΔEx3^ mice, in general, had increased amounts of N-glycans and, also, displayed specific structures, such as a biantennary disialo-glycoform bearing 1 Neu5Gc at each antenna, which were found only in traces in the tissues of female mice. Together with previous reports by Reiding et al. and Han et al. (60, 61), this is one of the few observations of sex-linked differences in glycan profiles. It will be important to explore if these sex-specific abnormalities are also recapitulated in human patients (62).

Importantly, we found higher average NEU1 activity levels in the kidneys of WT male as compared with female mice (~5 nmol/h mg versus ~3 nmol/h mg), while the NEU1 levels in other tissues were similar in mice of both sexes. These data, together with the increased levels of sialoglycans in NEU1-deficient male mice compared with females, allow us to speculate that males are more dependent on NEU1 action to ensure a proper glycosylation of kidney proteins. Thus, in a situation when NEU1 is completely or partially depleted, *Neu1*^ΔEx3^ as well as *Neu1*^Cx3cr1*Δ*Ex3^ male mice show drastic changes in glycosylation associated with (and perhaps causing) their reduced survival compared with females. While no normal comparison of sialidosis severity between male and female patients has ever been conducted, a recent study reported faster disease progression in a male compared with a female sibling (63).

Megalin is hypersialylated in *Neu1*-null mice. This was verified by both lectin blotting and immunohistochemistry coupled with high-resolution fluorescence confocal microscopy. These studies demonstrated that in kidney of *Neu1*^ΔEx3^ mice, megalin is intensely stained with SNA and not as avidly bound by PNA as in WT. Moreover, both methods revealed that levels of megalin were drastically reduced in *Neu1*-null mice and, instead of being localized to the apical membranes of PCTs, the protein was trapped in enlarged lysosomes. Megalin was also detected in the urine of *Neu1*^ΔEx3^ mice, indicating its shedding from apical membranes. Although further studies are required to understand the causal relation between megalin hypersialylation and its impaired trafficking, it is tempting to speculate that megalin is one of the proteins targeted to apical membranes via galactose-specific lectin in a carbohydrate-dependent manner (for example, via binding to galectin-3) (64). In this case, the presence of sialic acid residues would mask interactions of O-linked galactose residues with galectins, resulting in megalin relocation to lysosomes. Aberrant glycosylation of megalin occurs in the kidney of mice deficient in *Galnt11*, which encodes a member of the large glycosyltransferase family responsible for initiating mucin-type O-glycosylation of secreted and membrane-bound proteins (40). This results in age-related progressive loss of megalin from proximal renal tubules and impairment of reabsorption of LMWPs such as α1-microglobulin, retinol-binding protein, and DBP (40). In our study, substrates of megalin, β2-MG, and DBP were present only in the urine of *Neu1*-null mice, demonstrating that reduced levels of megalin or its hypersialylation is also associated with reabsorption defects. Hypoproteinemia, which could be indicative of poor recovery of urinary proteins, has been previously reported for the *Neu1*-KO mice generated by another group (26). Interestingly, the same group also reported a similar phenotype for a galactosialidosis model (*Ctsa*/*PPCA*-null mouse) with a secondary deficiency of NEU1 (65). In our galactosialidosis model, *CathA^S190A-Neo^* mice, the kidneys are normal (66), suggesting that just 10% of the residual NEU1 activity is sufficient to protect mice from a severe kidney damage.

Since the megalin-cubilin endocytic system plays a modulating role in vitamin D metabolism, it was plausible to propose that deficiency of this complex and increased urinary secretion of DBP could contribute to bone dysplasia through lower vitamin D levels. Indeed, *Neu1*^ΔEx3^ mice demonstrated a bone phenotype that may potentially be explained by a bone mineralization defect or bone formation and resorption anomalies. 25-OH vitamin D levels were significantly increased (*P* = 0.0487, Figure 7G) in the urine of *Neu1*^ΔEx3^ mice, suggesting that they were losing it through urinary secretion. At the same time, vitamin D is not deficient in plasma. Thus, further studies involving complete bone histomorphometry analyses with dynamic parameters (bone formation and resorption) are required to address this question.

Together, our results identify NEU1 as an important regulator of glycosylation of kidney proteins, including megalin, and, thus, a key element of the reabsorption process. It is tempting to speculate that desialylation of O-linked glycans may be important for transport and function of other critical proximal tubule receptors and solute carriers found to be deficient in the kidney of *Neu1*-KO mice, including the sodium-glucose cotransporter SGLT2 (51), which has important clinical relevance (67). Our findings also yield insights into the pathophysiology of nephrosialidosis and describe a model of kidney disease that implicates both glomerular and tubular defects.

## Methods

The constitutive KO NEU1 mouse model (*Neu1*^ΔEx3^) was previously described (30). *Neu1*^ΔEx3^ homozygous mice were compared with appropriate age- and sex-matched WT control littermates. To generate *Neu1*^Cx3cr1*Δ*Ex3^ strain, a mononuclear phagocyte system-specific *Neu1*-KO model, the previously reported *Neu1^ENSMUSE141558^* strain (30), was interbred with the *B6.Cg-Tg(Pgk1-flpo)10Sykr/J* line (The Jackson Laboratory, stock 011065). This cross resulted in the removal of FRT-flanked *LacZ/BactPNeo* cassette and normal expression of the *Neu1* gene in the *Neu1^loxPEx3^* strain. The *Neu1*^Cx3cr1*Δ*Ex3^ strain was obtained by crossing *Neu1^loxPEx3^* strain with the B6J.B6N(Cg)-*Cx3cr1^tm1.1(cre)Jung^*/J strain (The Jackson Laboratory, stock 025524), expressing the Cre recombinase under the control of the *Cx3cr1* gene promoter. *Neu1*^Cx3cr1*Δ*Ex3^ homozygous mice were compared with appropriate age- and sex-matched *Neu1^loxPEx3^* littermates. All mice were housed under 12-hour light/12-hour dark cycles with ad libitum access to a normal rodent chow and water. NEU1-deficient mice were euthanized on a humane basis due to urinary retention, following the advice of a veterinarian who was examining mice daily for the signs of a distorted bladder and inability to urinate. Lysosomal enzymes were assayed using corresponding fluorogenic 4-MU substrates as previously described (31, 68). Relative expression of *Neu1*, *Neu2*, *Neu3*, and *Neu4* in kidneys was determined by quantitative RT-PCR using previously described primers (30). Urine was collected in metabolic cages and analyzed with a urine dipstick, SDS-PAGE, or immunoblot. Analysis of kidney protein N-glycosylation by MALDI-TOF MS and glycosylation by lectin blotting was conducted essentially as described with or without PNGaseF treatment (37, 69). For the analysis of kidney tissues and bones by histochemistry, lectin histochemistry, immunohistochemistry, and TEM, mice were anesthetized with sodium pentobarbital and fixed by intracardiac perfusion with 4% paraformaldehyde (histochemistry) or 2% glutaraldehyde (TEM), and their tissues processed essentially as described (69, 70). Semiquantitative analysis of kidney proteins by LC-MS/MS was performed as described (71). The data were visualized with Scaffold 5.2.2, with protein threshold set at 1% FDR with a minimum of 2 peptides identified at FDR of 0.1%. Bone analysis by micro-CT was performed as described previously (72).

For complete methods, see Supplemental Methods.

### Study approval.

All animal experiments were approved by the CHU Sainte-Justine Research Ethics Committee and performed in compliance with the Comité Institutionnel des Bonnes Pratiques Animales en Recherche (approval numbers 2020-2658 and 2022-3452), in accordance with the Canadian Council on Animal Care guidelines.

### Data availability.

All data generated or analyzed during this study are included in this article and its supplemental files. Values for all data points shown in graphs and values behind any reported means are provided in the Supporting Data Values file available in the supplemental materials.

The MS data have been deposited to the ProteomeXchange Consortium via the PRIDE (73) partner repository with the data set identifier PXD044833.

## Author contributions

IK, EPD, XP, LS, AP, AM, EB, RVU, and IL conducted experiments and acquired data; IK, EPD, XP, LS, AP, CRM, DG, ML, EB, AM, IL, and AVP analyzed data; CWC provided reagents; IK, LS, and AVP wrote the manuscript (first draft); and AVP, DG, LS, ML, CWC, PT, CRM, FMW, and RVU wrote and edited the manuscript. All authors read and approved the final manuscript.

## Supplementary Material

Supplemental data

Supplemental video 1

Supplemental video 2

Supplemental video 3

Supplemental video 4

Supporting data values

## Figures and Tables

**Table 1:**
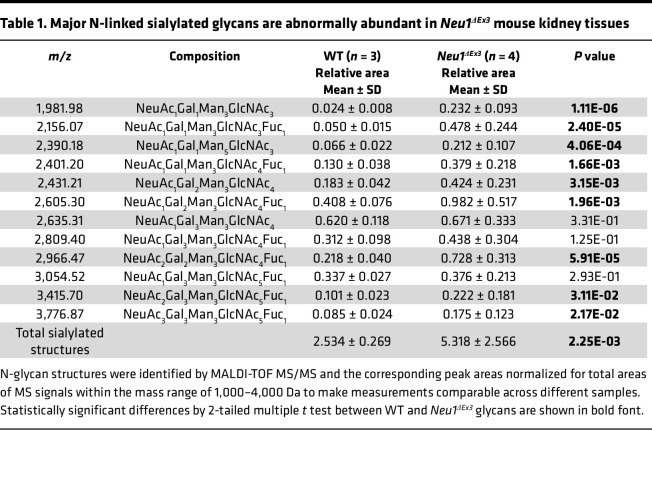
Major N-linked sialylated glycans are abnormally abundant in *Neu1^ΔEx3^* mouse kidney tissues

## Version 1 09/12/2023

In-Press Preview

## Version 2 10/23/2023

Electronic publication
