# Supplementary material for: Severe kidney dysfunction in sialidosis mice reveals an essential role for neuraminidase 1 in reabsorption
Source: JCI Insight. 2023 Oct 23;8(20):e166470. doi: 10.1172/jci.insight.166470 (PMC10619504; doi:10.1172/jci.insight.166470)
Supplement: Supplemental data [file jciinsight-8-166470-s085.pdf]

Supplemental Materials and Methods

Study approval

All animal experiments were approved by the CHU Sainte-Justine Research Ethics Committee and performed in compliance with the Comité Institutionnel des Bonnes Pratiques Animales en Recherche (CIBPAR; approval numbers 2020-2658 and 2022-3452), in accordance with the Canadian Council on Animal Care guidelines.

Animals

The constitutive KO NEU1 mouse model (*Neu1*$^{\Delta Ex3}$) was previously described [30]. Heterozygous mice were interbred, and litters were genotyped by PCR using genomic DNA extracted from clipped tail tip, as described [30]. *Neu1*$^{\Delta Ex3}$ homozygous mice were compared with appropriate age- and sex-matched WT control littermates.

To generate *Neu1*$^{Cx3cr1\Delta Ex3}$ strain, a mononuclear phagocyte system-specific *Neu1* KO model, previously reported *Neu1*^{ENSMUSE141558} strain [30], was interbred with the *B6.Cg-Tg(Pgk1-flpo)10Sykr/J* line (The Jackson Laboratory, stock 011065), that expresses the mouse codon-optimized FLP recombinase under the direction of the mouse *Pgk1*, phosphoglycerate kinase 1 promoter. This cross resulted in the removal of FRT-flanked *LacZ/BactPNeo* cassette and normal expression of the *Neu1* gene in the *Neu1*^{loxPEx3} strain (Supplementary figure S1A). The *Neu1*^{loxPEx3} strain was further crossed with the B6J.B6N(Cg)-Cx3cr1tm1.1(cre)Jung/J strain (The Jackson Laboratory, stock 025524), expressing the Cre recombinase under the control of the *Cx3cr1* (chemokine C-X3-C motif receptor 1) gene promoter. In the *Cx3cr1*-expressing cells of the progeny of *Neu1*^{loxPEx3} and B6J.B6N(Cg)-Cx3cr1tm1.1(cre)Jung/J, the exon 3 of the *Neu1* gene was consequently removed, as confirmed by Sanger sequencing. Heterozygous *Neu1*$^{Cx3cr1\Delta Ex3}$ mice were bred to each other, and litters genotyped by PCR using genomic DNA

extracted from clipped tail tip as shown in the Supplementary figure S1B. *Neu1*$^{Cx3cr1\Delta Ex3}$ homozygous mice were compared with appropriate age- and sex-matched *Neu1*^{loxPEx3} littermates. All mice were housed in an enriched environment in a poly-carbonate cages under 12 h/12 h light - dark cycles in a temperature- and humidity-controlled room. Mice had *ad libitum* access to a normal rodent chow and water.

Analysis of mouse phenotype

Neu1$^{\Delta Ex3}$ and *Neu1*$^{Cx3cr1\Delta Ex3}$ mice were weighted weekly, from 4 to 17 weeks of age. Survival was measured for 21 male *Neu1*$^{\Delta Ex3}$ and 18 female *Neu1*$^{\Delta Ex3}$ mice siblings, 39 male and 38 female *Neu1*$^{Cx3cr1\Delta Ex3}$ siblings, and for the matching numbers of male and female WT controls. NEU1-deficient mice were euthanized on a humane basis due to urinary retention following the advice of a veterinarian who was examining mice daily for the signs of a distorted bladder and inability to urinate. After euthanasia, the visceral organs were dissected, and the wet weight of the kidney, liver and spleen was measured and recorded.

Lysosomal enzyme assays

Total acidic α-neuraminidase, β-galactosidase and β-hexosaminidase activities were measured in tissue homogenates using corresponding fluorogenic 4-methylumbelliferyl substrates as previously described [68]. NEU1 activity was measured by supplementing the α-neuraminidase assay mixture with the specific inhibitor for both NEU3 and NEU4 enzymes, CG17700 (also known as C9-4BPT-DANA), at a final concentration of 125 μM as previously described [31]. Protein concentration was measured using Pierce BCA protein assay kit (#23225, Thermo Scientific).

Quantitative RT-PCR

Whole kidneys were collected, and RNA extracted using Trizol reagent (Invitrogen) according to manufacturer's protocol. RNA quality was assessed by Nanodrop (Thermo Scientific) and 1 µg of RNA was subjected to reverse transcription with QuantiTect Reverse Trasncription kit (Qiagen). Analyses were performed in duplicate for each sample using SYBR Green Supermix (Bio-Rad) and previously described primers [30]. Relative expression of *Neu1*, *Neu2*, *Neu3* and *Neu4* in kidneys were determined by the $2^{-\Delta\Delta CT}$ method and normalized to the levels of *RPL32* mRNA.

Analysis of urinary proteins

Urine was collected by housing mice in metabolic cages for 6 h while mice had free access to water. Mouse urine was, first, analyzed with a urine dipstick (Siemens, Multistix 10 SG). Then, urinary proteins were precipitated using trichloroacetic acid (TCA), as previously described [69], with some modifications. Briefly, TCA was added to 50 µL of urine at a final concentration of 4%. Samples were mixed by vortexing, incubated on ice for 20 min and centrifuged at 13,000 g for 15 min. Pellets were rinsed with acetone and dried at room temperature. Protein pellets were dissolved in 40 µL of 4x Laemmli buffer (#BP-110R, Boston Bioproducts), and pH was adjusted with 5 µL of 1 M Tris buffer, pH 8.4. Proteins were denatured by boiling for 5 min, resolved by SDS-PAGE on 8% acrylamide gel and stained with freshly prepared Coomassie brilliant blue for 1 h. Gels were de-stained with 10% acetic acid under gentle agitation overnight at a room temperature. For immunoblots, urine protein samples were resolved on NuPAGE 3-8% Tris-Acetate gradient gels (Invitrogen, EA03785BOX),

transferred to nitrocellulose membranes, blocked with 5% bovine serum albumin (BSA) in phosphate buffered saline (PBS) and hybridized with the following antibodies:

Antigen	Host/Target species	Dilution	Manufacturer, catalogue number
Megalin	Rabbit anti-human	1:1000	Abcam, Ab76969
Vitamin D binding protein (DBP)	Mouse monoclonal	1:500	Santa Cruz, A-5
β_2-microglobulin	Mouse monoclonal	1:1000	Santa Cruz, BBM.1

Blood glucose measurement

Animals were kept in cages under fasting conditions for 16 h, with free access to water. Blood, collected from clipped tail tip, was analyzed using a glucometer (Contour Next, Bayer).

Analysis of kidney protein glycosylation by lectin blotting

Whole kidneys were homogenized with 250 µL of RIPA buffer (50 mM Tris HCl, 150 mM NaCl, 1% Nonidet P-40, 0.25% Na-deoxycholate, 0.1% SDS, 2 mM EDTA, 1 mM PMSF, pH 7.5), supplemented with protease and phosphatase inhibitor cocktail (Roche), using a sonic dismembrator (Artek Systems Corporation). The homogenate was cleared by centrifugation at 13,000 *g* for 25 min, and the protein concentration was measured with Pierce BCA protein assay kit (#23225, Thermo Scientific) and adjusted to 0.8 µg/µL. Samples were boiled for 5 min and separated by SDS-PAGE on an 8% gel. Blots were transferred to nitrocellulose membrane. After staining with a Pierce™ Reversible Protein Stain Kit for Nitrocellulose Membranes (ThermoFisher Scientific) and blocking with 5% BSA for 1 h at room temperature, membranes were incubated overnight at 4°C with biotinylated peanut agglutinin (1:1000, PNA, Vector

Laboratories, Burlington, ON, Canada) or biotinylated *Sambucus nigra* lectin (1:5000, SNA, Vector Laboratories, Burlington, ON, Canada) and, then, exposed to streptavidin-horseradish peroxidase conjugate (1:2000; GE Healthcare Life Sciences, Baie-d'Urfé, QC, Canada). Lectin reactivity was revealed by enhanced chemiluminescence (ECL) using the Pierce™ ECL Western Blotting Substrate (Thermo Fisher Scientific Inc., Rockford, USA). The total intensities of the stained protein bands were quantified by ImageJ software (Rasband, W.S., ImageJ, U.S. National Institutes of Health, Bethesda, Maryland, USA, https://imagej.net/) and normalized for the combined intensity of protein bands stained with Pierce™ Reversible Protein Stain.

Whole kidneys were homogenized in 0.1% Rapigest (Waters Corporation) dissolved in freshly prepared 50 mM ammonium bicarbonate containing 5 mM DTT. Protein concentration was measured with Pierce BCA protein assay kit (#23225, Thermo Scientific), adjusted to 4.1 µg/µL, and resolved on gradient NuPAGE 3-8% Tris-Acetate gels (Invitrogen, EA03785BOX). Proteins were transferred to nitrocellulose membrane, blocked, and stained with antibodies and lectins as listed below.

Antigen	Host/Target species	Dilution	Manufacturer, catalogue number
Megalin	Rabbit anti-human	1:1000 in 3% milk in TBS, pH 8.4	Abcam, Ab76969
Peanut agglutinin (PNA)	Biotinylated	1:2000	Vectorlabs, B-1075-5
Maackia Amurensis Lectin II (MALII)	Biotinylated	1:1000	Vectorlabs, B-1265-1
Ricinus Commuis Agglutinin I (RCA-1)	Biotinylated	1:1000	Vectorlabs, RCA120

Sambucus Nigra Lectin (SNA)	Biotinylated	1:5000	Vectorlab, B-1305-2

To analyse *N*-linked protein glycans, kidneys were homogenized in 0.1% Rapigest prepared in 50 mM ammonium bicarbonate. The homogenate was cleared by centrifugation at 13000 g for 20 min, supplemented with DTT to a final concentration of 5 mM, and incubated at 60°C for 30 minutes. A 20 µg aliquot of each sample treated or not with PNGaseF (New England Biolabs, P0704L) to remove *N*-linked glycans [70], or *Arthrobacter ureafaciens* bacterial sialidase (Roche #10269611001) to remove sialylations, were resolved on 3-8% gradient NuPAGE Tris-Acetate gel (Invitrogen, EA03785BOX), and transferred to nitrocellulose membrane. The membrane was blocked with 3% milk in 0.05% TBS-Tween 20 for 1 h at a room temperature and hybridized for 2 h at a room temperature with streptavidin (1:5000) or anti-rabbit (1:5000) HPR-conjugated secondary antibody. Signals were revealed using Pierce™ ECL Western Blotting Substrate (Thermo Fisher Scientific Inc., Rockford, USA).

The following antibodies were used:

Antigen	Host/Target species	Dilution	Manufacturer, catalogue number
Megalin	Rabbit anti-human	1:1000 in 3% milk in TBS, pH 8.4	Abcam, Ab76969
Peanut agglutinin (PNA)	Biotinylated	1:2000	Vectorlabs, B-1075-5
Sambucus Nigra Lectin (SNA)	Biotinylated	1:5000	Vectorlab, B-1305-2

Ricinus Communis Agglutinin I (RCA-1)	Biotinylated	1:1000	Vectorlabs, RCA120

Analysis of kidney protein N-glycosylation by MALDI MS

Mouse kidney tissue was homogenized and lysed in a chloroform/methanol/water mixture 4:8:3 (v/v/v) as described previously [71]. The protein pellet was separated from the lipid-containing supernatant by centrifugation, cleaned by repeated washes with acetone/water (4:1) at 4°C, dried under a stream of nitrogen and stored at -20°C. An additional cleaning step was performed during the analysis, by resuspending the protein pellet in ddH$_2$O, followed by centrifugation. The supernatant, containing free urinary oligosaccharides accumulating as undegraded NEU1 substrates [36], was concentrated and analyzed separately by permethylation and MALDI MS. To accomplish the *N*-linked glycan analysis, the washed protein pellet (~2 mg) was resuspended in 0.2 mL of 0.1% RapiGest^{TM} Surfactant (Waters Corporation, Milford, Massachusetts) in 50 mM NH_4HCO_3 using an ultrasonic processor equipped with a 2 mm probe (130 Watt, 50% amplitude, 5 min in pulsing mode). The obtained homogenate was incubated at 100 °C for 5 min, followed by reduction in 5 mM dithiothreitol (DTT, Sigma) at 56°C for 30 min and alkylation in 15 mM iodoacetamide (IAA, Sigma) in the dark, at room temperature for 45 min. Glycan chains were cleaved by peptide-N-glycosidase F (PNGase F) (4U, Roche Molecular Biochemicals, Mannheim, Germany) overnight at 37 °C. The released N-glycans were purified, and permethylated by ICH_3 in a DMSO/NaOH slurry as described [72, 73]. MALDI TOF and MALDI TOF/TOF analyses of permethylated N-glycans were performed using 5-chloro-2-mercaptobenzothiazole (CMBT, 10 mg/ml in 80:20 methanol/water, v/v) as a matrix, and acquired on a 4800 proteomic Analyzer (AB Sciex) in positive polarity and in reflector mode [72]. Data were analyzed using DataExplorer^{TM} 4.9 software. Glycan structures were assigned

based on molecular weight, knowledge of the biosynthetic pathway and MS/MS analyses, using the bioinformatic tools developed by the Consortium for Functional Glycomics (http://functionalglycomics.org).

Analysis of kidney protein N-glycosylation by HILIC-UPLC-FLR-ESI-MS

Analysis of protein N-glycosylation by HILIC-UPLC-FLR-ESI-MS was performed as previously described [37]. Briefly, enzymatic release and labelling of *N*-glycans was performed using the GlycoWorks™ RapiFluor-MS™ *N*-Glycan kit (Waters Corporation, Milford, MA, USA), per manufacturer's protocol with the exception of deglycosylation reaction time which was extended from 5 to 20 min. *N*-glycans, labeled at the glycosylamine residue of the terminal chitobiose epitope, were separated by an UHPLC Thermo system (Vanquish™ Flex System VF-501-A-02) coupled to an Orbitrap Exploris™ 120 HESI EASY-IC mass spectrometer (Thermo Fisher Scientific Inc., Bremen, Germany) equipped with a Vanquish™ fluorescence detector (VC-D50-A). Samples were separated using a Hydrophilic Interaction Liquid Chromatography (HILIC) column (ACQUITY UPLC Glycan BEH Amide 130 Å, 1.7 µm, 2.1 × 150 mm, Waters Corporation Milford, MA, USA) at a flow rate of 0.4 ml/min at 60 °C, with 200 mM ammonium formate aqueous solution (pH 4.4), as a mobile phase A, and acetonitrile (ACN), as a mobile phase B. The gradient ramped from 25% A to 46 % A in 35 min.

MS analyses were conducted under the following conditions: heater temperature 275 °C, capillary temperature 250 °C, spray voltage 3.3 kV, RF lens 70 %. Spectra were acquired in positive polarity, and resolution was adjusted at 30000 FWHM at 400 m/z. Full scan spectra were acquired in the mass range of 700-2000 m/z using the Automatic Gain Control (AGC) target with Data-Dependent MS2 Analysis (DDA) by higher-energy collision dissociation (HCD) of the four most abundant precursor ions, each with normalized collision energy (NCEs) at 25,

35, and 65 %. Isolation window was set as 0.5 m/z, with Orbitrap MS2 resolution of 15000, automated scan range mode, standard AGC target, and automated maximum injection time. Fluorescence detection was conducted using wavelengths of 265 nm (excitation) and 425 nm (emission) with a sampling rate of 2 Hz. Three technical replicates were performed for each sample and three runs for each analysis with no substantial differences identified for chromatograms and spectra between samples.

Histochemistry and immunohistochemistry

Animals were deeply anaesthetized with sodium pentobarbital and fixed by intracardiac perfusion with 4% paraformaldehyde prepared in PBS, pH 7.4. Kidneys were removed and immersed in 4% paraformaldehyde in PBS overnight, changed to 70% ethanol before tissue processing. The tissues were dehydrated, cleared for 18 h in the tissue transfer processor (Leica TP1020), embedded in paraffin (Leica EG1160) and stored at 4° C. Kidney tissues were sliced to 5 μm thickness using a microtome (Leica RM2145), placed on glass slides, and dried at 37 °C overnight. For routine H&E staining, samples were first deparaffinized with xylene and rehydrated with ethanol in decreasing concentrations of 100%, 95%, 75% and 60%. Then, the slides were stained with hematoxylin (MHS16, Sigma) and Eosin (Vintage Eosin-Y, StatLab) and mounted with Eukitt Quick-hardening mounting medium (03989, Sigma). For immunostaining, after deparaffinization and rehydration, kidney sections were boiled in 10 mM sodium citrate buffer with 0.05% Tween 20, pH 6.0, for 15 min for antigen retrieval. Tissue sections were blocked for 1 h with 5% BSA and 0.04% Tween 20 at room temperature and stained with Cy3-labelled PNA (1:100, Vector Laboratories) and Cy5-labelled SNA (1:100, Vector Laboratories), diluted in carbo-free blocking solution, for 1 h at room temperature, and

mounted with DAPI-containing Prolong Gold Antifade Mounting medium (P36931, ThermoFisher). Slides were analyzed by Axioscan (Zeiss), and images were processed using the ZenBlue Lite program (Zeiss).

For fluorescent confocal microscopy analyses, PFA-fixed kidneys were transferred to a 30% sucrose in PBS, incubated overnight at 4°C, embedded in OCT (Sakura) and kept frozen at -80 °C. OCT-embedded frozen kidney blocks were cut using a cryostat (Leica) to 15 µm thickness, blocked and permeabilized with 1% Triton X-100 in 10% goat serum for 1 h at room temperature. Tissue sections were stained with the following antibodies:

Antigen	Host/Target species	Dilution	Manufacturer, catalogue number
Megalin	Rabbit anti-human	1:200 in 3% milk in PBS, pH 8.4	Abcam, Ab76969
Peanut agglutinin (PNA), Cy3-labelled		1:100	Vectorlabs, CL-1073-1
Sambucus Nigra Lectin (SNA), Cy5-labelled		1:100	Vectorlab, CL-1305-1
Ricinus Communis Agglutinin I (RCA I), Rhodamine-labelled		1:100	Vectorlabs, RL-1082-5
LAMP1	Rat anti-human	1:50	DSHB, H4A3
CD68	Rabbit	1:200	Abcam, Ab125212

Slides were mounted with DAPI-containing Prolong Gold Antifade Mounting medium (P36931, ThermoFisher) and analyzed using SP8-DLS inverted confocal microscope (Leica TCS SP8). Images were processed and quantified using ImageJ.

For nephron quantification, H&E stained 5 µm thick paraffin sections of age and sex matched *Neu1*$^{\Delta Ex3}$ (n=3), *Neu1*$^{Cx3cr1\Delta Ex3}$ (n=3) and WT (n=4) mice were scanned with Axioscan at 40x brightfield. Five 823.53 µm x 912.06 µm regions of kidney cortex were selected at the same positions, and glomeruli were manually counted and normalized (multiplied) by the wet weight of the kidney.

Transmission Electron Microscopy

Male and female WT, *Neu1*$^{\Delta Ex3}$, *Neu1*^{loxPEx3} and *Neu1*$^{Cx3cr1\Delta Ex3}$ mice were anesthetised with sodium pentobarbital and perfused with PBS and 5% glutaraldehyde in 0.1 M phosphate buffer, pH 7.0. After perfusion, organs were carefully removed and post-fixed by immersion in 5% glutaraldehyde overnight at 4 °C. Kidney samples were trimmed to obtain the cortex regions that were, then, washed with 0.1 M cacodylate buffer, pH 7.4, before secondary fixation with 1% osmium tetroxide and 1.5% potassium ferrocyanide for 2 h. Samples were dehydrated with an ethanol series and propylene oxide, infiltrated with epoxy resin, and, embedded in Durcupan®-Epon® mixture. Semi-thin (1 µm thick) sections were cut, mounted on glass slides, stained with toluidine blue and examined on Leica DMS light microscope to select the sections eventually used for electron microscopy.

The Epon blocks were trimmed, and 100 nm ultrathin sections were cut with an Ultracut E ultramicrotome. Samples were mounted on 200-mesh copper grids, stained with uranyl acetate (Electron Microscopy Sciences) and lead citrate, and examined on a FEI Tecnai G2 Spirit

BioTwin 120 kV Cryo-TEM at McGill University Facility for Electron Microscopy Research (FEMR). PCT images were taken at the 4800x magnification, DCT, at 4800x magnification, glomeruli, at 1200x magnification and podocytes, at 13000x magnification.

Liquid Chromatography-Mass Spectrometry

Samples were reconstituted in 50 mM ammonium bicarbonate with 10 mM TCEP [Tris(2-carboxyethyl) phosphine hydrochloride (Thermo Fisher Scientific], and vortexed for 1 h at 37°C. Chloroacetamide (Sigma-Aldrich) was added for alkylation to a final concentration of 55 mM. Samples were vortexed for another hour at 37°C. One microgram of trypsin was added, and digestion was performed for 8 h at 37°C. Samples were dried down and solubilized in 5% ACN-4% formic acid (FA). Peptides were loaded and separated on a home-made reversed-phase column (150-µm i.d. by 200 mm) with a 56-min gradient from 10 to 30% ACN-0.2% FA and a 600-nl/min flow rate on an Easy nLC-1000 connected to an Orbitrap Fusion (Thermo Fisher Scientific, San Jose, CA). Each full MS spectrum acquired at a resolution of 120,000 was followed by tandem-MS (MS-MS) spectra acquisition on the most abundant multiply charged precursor ions for a maximum of 3 s. Tandem-MS experiments were performed using higher energy collision dissociation (HCD) at a collision energy of 27%. The data were processed using PEAKS X (Bioinformatics Solutions, Waterloo, ON) and a Uniprot mouse database. Mass tolerances for precursor and fragment ions were 10 ppm and 0.01 Da, respectively. Fixed modification was carbamidomethyl (C). Variable selected posttranslational modifications were acetylation (N-ter), oxidation (M), deamidation (NQ), and phosphorylation (STY). The data were visualized with Scaffold 5.2.2, showing protein threshold at 1% false discovery rate (FDR) with a minimum of 2 peptides identified at FDR of 0.1%).

Bone analysis

The left tibia bone of each mouse was scanned in a Micro-CT scanner (eXplore Locus, GE Healthcare Canada) with a tube voltage of 80 kVP, current of 450 µA, 2000 ms integration time and a resolution of 20 µM. Images analysis allowed the evaluation of cortical bone parameters: cortical bone volume, bone mineral density, bone mineral content and thickness using an automatic bone analysis software (eXplore MicroView from GE Healthcare, Canada). The bone specimens were then decalcified using CAL-EX (Cal-Ex™ II Fixative/Decalcifier, Fisher Chemical™) before embedding in paraffin, slicing with a microtome (Thermo Scientific Microm HM 325-2 Manual Microtome) at 4 microns and staining with Masson Trichrome. Static bone histomorphometry measures were assessed on a single section for each animal for three regions: primary spongiosa, growth plate and secondary spongiosa for the trabecular bone parameters (Bioquant Meg IV System; R & Biometrics, TE, USA), using an Olympus BX45 microscope (Olympus, Richmond Hill, ON, Canada). A standardized ROI was used for each measure for all animals.

Analysis of 25-hydroxy vitamin D (25-OH) by ELISA

Mouse 25-hydroxy vitamin D (25-OH) was analysed in plasma (n=6 per genotype) and urine (n=2 per genotype) of $Neu1^{\Delta Ex3}$ and WT mice using an ELISA kit (MyBioSource #MBS263918) as per manufacturer's protocol. Briefly, plasma and urine samples were diluted at 1:5 and assessed for 25-OH in a double antibody sandwiched ELISA technique, and absorbance values were measured with Clariostar (BMG Labtech).

Statistical analysis

Statistical analyses were performed using Prism GraphPad 9.3.0. software (GraphPad Software San Diego, CA). The normality for all data was checked using the D'Agostino & Pearson omnibus normality test. The significance of the difference was determined using t-test when comparing two groups and one-way ANOVA test, followed by a Tukey multiple comparison test, when comparing more than two groups. Two-way ANOVA followed by Bonferroni or a Tukey post hoc tests was used for two-factor analysis. A P-value of 0.05 or less was considered significant.

Supplementary data

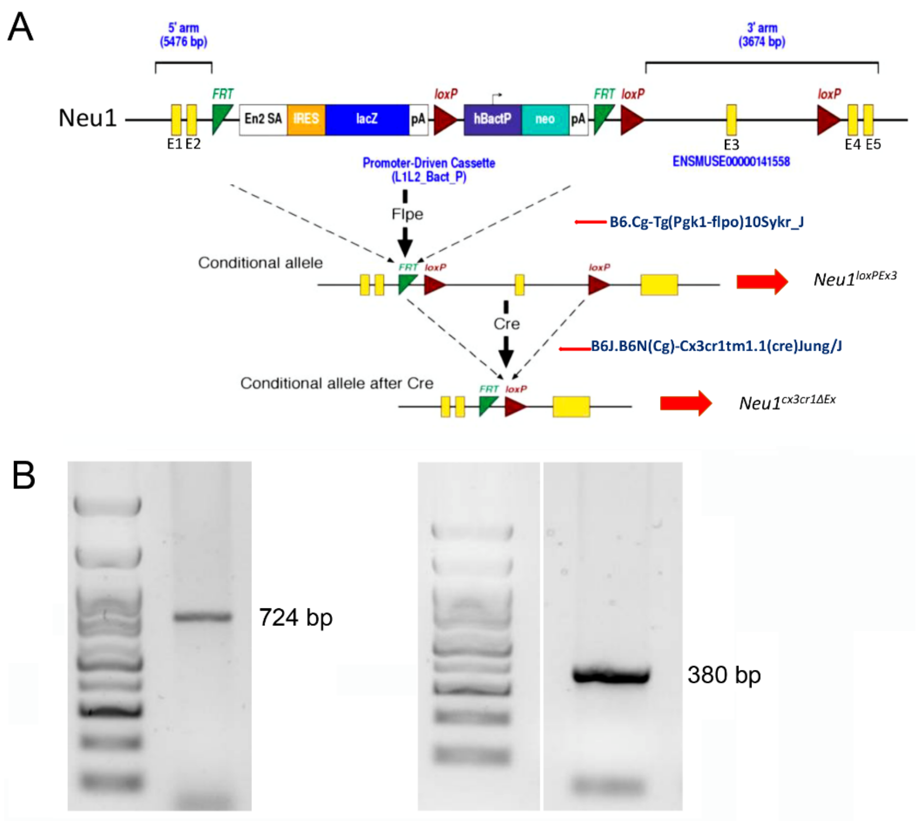

Figure S1. Generation of $Neu1^{Cx3cr1\Delta Ex3}$ mouse strain.

(A) Scheme showing generation of a conditional *Neu1* KO strain, $Neu1^{Cx3cr1\Delta Ex3}$. Previously described $Neu1^{ENSMUSE141558}$ strain was interbred with the *B6.Cg-Tg(Pgk1-flpo)10Sykr/J* line (The Jackson Laboratory stock 011065), that expresses the mouse codon-optimized FLP recombinase under the direction of the mouse *Pgk1*, phosphoglycerate kinase 1 promoter. This caused the removal of FRT-flanked *Neo* cassette allowing normal expression of the *Neu1* gene in the resulting $Neu1^{loxPEx3}$ strain. The $Neu1^{loxPEx3}$ strain was then crossed with the B6J.B6N(Cg)-Cx3cr1tm1.1(cre)Jung/J strain (The Jackson Laboratory stock 025524) expressing the Cre recombinase under the control of the *Cx3cr1* (chemokine C-X3-C motif receptor 1) gene promoter. **(B)** $Neu1^{Cx3cr1\Delta Ex3}$ mice were genotyped by PCR of tail genomic DNA for the presence of $Neu1^{loxPEx3}$ (724 bp PCR fragment) and *Cx3cr1Cre* (380 bp fragment).

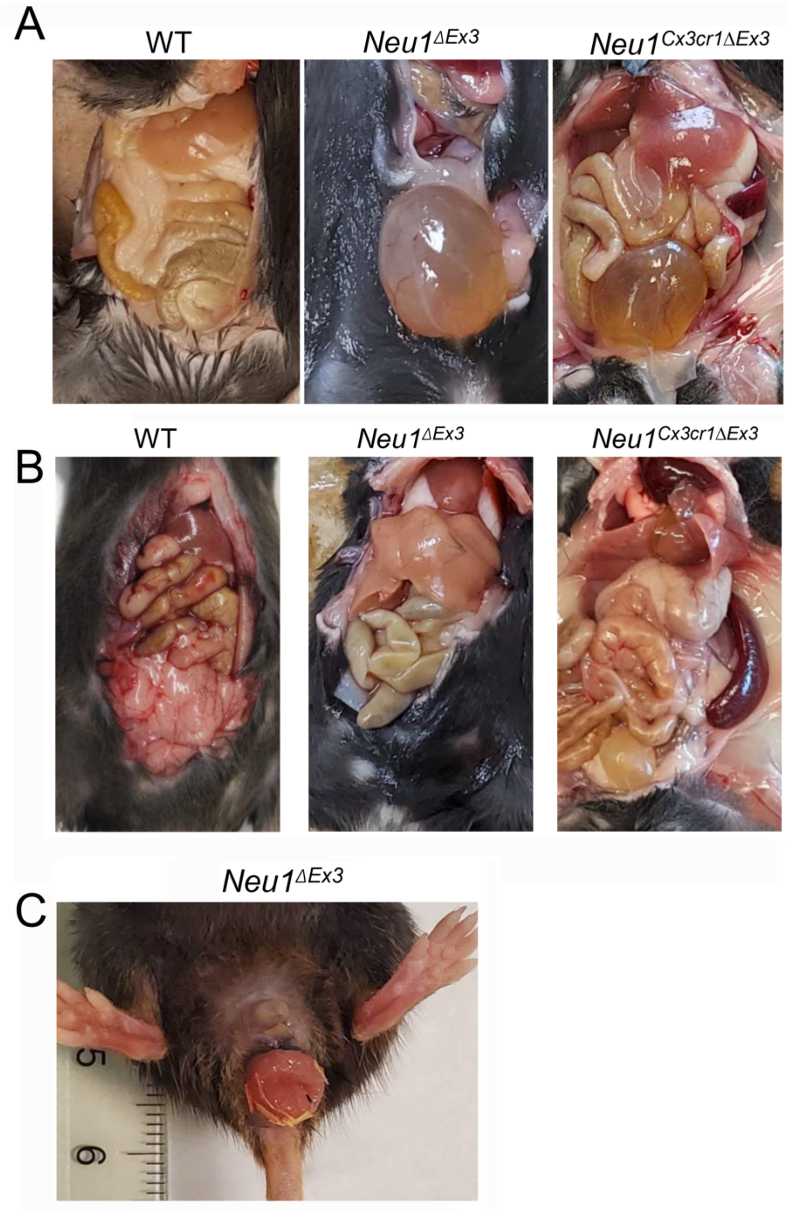

Figure S2. Necropsy of *Neu1^{ΔEx3}* and *Neu1^{cx3cr1ΔEx3}* mice at the terminal stage

(A) *Neu1^{ΔEx3}* and *Neu1^{cx3cr1ΔEx3}* mice at the terminal stage show an inability to urinate causing distention of bladder, not present in the WT mouse. Pictures show representative necropsy images of male 4-month-old *Neu1^{ΔEx3}* mouse, 7-month-old *Neu1^{cx3cr1ΔEx3}* mouse and 7-month-old WT mouse. **(B)** Four-month-old female *Neu1^{ΔEx3}* and *Neu1^{Cx3cr1ΔEx3}* mice show reduced abdominal fat when compared to WT mouse, accompanied by visibly enlarged liver in *Neu1^{ΔEx3}* and enlarged spleen in *Neu1^{Cx3cr1ΔEx3}* mouse. **(C)** Severe rectal prolapse in 6.5-month-old *Neu1^{ΔEx3}* female mouse.

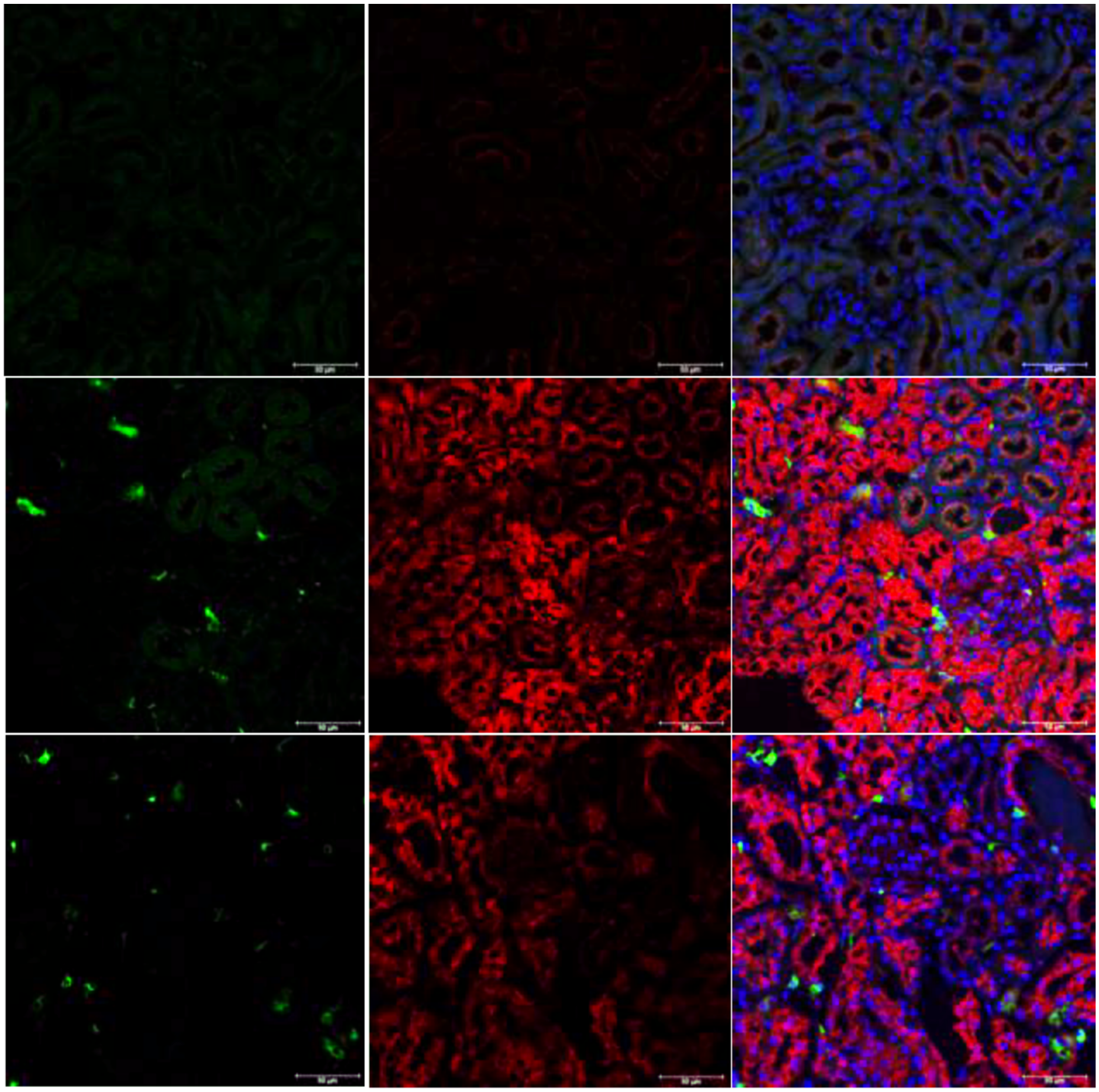

Figure S3. Infiltration of CD68+ activated macrophages and increased lysosomal biogenesis (LAMP1+ cells) in kidney of 4-month-old *Neu1$^{\Delta Ex3}$* and *Neu1$^{Cx3cr1\Delta Ex3}$* mice.

Panels show representative confocal microscopy images of kidney tissues of 4-month-old *Neu1$^{\Delta Ex3}$* and *Neu1$^{Cx3cr1\Delta Ex3}$* mice and their age-matching WT control stained with antibodies against CD68 (green, activated macrophages) and LAMP1 (red, lysosomes). DAPI (blue) was used as a nuclear counterstain.
Graphs show quantification of fluorescence with ImageJ software. Individual data, means and SD obtained for 2 mice per genotypes (3 areas/mouse) are shown. P values were calculated using Nested one-way ANOVA test with a Tukey post hoc test.

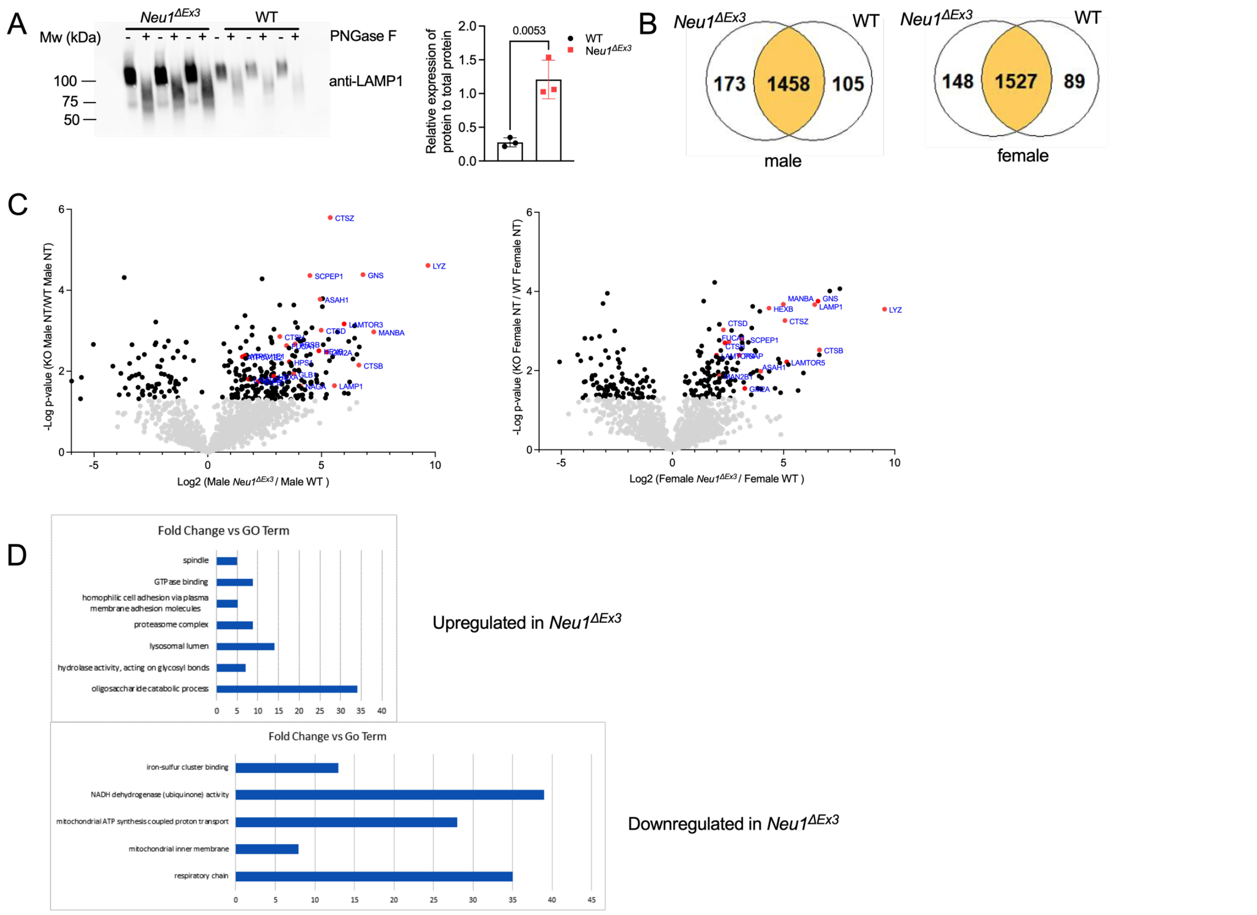

Figure S4. Semiquantitative LC-MS/MS analysis of kidney proteins reveals increased levels of lysosomal proteins in male and female 4-month-old *Neu1$^{\Delta Ex3}$* mice.

(A) Immunoblot analysis of kidney protein extracts treated or not with PNGaseF demonstrates increased intensity of LAMP-1 immunoreactive band in kidney of *Neu1$^{\Delta Ex3}$* mice but a similar shif of the LAMP-1 band position after PNGaseF treatment. Graph shows quantification of the band intensity with ImageJ softare. Individual results, means and SD are shown (n=3). P values were calculated by one-way ANOVA with Dunn's post hoc test. **(B)** Total number of proteins identified in kidney of male and female *Neu1$^{\Delta Ex3}$* and WT mice. **(C)** Volcano plots of kidney proteins showing differentially regulated proteins that are statistically different in male and female *Neu1$^{\Delta Ex3}$* and WT mice. Representative lysosomal proteins are shown as red dots and annotated. **(D)** Gene ontology (GO) terms plotted versus fold chages for protein groups downregulated or upregulated in *Neu1$^{\Delta Ex3}$* mice in a sex-specific manner.

A

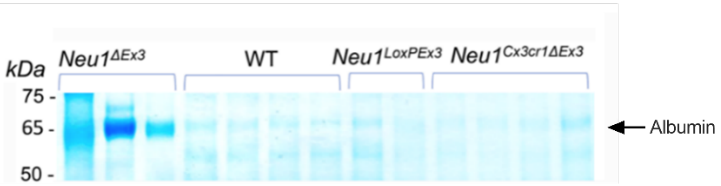

B

Genotype	Protein (g/L)	pH	Glucose	Specific gravity
WT (n=5)	Negative	6.5	Negative	1.030
Neu1$^{\Delta Ex3}$ (n =5)	0.3	5.0	5.5	1.030
Neu1$^{Cx3cr1\Delta Ex3}$ (n=4)	Trace	5.0	Negative	1.030

C

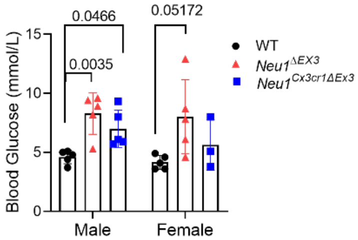

D

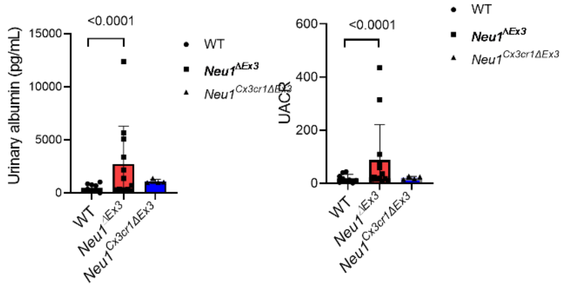

Figure S5. Four-month-old *Neu1*$^{\Delta Ex3}$ mice develop proteinuria.

(A) SDS-PAGE analysis of urinary proteins showing a presence of a 65 kDa albumin protein band (arrow) in the urine of 4-month-old *Neu1*$^{\Delta Ex3}$ mice but not of WT or *Neu1*$^{Cx3cr1\Delta Ex3}$ mice. Image shows a representative SDS-PAGE analysis of urine from 3 *Neu1*$^{\Delta Ex3}$, 4 WT, 2 *Neu1*^{LoxPEx3} with normal Neu1 expression and 4 *Neu1*$^{Cx3cr1\Delta Ex3}$ age-matched mice. **(B)** Urine dipstick (Siemens, Multistix 10 SG) test results showing presence of protein, reduced pH, normal specific gravity, and increased glucose levels in the urine of 4-month-old *Neu1*$^{\Delta Ex3}$ mice. (**C**) Elevated blood glucose levels were detected in fasted male and female *Neu1*$^{\Delta Ex3}$ mice, and in male *Neu1*$^{Cx3cr1\Delta Ex3}$ mice. **(D)** ELISA analysis showing reduction of urinary creatinine and a trend for the increase of urinary albumin and urine albumin-to-creatinine ratio (UACR) in 4-month-old *Neu1*$^{\Delta Ex3}$ mice consistent with kidney disfunction. Plasma and urine were collected from 4-month-old and 6-month-old WT (n=9), *Neu1*$^{\Delta Ex3}$ (n=13) and *Neu1*$^{Cx3cr1\Delta Ex3}$ (n=4) mice of both sexes. Levels of plasma and urinary creatinine were quantified by Creatinine kit (Chondrex Inc), and urinary albumin was quantified by ELISA (Abcam).

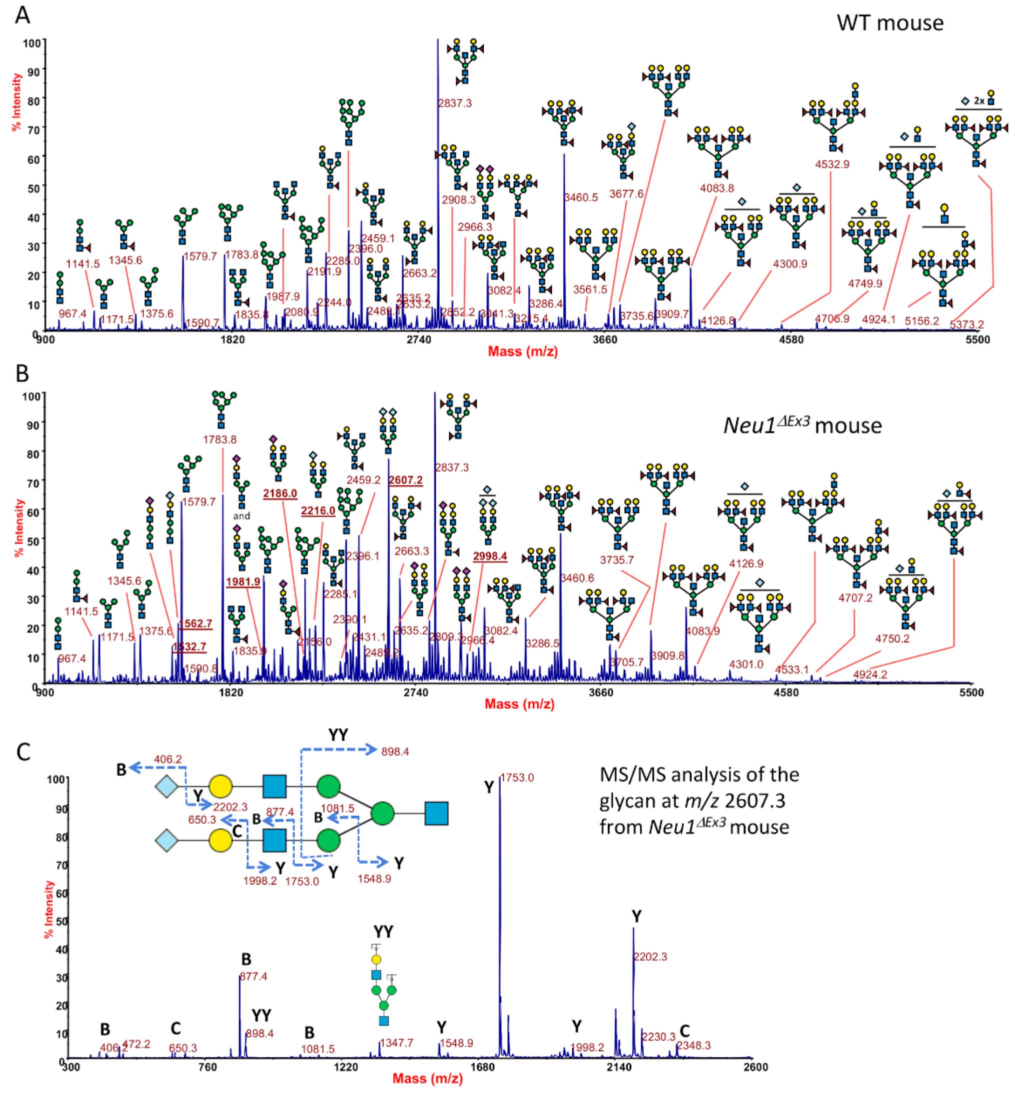

Figure S6. MALDI mass spectrometry analysis shows the presense of free glycans stored in kidney tissues of 4-month-old *Neu1$^{\Delta Ex3}$* mice.

Representative spectra of N-glycans from kidney tissue of **(A)** WT and **(B)** *Neu1$^{\Delta Ex3}$* mouse. A number of sialylated glycans at *m/z* 1532.7, 1562.7, 1981.9, 2186.0, 2216.0, 2607.2, and 2998.4 (underlined) are present only in *Neu1$^{\Delta Ex3}$* profiles, and most likely represent free oligosaccharides stored in the lysosomes of kidney tissues and secreted in the urine of NEU1 KO mice. **(C)** MS/MS spectrum of the most abundant species at *m/z* 2607.2, shows the presence of a GlcNAc at the reducing glycan end, instead of the disaccharide GlcNAc-GlcNAc, which is expected to be found in an N-linked glycan released from a glycoprotein or a glycopeptide after digestion with PNGase F. GlcNAc, blue square; Man, green circle; Gal, yellow circle; Neu5Ac, purple diamond; Neu5Gc, light blue diamond; Fuc, red triangle.

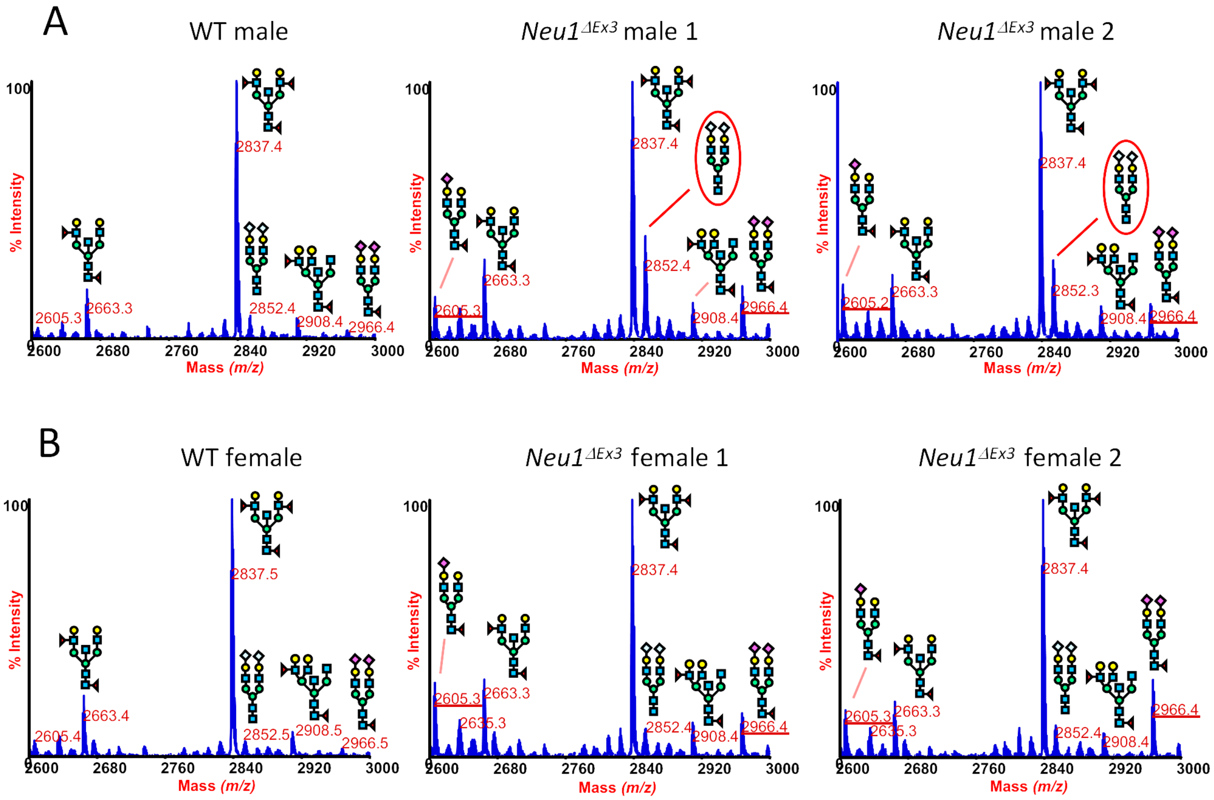

Figure S7. MALDI MS spectra of N-linked glycans from kidney tissue are sex-specific in *Neu1^{ΔEx3}* mice.

Partial (mass-range between *m/z* 2600 and 3000) MALDI MS profiles from a 4-month-old male WT mouse and its age-matching male **(A)** and female **(B)** *Neu1^{ΔEx3}* littermates. Both male and female *Neu1^{ΔEx3}* mice show increase in sialylated structures (underlined *m/z* values) as compared to WT. Additionally, male *Neu1^{ΔEx3}* show an accumulation of the glycoform at *m/z* 2852.4 (circled in red) not occurring in female *Neu1^{ΔEx3}* mice. GlcNAc, blue square; Man, green circle; Gal, yellow circle; Neu5Ac, purple diamond; Neu5Gc, light blue diamond; Fuc, red triangle.

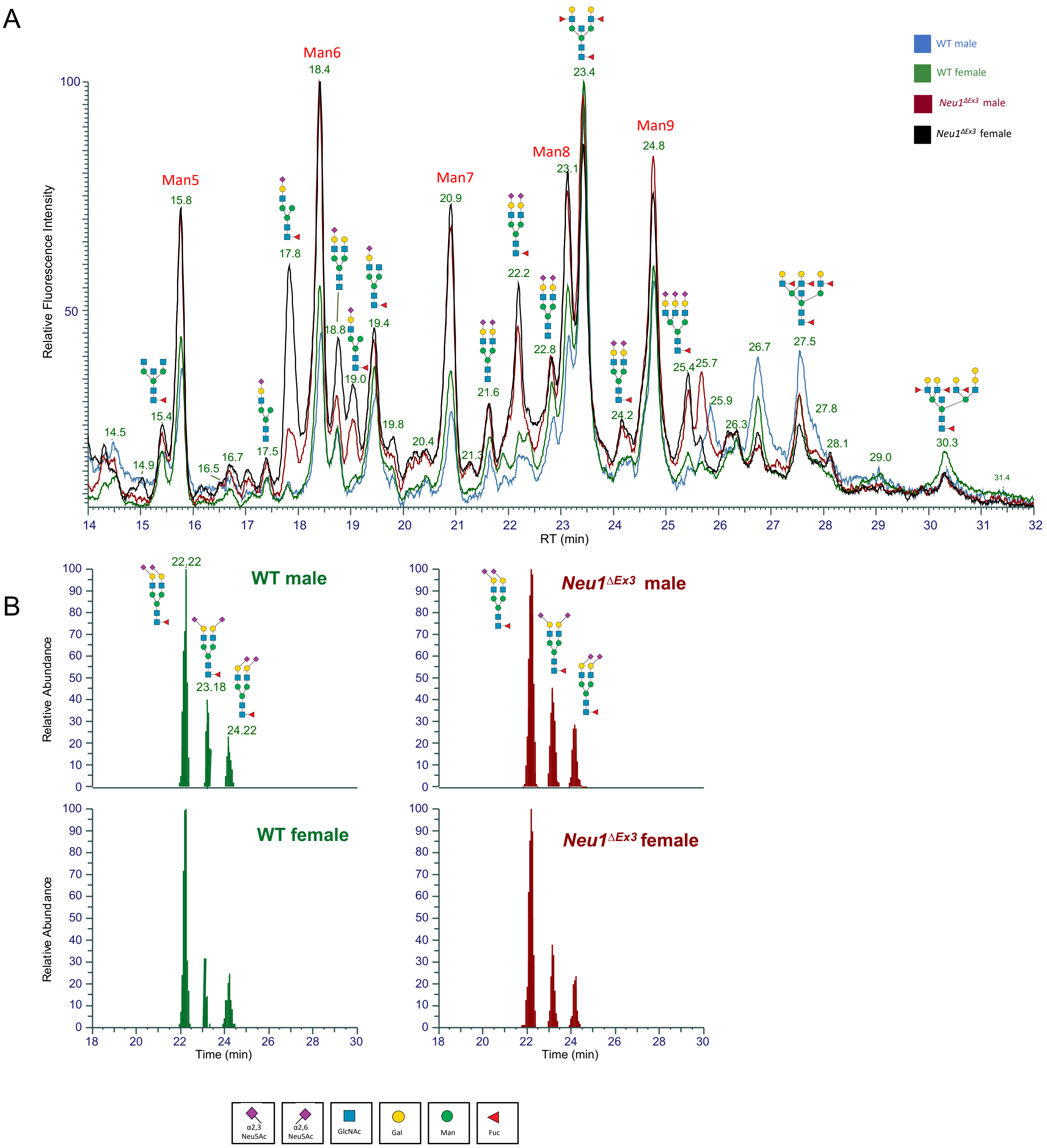

Figure S8. Analysis of sialic acid linkages in N-glycans by HILIC-UPLC-FLR-ESI-MS.
(A) Overlaid representative fluorescence (FLR) chromatograms obtained by HILIC-UPLC-FLR-MS of *Rapi*Fluor labeled kidney protein N-glycans from male and female WT and *Neu1*$^{\Delta Ex3}$. An increase of oligomannoses and biantennary core fucosylated disialo-glycoform is evident in *Neu1*$^{\Delta Ex3}$ mice of both sexes. The biantennary disialo-glycoform bearing one Neu5Gc at each antenna (retention time 25.7) is much more intense in the *Neu1*$^{\Delta Ex3}$ male mouse.
(B) Extracted ion chromatograms of the peak at *m/z* 1341.0150 (±5ppm) corresponding to the biantennary core fucosylated disialo-glycoform from four mice (WT and *Neu1*$^{\Delta Ex3}$ males and

females), showing a separation of glycans containing 2,3 and 2,6-linked sialic acids. Peaks were assigned based on the retention times of similar glyco-isomers from human serum and by comparison of chromatograms of glycans treated or not with a neuraminidase specific for 2,3-linked sialic acid residues.

Table S1. Bone analyses in WT and *Neu1*$^{\Delta Ex3}$ mice with micro-CT and histology.

**Parameters/group**	WT	*Neu1*$^{\Delta Ex3}$
**Micro-Ct (Cortical bone analyses)**		
Bone length (mm)	18.4 ± 0.3	16.5 ± 0.3
Bone volume (mm^3)	1.07 ± 0.03	0.83 ± 0.08
Total volume (mm^3)	1.44 ± 0.05	1.22 ± 0.06
Bone volume fraction (BVF)	0.75 ± 0.01	0.68 ± 0.05
Bone mineral content (BMC, mg)	1.09 ± 0.02	0.81 ± 0.08
Bone mineral density (BMD, mg/cm^3)	1015 ± 15	976 ± 11
Cortical thickness (mm)	0.18 ± 0.01	0.16 ± 0.01
**Histomorphometry (Trabecular bone analyses)**		
Bone volume (BV, mm^2)	0.14 ± 0.04	0.25 ± 0.03
Tissue volume (TV, mm^2)	2.33 ± 0.07	2.06 ± 0.02
Bone surface (BS, mm)	6.67 ± 1.68	14.68 ± 0.17
BV/TV	0.08 ± 0.02	0.14 ± 0.01
BS/TV	49.47 ± 4.23	61.50 ± 5.83
Trabecular thickness (Tb.Th, um)	41.1 ± 3.9	32.8 ± 3.1
Trabecular number (Tb.N, n/mm^2)	1.4 ± 0.3	3.7 ± 0.04
Trabecular separation (Tb.Sp, um)	782.3 ± 214.8	240.4 ± 45.9
Growth plate thickness (mm)	0.117 ± 0.053	0.108 ± 0.015
Growth plate thickness (%)	68.4 ± 1.1	49.5 ± 5.7
Growth plate volume (mm2)	0.056 ± 0.003	0.049 ± 0.002
Growth plate volume (%)	67.2 ± 1.3	50.4 ± 7.2
Chondrocyte number (n/mm^2 of growth plate)	1310 ± 113	1711 ± 87
Primary spongiosa thickness (uM)	54.1 ± 4.6	113.4 ± 27.5
Primary spongiosa thickness (%)	31.6 ± 1.1	50.5 ± 5.7
Primary spongiosa (mm2)	0.027 ± 0.003	0.051 ± 0.016
Primary spongiosa volume (%)	32.8 ± 1.3	49.6 ± 7.2
BV Primary spongiosa (mm2)	0.008 ± 0.003	0.025 ± 0.009
BV Primary spongiosa (%)	53.6 ± 10.3	48.6 ± 1.9

Table S2 Proteins reduced or increased in kidney of *Neu1*$^{\Delta Ex3}$ compared to WT mice according to the results of LC-MS/MS analysis.

List of proteins reduced in kidney of 4-month-old female *Neu1*$^{\Delta Ex3}$ mice							Exclusive Unique peptide count					
#	Identified Proteins (1841)	Accession Number	Alternate ID	MW	T-Test (p-value): ($p < 0.05$)	Quantitative Profile	KO1	KO2	KO3	WT1	WT2	WT3
1	Low-density lipoprotein receptor-related protein 2 OS=Mus musculus (Mouse) OX=10090 GN=Lrp2 PE=1 SV=1	A2ARV4	Lrp2	519 kDa	0.01	KO Female NT low, WT Female NT high	106	87	62	159	170	128
2	ATP synthase subunit beta, mitochondrial OS=Mus musculus (Mouse) OX=10090 GN=Atp5f1b PE=1 SV=2	P56480	Atp5f1b	56 kDa	0.0043	KO Female NT low, WT Female NT high	66	66	59	93	96	83
3	Peroxisomal bifunctional enzyme OS=Mus musculus (Mouse) OX=10090 GN=Ehhadh PE=1 SV=4	Q9DBM2	Ehhadh	78 kDa	0.01	KO Female NT low, WT Female NT high	52	41	41	102	108	73
4	ATP synthase subunit alpha, mitochondrial OS=Mus musculus (Mouse) OX=10090 GN=Atp5f1a PE=1 SV=1	Q03265	Atp5f1a	60 kDa	0.038	KO Female NT low, WT Female NT high	51	54	47	80	84	59
5	Catalase OS=Mus musculus (Mouse) OX=10090 GN=Cat PE=1 SV=4	P24270	Cat	60 kDa	0.027	KO Female NT low, WT Female NT high	54	44	43	65	67	61
6	Cluster of Sodium/potassium-transporting ATPase subunit alpha-1 OS=Mus musculus (Mouse) OX=10090 GN=Atp1a1 PE=1 SV=1 (Q8VDN2)	Q8VDN2 [5]	Atp1a1	113 kDa	0.037	KO Female NT low, WT Female NT high	49	32	37	49	55	51
7	Argininosuccinate synthase OS=Mus musculus (Mouse) OX=10090 GN=Ass1 PE=1 SV=1	P16460	Ass1	47 kDa	0.046	KO Female NT low, WT Female NT high	49	42	42	53	57	52

8	Pyruvate carboxylase, mitochondrial OS=Mus musculus (Mouse) OX=10090 GN=Pc PE=1 SV=1	Q05920	Pc	130 kDa	0.029	KO Female NT low, WT Female NT high	52	39	38	62	67	55
9	Glutamate dehydrogenase 1, mitochondrial OS=Mus musculus (Mouse) OX=10090 GN=Glud1 PE=1 SV=1	P26443	Glud1	61 kDa	0.027	KO Female NT low, WT Female NT high	52	47	38	54	53	54
10	Villin-1 OS=Mus musculus (Mouse) OX=10090 GN=Vil1 PE=1 SV=3	Q62468	Vil1	93 kDa	0.019	KO Female NT low, WT Female NT high	39	32	27	44	47	41
11	Sorbitol dehydrogenase OS=Mus musculus (Mouse) OX=10090 GN=Sord PE=1 SV=3	Q64442	Sord	38 kDa	0.035	KO Female NT low, WT Female NT high	35	28	28	45	49	42
12	Cluster of ADP/ATP translocase 2 OS=Mus musculus (Mouse) OX=10090 GN=Slc25a5 PE=1 SV=3 (P51881)	P51881 [2]	Slc25a5	33 kDa	0.0028	KO Female NT low, WT Female NT high	36	30	28	43	42	39
13	Cluster of Aldo-keto reductase family 1 member C21 OS=Mus musculus (Mouse) OX=10090 GN=Akr1c21 PE=1 SV=2 (Q91WR5)	Q91WR5 [3]	Akr1c21	37 kDa	0.013	KO Female NT low, WT Female NT high	22	19	17	30	32	28
14	Glycine N-acyltransferase-like protein Keg1 OS=Mus musculus (Mouse) OX=10090 GN=Keg1 PE=1 SV=1	Q9DCY0	Keg1	34 kDa	0.022	KO Female NT low, WT Female NT high	22	21	18	28	31	26
15	Kynurenine/alpha-aminoadipate aminotransferase, mitochondrial OS=Mus musculus (Mouse) OX=10090 GN=Aadat PE=1 SV=1	Q9WVM8	Aadat	48 kDa	0.032	KO Female NT low, WT Female NT high	44	20	20	57	61	41
16	2-iminobutanoate/2-iminopropanoate deaminase OS=Mus musculus (Mouse) OX=10090 GN=Rida PE=1 SV=3	P52760	Rida	14 kDa	0.021	KO Female NT low, WT Female NT high	29	25	23	39	34	30

17	N-acyl-aromatic-L-amino acid amidohydrolase (carboxylate-forming) OS=Mus musculus (Mouse) OX=10090 GN=Acy3 PE=1 SV=1	Q91XE4	Acy3	35 kDa	0.021	KO Female NT low, WT Female NT high	22	16	15	29	29	24
18	Meprin A subunit alpha OS=Mus musculus (Mouse) OX=10090 GN=Mep1a PE=1 SV=4	P28825	Mep1a	84 kDa	0.027	KO Female NT low, WT Female NT high	15	13	13	27	34	20
19	Glycine amidinotransferase, mitochondrial OS=Mus musculus (Mouse) OX=10090 GN=Gatm PE=1 SV=1	Q9D964	Gatm	48 kDa	0.046	KO Female NT low, WT Female NT high	34	29	21	32	33	30
20	Propionyl-CoA carboxylase alpha chain, mitochondrial OS=Mus musculus (Mouse) OX=10090 GN=Pcca PE=1 SV=2	Q91ZA3	Pcca	80 kDa	0.022	KO Female NT low, WT Female NT high	31	23	24	37	33	31
21	Hydroxyacid oxidase 2 OS=Mus musculus (Mouse) OX=10090 GN=Hao2 PE=1 SV=1	Q9NYQ2	Hao2	39 kDa	0.018	KO Female NT low, WT Female NT high	30	26	23	51	55	38
22	Probable D-lactate dehydrogenase, mitochondrial OS=Mus musculus (Mouse) OX=10090 GN=Ldhd PE=1 SV=1	Q7TNG8	Ldhd	52 kDa	0.0028	KO Female NT low, WT Female NT high	13	14	15	20	21	18
23	Cytochrome b-c1 complex subunit 2, mitochondrial OS=Mus musculus (Mouse) OX=10090 GN=Uqcrc2 PE=1 SV=1	Q9DB77	Uqcrc2	48 kDa	0.037	KO Female NT low, WT Female NT high	24	22	16	35	37	25
24	Cluster of Heat shock 70 kDa protein 1A OS=Mus musculus (Mouse) OX=10090 GN=Hspa1a PE=1 SV=2 (Q61696)	Q61696 [2]	Hspa1a	70 kDa	0.046	KO Female NT low, WT Female NT high	12	8	6	14	10	19
25	Fumarate hydratase, mitochondrial OS=Mus musculus (Mouse) OX=10090 GN=Fh PE=1 SV=3	P97807	Fh	54 kDa	0.00027	KO Female NT low, WT Female NT high	25	23	21	30	30	26

26	Nucleoside diphosphate-linked moiety X motif 19 OS=Mus musculus (Mouse) OX=10090 GN=Nudt19 PE=1 SV=2	P11930	Nudt19	40 kDa	0.0026	KO Female NT low, WT Female NT high	10	8	8	18	21	18
27	Triokinase/FMN cyclase OS=Mus musculus (Mouse) OX=10090 GN=Tkfc PE=1 SV=1	Q8VC30	Tkfc	60 kDa	0.033	KO Female NT low, WT Female NT high	19	9	12	24	26	20
28	Acyl-coenzyme A synthetase ACSM1, mitochondrial OS=Mus musculus (Mouse) OX=10090 GN=Acsm1 PE=1 SV=1	Q91VA0	Acsm1	65 kDa	0.016	KO Female NT low, WT Female NT high	21	14	11	30	33	27
29	Peroxisomal multifunctional enzyme type 2 OS=Mus musculus (Mouse) OX=10090 GN=Hsd17b4 PE=1 SV=3	P51660	Hsd17b4	79 kDa	0.021	KO Female NT low, WT Female NT high	15	9	9	21	17	18
30	Carboxylesterase 1D OS=Mus musculus (Mouse) OX=10090 GN=Ces1d PE=1 SV=1	Q8VCT4	Ces1d	62 kDa	0.00014	KO Female NT low, WT Female NT high	10	12	9	18	19	18
31	NADH-ubiquinone oxidoreductase 75 kDa subunit, mitochondrial OS=Mus musculus (Mouse) OX=10090 GN=Ndufs1 PE=1 SV=2	Q91VD9	Ndufs1	80 kDa	0.0085	KO Female NT low, WT Female NT high	12	14	7	22	28	22
32	Cluster of Carbonyl reductase [NADPH] 1 OS=Mus musculus (Mouse) OX=10090 GN=Cbr1 PE=1 SV=3 (P48758)	P48758 [2]	Cbr1	31 kDa	0.014	KO Female NT low, WT Female NT high	27	23	22	31	31	24
33	Heat shock protein 75 kDa, mitochondrial OS=Mus musculus (Mouse) OX=10090 GN=Trap1 PE=1 SV=1	Q9CQN1	Trap1	80 kDa	0.013	KO Female NT low, WT Female NT high	16	16	12	22	19	22
34	Ketohexokinase OS=Mus musculus (Mouse) OX=10090 GN=Khk PE=1 SV=1	P97328	Khk	33 kDa	0.02	KO Female NT low, WT Female NT high	15	11	8	21	28	26

35	Carboxylesterase 1F OS=Mus musculus (Mouse) OX=10090 GN=Ces1f PE=1 SV=1	Q91WU0	Ces1f	62 kDa	0.035	KO Female NT low, WT Female NT high	6	2	2	5	5	4
36	Valacyclovir hydrolase OS=Mus musculus (Mouse) OX=10090 GN=Bphl PE=1 SV=1	Q8R164	Bphl	33 kDa	0.0039	KO Female NT low, WT Female NT high	16	10	9	18	19	17
37	Cluster of 3-ketoacyl-CoA thiolase A, peroxisomal OS=Mus musculus (Mouse) OX=10090 GN=Acaa1a PE=1 SV=1 (Q921H8)	Q921H8 [2]	Acaa1a	44 kDa	0.048	KO Female NT low, WT Female NT high	18	15	11	21	19	21
38	Peroxisomal carnitine O-octanoyltransferase OS=Mus musculus (Mouse) OX=10090 GN=Crot PE=1 SV=1	Q9DC50	Crot	70 kDa	0.0054	KO Female NT low, WT Female NT high	4	1	0	10	9	12
39	3-hydroxyisobutyrate dehydrogenase, mitochondrial OS=Mus musculus (Mouse) OX=10090 GN=Hibadh PE=1 SV=1	Q99L13	Hibadh	35 kDa	0.033	KO Female NT low, WT Female NT high	15	18	17	22	20	18
40	Phosphate carrier protein, mitochondrial OS=Mus musculus (Mouse) OX=10090 GN=Slc25a3 PE=1 SV=1	Q8VEM8	Slc25a3	40 kDa	0.05	KO Female NT low, WT Female NT high	13	11	9	19	27	14
41	Fumarylacetoacetase OS=Mus musculus (Mouse) OX=10090 GN=Fah PE=1 SV=2	P35505	Fah	46 kDa	0.0091	KO Female NT low, WT Female NT high	17	14	14	25	24	20
42	Cadherin-16 OS=Mus musculus (Mouse) OX=10090 GN=Cdh16 PE=1 SV=1	O88338	Cdh16	90 kDa	0.032	KO Female NT low, WT Female NT high	12	13	7	14	22	15
43	Lipoamide acyltransferase component of branched-chain alpha-keto acid dehydrogenase complex, mitochondrial OS=Mus musculus (Mouse) OX=10090 GN=Dbt PE=1 SV=2	P53395	Dbt	53 kDa	0.018	KO Female NT low, WT Female NT high	12	10	8	17	16	15

44	Succinate dehydrogenase [ubiquinone] iron-sulfur subunit, mitochondrial OS=Mus musculus (Mouse) OX=10090 GN=Sdhb PE=1 SV=1	Q9CQA3	Sdhb	32 kDa	0.038	KO Female NT low, WT Female NT high	14	9	11	17	13	18
45	Electron transfer flavoprotein-ubiquinone oxidoreductase, mitochondrial OS=Mus musculus (Mouse) OX=10090 GN=Etfdh PE=1 SV=1	Q921G7	Etfdh	68 kDa	0.0022	KO Female NT low, WT Female NT high	10	7	7	17	20	15
46	Apoptosis-inducing factor 1, mitochondrial OS=Mus musculus (Mouse) OX=10090 GN=Aifm1 PE=1 SV=1	Q9Z0X1	Aifm1	67 kDa	0.012	KO Female NT low, WT Female NT high	8	10	7	17	20	12
47	Cytochrome c1, heme protein, mitochondrial OS=Mus musculus (Mouse) OX=10090 GN=Cyc1 PE=1 SV=1	Q9D0M3	Cyc1	35 kDa	0.016	KO Female NT low, WT Female NT high	8	8	6	18	17	8
48	NADH dehydrogenase [ubiquinone] flavoprotein 1, mitochondrial OS=Mus musculus (Mouse) OX=10090 GN=Ndufv1 PE=1 SV=1	Q91YT0	Ndufv1	51 kDa	0.025	KO Female NT low, WT Female NT high	11	11	6	15	20	16
49	Alpha-methylacyl-CoA racemase OS=Mus musculus (Mouse) OX=10090 GN=Amacr PE=1 SV=4	O09174	Amacr	42 kDa	0.031	KO Female NT low, WT Female NT high	4	4	4	7	6	10
50	ATP synthase subunit gamma, mitochondrial OS=Mus musculus (Mouse) OX=10090 GN=Atp5f1c PE=1 SV=1	Q91VR2	Atp5f1c	33 kDa	0.0048	KO Female NT low, WT Female NT high	10	7	9	14	14	11
51	Cytochrome c oxidase subunit 2 OS=Mus musculus (Mouse) OX=10090 GN=Mtco2 PE=1 SV=1	P00405	Mtco2	26 kDa	0.042	KO Female NT low, WT Female NT high	10	8	7	11	15	10
52	Secernin-2 OS=Mus musculus (Mouse) OX=10090 GN=Scrn2 PE=1 SV=1	Q8VCA8	Scrn2	47 kDa	0.016	KO Female NT low, WT Female NT high	9	5	5	10	10	10

53	Voltage-dependent anion-selective channel protein 2 OS=Mus musculus (Mouse) OX=10090 GN=Vdac2 PE=1 SV=2	Q60930	Vdac2	32 kDa	0.033	KO Female NT low, WT Female NT high	9	9	7	8	13	9
54	Prohibitin OS=Mus musculus (Mouse) OX=10090 GN=Phb PE=1 SV=1	P67778	Phb	30 kDa	0.0058	KO Female NT low, WT Female NT high	5	6	4	18	15	12
55	3-hydroxyanthranilate 3,4-dioxygenase OS=Mus musculus (Mouse) OX=10090 GN=Haao PE=1 SV=1	Q78JT3	Haao	33 kDa	0.015	KO Female NT low, WT Female NT high	6	6	3	11	8	10
56	ATP-binding cassette sub-family D member 3 OS=Mus musculus (Mouse) OX=10090 GN=Abcd3 PE=1 SV=2	P55096	Abcd3	75 kDa	0.01	KO Female NT low, WT Female NT high	5	1	0	9	12	9
57	NADPH--cytochrome P450 reductase OS=Mus musculus (Mouse) OX=10090 GN=Por PE=1 SV=2	P37040	Por	77 kDa	0.03	KO Female NT low, WT Female NT high	7	4	3	7	11	10
58	Xaa-Pro aminopeptidase 1 OS=Mus musculus (Mouse) OX=10090 GN=Xpnpep1 PE=1 SV=1	Q6P1B1	Xpnpep1	70 kDa	0.038	KO Female NT low, WT Female NT high	7	4	6	9	10	12
59	Complex I assembly factor ACAD9, mitochondrial OS=Mus musculus (Mouse) OX=10090 GN=Acad9 PE=1 SV=2	Q8JZN5	Acad9	69 kDa	0.031	KO Female NT low, WT Female NT high	6	3	3	7	8	6
60	Prohibitin-2 OS=Mus musculus (Mouse) OX=10090 GN=Phb2 PE=1 SV=1	O35129	Phb2	33 kDa	0.031	KO Female NT low, WT Female NT high	3	6	4	10	10	7
61	NADH dehydrogenase [ubiquinone] 1 alpha subcomplex subunit 12 OS=Mus musculus (Mouse) OX=10090 GN=Ndufa12 PE=1 SV=2	Q7TMF3	Ndufa12	17 kDa	0.033	KO Female NT low, WT Female NT high	6	5	4	8	6	5

62	Enoyl-CoA delta isomerase 3, peroxisomal OS=Mus musculus (Mouse) OX=10090 GN=Eci3 PE=1 SV=1	Q78JN3	Eci3	35 kDa	0.022	KO Female NT low, WT Female NT high	3	3	2	5	6	4
63	NADH dehydrogenase [ubiquinone] iron-sulfur protein 3, mitochondrial OS=Mus musculus (Mouse) OX=10090 GN=Ndufs3 PE=1 SV=2	Q9DCT2	Ndufs3	30 kDa	0.042	KO Female NT low, WT Female NT high	3	4	2	10	13	6
64	Cluster of NADPH-dependent 3-keto-steroid reductase Hsd3b4 OS=Mus musculus (Mouse) OX=10090 GN=Hsd3b4 PE=1 SV=3 (Q61767)	Q61767 [2]	Hsd3b4	42 kDa	0.01	KO Female NT low, WT Female NT high	3	1	1	7	9	10
65	Mitochondrial amidoxime reducing component 2 OS=Mus musculus (Mouse) OX=10090 GN=Mtarc2 PE=1 SV=1	Q922Q1	Mtarc2	38 kDa	0.018	KO Female NT low, WT Female NT high	4	2	3	7	8	4
66	Calcium-binding mitochondrial carrier protein Aralar1 OS=Mus musculus (Mouse) OX=10090 GN=Slc25a12 PE=1 SV=1	Q8BH59	Slc25a12	75 kDa	0.023	KO Female NT low, WT Female NT high	0	1	0	4	9	7
67	40S ribosomal protein S14 OS=Mus musculus (Mouse) OX=10090 GN=Rps14 PE=1 SV=3	P62264	Rps14	16 kDa	0.008	KO Female NT low, WT Female NT high	5	3	3	7	5	7
68	Sideroflexin-1 OS=Mus musculus (Mouse) OX=10090 GN=Sfxn1 PE=1 SV=3	Q99JR1	Sfxn1	36 kDa	0.039	KO Female NT low, WT Female NT high	3	1	1	4	7	5
69	EH domain-containing protein 4 OS=Mus musculus (Mouse) OX=10090 GN=Ehd4 PE=1 SV=1	Q9EQP2	Ehd4	61 kDa	0.023	KO Female NT low, WT Female NT high	3	1	1	4	4	3
70	CDGSH iron-sulfur domain-containing protein 1 OS=Mus musculus (Mouse) OX=10090 GN=Cisd1 PE=1 SV=1	Q91WS0	Cisd1	12 kDa	0.0022	KO Female NT low, WT Female NT high	2	3	3	5	5	4

71	Enoyl-[acyl-carrier-protein] reductase, mitochondrial OS=Mus musculus (Mouse) OX=10090 GN=Mecr PE=1 SV=2	Q9DCS3	Mecr	40 kDa	0.015	KO Female NT low, WT Female NT high	2	1	0	5	4	5
72	Sulfide:quinone oxidoreductase, mitochondrial OS=Mus musculus (Mouse) OX=10090 GN=Sqor PE=1 SV=3	Q9R112	Sqor	50 kDa	0.018	KO Female NT low, WT Female NT high	2	1	1	6	4	5
73	Mitochondrial 2-oxoglutarate/malate carrier protein OS=Mus musculus (Mouse) OX=10090 GN=Slc25a11 PE=1 SV=3	Q9CR62	Slc25a11	34 kDa	0.013	KO Female NT low, WT Female NT high	4	3	3	7	9	7
74	Liver carboxylesterase 1 OS=Mus musculus (Mouse) OX=10090 GN=Ces1 PE=1 SV=1	Q8VCC2	Ces1	63 kDa	0.00039	KO Female NT low, WT Female NT high	0	0	0	0	0	0
75	Acyl-coenzyme A amino acid N-acyltransferase 1 OS=Mus musculus (Mouse) OX=10090 GN=Acnat1 PE=1 SV=1	A2AKK5	Acnat1	46 kDa	0.011	KO Female NT low, WT Female NT high	3	0	0	10	9	7
76	NADH dehydrogenase [ubiquinone] 1 alpha subcomplex subunit 5 OS=Mus musculus (Mouse) OX=10090 GN=Ndufa5 PE=1 SV=3	Q9CPP6	Ndufa5	13 kDa	0.0072	KO Female NT low, WT Female NT high	0	1	2	6	5	3
77	60S ribosomal protein L28 OS=Mus musculus (Mouse) OX=10090 GN=Rpl28 PE=1 SV=2	P41105	Rpl28	16 kDa	0.0031	KO Female NT low, WT Female NT high	2	2	1	4	5	4
78	Actin-related protein 2/3 complex subunit 1A OS=Mus musculus (Mouse) OX=10090 GN=Arpc1a PE=1 SV=1	Q9R0Q6	Arpc1a	42 kDa	0.0053	KO Female NT low, WT Female NT high	1	0	0	2	4	5
79	NADH dehydrogenase [ubiquinone] 1 beta subcomplex subunit 4 OS=Mus musculus (Mouse) OX=10090 GN=Ndufb4 PE=1 SV=3	Q9CQC7	Ndufb4	15 kDa	0.047	KO Female NT low, WT Female NT high	2	3	2	4	3	4

80	Voltage-dependent anion-selective channel protein 3 OS=Mus musculus (Mouse) OX=10090 GN=Vdac3 PE=1 SV=1	Q60931	Vdac3	31 kDa	0.0038	KO Female NT low, WT Female NT high	0	2	0	5	5	3
81	NADH dehydrogenase [ubiquinone] iron-sulfur protein 5 OS=Mus musculus (Mouse) OX=10090 GN=Ndufs5 PE=1 SV=3	Q99LY9	Ndufs5	13 kDa	0.039	KO Female NT low, WT Female NT high	2	2	2	4	3	3
82	Ectonucleoside triphosphate diphosphohydrolase 5 OS=Mus musculus (Mouse) OX=10090 GN=Entpd5 PE=1 SV=1	Q9WUZ9	Entpd5	47 kDa	0.016	KO Female NT low, WT Female NT high	2	1	0	3	4	3
83	NADH dehydrogenase [ubiquinone] 1 beta subcomplex subunit 5, mitochondrial OS=Mus musculus (Mouse) OX=10090 GN=Ndufb5 PE=1 SV=1	Q9CQH3	Ndufb5	22 kDa	0.031	KO Female NT low, WT Female NT high	1	2	0	5	5	3
84	NADH dehydrogenase [ubiquinone] 1 beta subcomplex subunit 8, mitochondrial OS=Mus musculus (Mouse) OX=10090 GN=Ndufb8 PE=1 SV=1	Q9D6J5	Ndufb8	22 kDa	0.021	KO Female NT low, WT Female NT high	0	2	1	2	3	2
85	Mitochondrial pyruvate carrier 2 OS=Mus musculus (Mouse) OX=10090 GN=Mpc2 PE=1 SV=1	Q9D023	Mpc2	14 kDa	0.047	KO Female NT low, WT Female NT high	2	1	1	2	3	3
86	NADH dehydrogenase [ubiquinone] iron-sulfur protein 8, mitochondrial OS=Mus musculus (Mouse) OX=10090 GN=Ndufs8 PE=1 SV=1	Q8K3J1	Ndufs8	24 kDa	0.016	KO Female NT low, WT Female NT high	1	1	1	2	2	3
87	NADH dehydrogenase [ubiquinone] 1 alpha subcomplex subunit 6 OS=Mus musculus (Mouse) OX=10090 GN=Ndufa6 PE=1 SV=1	Q9CQZ5	Ndufa6	15 kDa	0.016	KO Female NT low, WT Female NT high	1	2	1	5	4	3
88	DnaJ homolog subfamily A member 1 OS=Mus musculus (Mouse) OX=10090 GN=Dnaja1 PE=1 SV=1	P63037	Dnaja1	45 kDa	0.023	KO Female NT low, WT Female NT high	2	0	0	2	2	4

89	Glycine dehydrogenase (decarboxylating), mitochondrial OS=Mus musculus (Mouse) OX=10090 GN=Gldc PE=1 SV=1	Q91W43	Gldc	113 kDa	0.038	KO Female NT low, WT Female NT high	2	0	0	7	8	3
90	Transmembrane emp24 domain-containing protein 10 OS=Mus musculus (Mouse) OX=10090 GN=Tmed10 PE=1 SV=1	Q9D1D4	Tmed10	25 kDa	0.013	KO Female NT low, WT Female NT high	1	2	1	2	4	4
91	NADH dehydrogenase [ubiquinone] 1 alpha subcomplex subunit 2 OS=Mus musculus (Mouse) OX=10090 GN=Ndufa2 PE=1 SV=3	Q9CQ75	Ndufa2	11 kDa	0.016	KO Female NT low, WT Female NT high	1	1	1	2	2	3
92	Solute carrier family 22 member 2 OS=Mus musculus (Mouse) OX=10090 GN=Slc22a2 PE=1 SV=1	O70577	Slc22a2	62 kDa	0.026	KO Female NT low, WT Female NT high	0	0	0	1	2	3
93	4-hydroxyphenylpyruvate dioxygenase OS=Mus musculus (Mouse) OX=10090 GN=Hpd PE=1 SV=3	P49429	Hpd	45 kDa	0.008	KO Female NT low, WT Female NT high	0	0	0	4	3	5
94	Solute carrier family 22 member 12 OS=Mus musculus (Mouse) OX=10090 GN=Slc22a12 PE=1 SV=1	Q8CFZ5	Slc22a12	60 kDa	0.025	KO Female NT low, WT Female NT high	0	0	0	2	3	1
95	Lysine--tRNA ligase OS=Mus musculus (Mouse) OX=10090 GN=Kars1 PE=1 SV=1	Q99MN1	Kars1	68 kDa	0.016	KO Female NT low, WT Female NT high	1	0	1	2	2	2
96	Dehydrogenase/reductase SDR family member 1 OS=Mus musculus (Mouse) OX=10090 GN=Dhrs1 PE=1 SV=1	Q99L04	Dhrs1	34 kDa	0.047	KO Female NT low, WT Female NT high	1	0	0	2	2	1
97	Acyl-coenzyme A thioesterase 8 OS=Mus musculus (Mouse) OX=10090 GN=Acot8 PE=1 SV=1	P58137	Acot8	36 kDa	0.047	KO Female NT low, WT Female NT high	1	0	0	1	2	2

98	Mitochondrial pyruvate carrier 1 OS=Mus musculus (Mouse) OX=10090 GN=Mpc1 PE=1 SV=1	P63030	Mpc1	12 kDa	0.0075	KO Female NT low, WT Female NT high	0	1	0	2	2	2
99	Serine beta-lactamase-like protein LACTB, mitochondrial OS=Mus musculus (Mouse) OX=10090 GN=Lactb PE=1 SV=1	Q9EP89	Lactb	61 kDa	0.026	KO Female NT low, WT Female NT high	0	0	0	2	1	1
100	Vesicular integral-membrane protein VIP36 OS=Mus musculus (Mouse) OX=10090 GN=Lman2 PE=1 SV=2	Q9DBH5	Lman2	40 kDa	0.016	KO Female NT low, WT Female NT high	0	0	0	1	2	1
101	Arginase-2, mitochondrial OS=Mus musculus OX=10090 GN=Arg2 PE=1 SV=1	O08691		40 kDa	0.016	KO Female NT low, WT Female NT high	0	0	0	2	1	1

List of proteins increased in kidney of 4-month-old female *Neu1*$^{\Delta Ex3}$ mice.							Exclusive unique peptide count					
#	Identified Proteins (1841)	Accession Number	Alternate ID	MW	T-Test (p-value): ($p < 0.05$)	Quantitative Profile	KO1	KO2	KO3	WT1	WT2	WT3
1	Albumin OS=Mus musculus (Mouse) OX=10090 GN=Alb PE=1 SV=3	P07724	Alb	69 kDa	0.035	KO Female NT high, WT Female NT low	219	203	201	212	212	195
2	Spectrin alpha chain, non-erythrocytic 1 OS=Mus musculus (Mouse) OX=10090 GN=Sptan1 PE=1 SV=4	P16546	Sptan1	285 kDa	0.0059	KO Female NT high, WT Female NT low	125	133	132	109	114	96
3	Spectrin beta chain, non-erythrocytic 1 OS=Mus musculus (Mouse) OX=10090 GN=Sptbn1 PE=1 SV=2	Q62261	Sptbn1	274 kDa	0.027	KO Female NT high, WT Female NT low	83	85	87	66	70	75
4	Cluster of Lysozyme C-2 OS=Mus musculus (Mouse) OX=10090 GN=Lyz2 PE=1 SV=2 (P08905)	P08905 [2]	Lyz2	17 kDa	0.00079	KO Female NT high, WT Female NT low	64	52	60	10	4	3
5	Cluster of Tubulin alpha-1C chain OS=Mus musculus (Mouse) OX=10090 GN=Tuba1c PE=1 SV=1 (P68373)	P68373 [5]	Tuba1c	50 kDa	0.038	KO Female NT high, WT Female NT low	45	48	46	40	43	32
6	Filamin-B OS=Mus musculus (Mouse) OX=10090 GN=Flnb PE=1 SV=3	Q80X90	Flnb	278 kDa	0.037	KO Female NT high, WT Female NT low	50	63	67	31	28	47
7	Peptidyl-prolyl cis-trans isomerase A OS=Mus musculus (Mouse) OX=10090 GN=Ppia PE=1 SV=2	P17742	Ppia	18 kDa	0.044	KO Female NT high, WT Female NT low	39	42	39	33	26	35
8	Transketolase OS=Mus musculus (Mouse) OX=10090 GN=Tkt PE=1 SV=1	P40142	Tkt	68 kDa	0.012	KO Female NT high, WT Female NT low	37	38	41	35	36	35

9	Talin-1 OS=Mus musculus (Mouse) OX=10090 GN=Tln1 PE=1 SV=2	P26039	Tln1	270 kDa	0.027	KO Female NT high, WT Female NT low	37	36	45	23	21	33
10	Filamin-A OS=Mus musculus (Mouse) OX=10090 GN=Flna PE=1 SV=5	Q8BTM8	Flna	281 kDa	0.0055	KO Female NT high, WT Female NT low	45	40	57	21	18	25
11	Phosphatidylethanolamine-binding protein 1 OS=Mus musculus (Mouse) OX=10090 GN=Pebp1 PE=1 SV=3	P70296	Pebp1	21 kDa	0.0038	KO Female NT high, WT Female NT low	35	39	39	29	25	28
12	Vinculin OS=Mus musculus (Mouse) OX=10090 GN=Vcl PE=1 SV=4	Q64727	Vcl	117 kDa	0.011	KO Female NT high, WT Female NT low	38	40	30	20	21	28
13	Cathepsin D OS=Mus musculus (Mouse) OX=10090 GN=Ctsd PE=1 SV=1	P18242	Ctsd	45 kDa	0.00037	KO Female NT high, WT Female NT low	28	24	29	9	8	8
14	Progranulin OS=Mus musculus (Mouse) OX=10090 GN=Grn PE=1 SV=2	P28798	Grn	63 kDa	0.001	KO Female NT high, WT Female NT low	37	35	43	15	10	8
15	Cathepsin B OS=Mus musculus (Mouse) OX=10090 GN=Ctsb PE=1 SV=2	P10605	Ctsb	37 kDa	0.00018	KO Female NT high, WT Female NT low	36	31	35	11	10	9
16	Fatty acid-binding protein, adipocyte OS=Mus musculus (Mouse) OX=10090 GN=Fabp4 PE=1 SV=3	P04117	Fabp4	15 kDa	0.03	KO Female NT high, WT Female NT low	23	25	23	18	18	20
17	Serotransferrin OS=Mus musculus (Mouse) OX=10090 GN=Tf PE=1 SV=1	Q921I1	Tf	77 kDa	0.01	KO Female NT high, WT Female NT low	31	34	32	15	23	18
18	Uromodulin OS=Mus musculus (Mouse) OX=10090 GN=Umod PE=1 SV=1	Q91X17	Umod	71 kDa	0.00052	KO Female NT high, WT	37	27	34	5	4	1

						Female NT low						
19	Beta-mannosidase OS=Mus musculus (Mouse) OX=10090 GN=Manba PE=1 SV=1	Q8K2I4	Manba	101 kDa	0.0036	KO Female NT high, WT Female NT low	35	26	40	0	2	0
20	14-3-3 protein theta OS=Mus musculus (Mouse) OX=10090 GN=Ywhaq PE=1 SV=1	P68254	Ywhaq	28 kDa	0.013	KO Female NT high, WT Female NT low	13	11	11	11	9	9
21	Ganglioside GM2 activator OS=Mus musculus (Mouse) OX=10090 GN=Gm2a PE=1 SV=2	Q60648	Gm2a	21 kDa	0.015	KO Female NT high, WT Female NT low	20	22	22	10	10	6
22	Parkinson disease protein 7 homolog OS=Mus musculus (Mouse) OX=10090 GN=Park7 PE=1 SV=1	Q99LX0	Park7	20 kDa	0.0018	KO Female NT high, WT Female NT low	20	21	20	15	13	16
23	Kininogen-1 OS=Mus musculus (Mouse) OX=10090 GN=Kng1 PE=1 SV=1	O08677	Kng1	73 kDa	0.029	KO Female NT high, WT Female NT low	21	24	30	14	15	16
24	Plastin-2 OS=Mus musculus (Mouse) OX=10090 GN=Lcp1 PE=1 SV=4	Q61233	Lcp1	70 kDa	0.0041	KO Female NT high, WT Female NT low	27	20	19	4	7	8
25	Pro-cathepsin H OS=Mus musculus (Mouse) OX=10090 GN=Ctsh PE=1 SV=2	P49935	Ctsh	37 kDa	0.006	KO Female NT high, WT Female NT low	20	21	25	13	12	9
26	Annexin A5 OS=Mus musculus (Mouse) OX=10090 GN=Anxa5 PE=1 SV=1	P48036	Anxa5	36 kDa	0.0023	KO Female NT high, WT Female NT low	18	19	16	13	13	13
27	Apolipoprotein A-IV OS=Mus musculus (Mouse) OX=10090 GN=Apoa4 PE=1 SV=3	P06728	Apoa4	45 kDa	0.022	KO Female NT high, WT Female NT low	17	22	19	13	7	11

28	Adenylyl cyclase-associated protein 1 OS=Mus musculus (Mouse) OX=10090 GN=Cap1 PE=1 SV=4	P40124	Cap1	52 kDa	0.0088	KO Female NT high, WT Female NT low	21	18	21	14	9	15
29	Transaldolase OS=Mus musculus (Mouse) OX=10090 GN=Taldo1 PE=1 SV=2	Q93092	Taldo1	37 kDa	0.008	KO Female NT high, WT Female NT low	21	19	16	13	11	14
30	Galectin-1 OS=Mus musculus (Mouse) OX=10090 GN=Lgals1 PE=1 SV=3	P16045	Lgals1	15 kDa	0.044	KO Female NT high, WT Female NT low	14	17	23	9	5	9
31	Cluster of Myosin regulatory light chain 12B OS=Mus musculus (Mouse) OX=10090 GN=Myl12b PE=1 SV=2 (Q3THE2)	Q3THE2 [2]	Myl12b	20 kDa	0.015	KO Female NT high, WT Female NT low	18	21	21	15	13	15
32	Vitamin D-binding protein OS=Mus musculus (Mouse) OX=10090 GN=Gc PE=1 SV=2	P21614	Gc	54 kDa	0.027	KO Female NT high, WT Female NT low	17	15	14	16	10	11
33	WD repeat-containing protein 1 OS=Mus musculus (Mouse) OX=10090 GN=Wdr1 PE=1 SV=3	O88342	Wdr1	66 kDa	0.0061	KO Female NT high, WT Female NT low	14	14	11	15	11	11
34	10 kDa heat shock protein, mitochondrial OS=Mus musculus (Mouse) OX=10090 GN=Hspe1 PE=1 SV=2	Q64433	Hspe1	11 kDa	0.019	KO Female NT high, WT Female NT low	14	16	16	13	10	13
35	Ceruloplasmin OS=Mus musculus (Mouse) OX=10090 GN=Cp PE=1 SV=2	Q61147	Cp	121 kDa	0.036	KO Female NT high, WT Female NT low	17	18	24	12	7	7
36	Destrin OS=Mus musculus (Mouse) OX=10090 GN=Dstn PE=1 SV=3	Q9R0P5	Dstn	19 kDa	0.018	KO Female NT high, WT Female NT low	13	16	16	10	8	13
37	Cathepsin Z OS=Mus musculus (Mouse) OX=10090 GN=Ctsz PE=1 SV=1	Q9WUU7	Ctsz	34 kDa	0.00072	KO Female NT high, WT	16	17	17	6	4	4

						Female NT low						
38	Nascent polypeptide-associated complex subunit alpha, muscle-specific form OS=Mus musculus (Mouse) OX=10090 GN=Naca PE=1 SV=2	P70670	Naca	220 kDa	0.01	KO Female NT high, WT Female NT low	7	7	9	5	3	6
39	Fatty acid-binding protein 5 OS=Mus musculus (Mouse) OX=10090 GN=Fabp5 PE=1 SV=3	Q05816	Fabp5	15 kDa	0.008	KO Female NT high, WT Female NT low	16	17	20	5	6	7
40	Cysteine and glycine-rich protein 1 OS=Mus musculus (Mouse) OX=10090 GN=Csrp1 PE=1 SV=3	P97315	Csrp1	21 kDa	0.013	KO Female NT high, WT Female NT low	11	11	15	9	5	9
41	NSFL1 cofactor p47 OS=Mus musculus (Mouse) OX=10090 GN=Nsfl1c PE=1 SV=1	Q9CZ44	Nsfl1c	41 kDa	0.02	KO Female NT high, WT Female NT low	13	20	20	13	9	9
42	Talin-2 OS=Mus musculus (Mouse) OX=10090 GN=Tln2 PE=1 SV=3	Q71LX4	Tln2	254 kDa	0.012	KO Female NT high, WT Female NT low	4	1	5	1	0	0
43	Carboxypeptidase Q OS=Mus musculus (Mouse) OX=10090 GN=Cpq PE=1 SV=1	Q9WVJ3	Cpq	52 kDa	0.0023	KO Female NT high, WT Female NT low	10	10	14	2	0	1
44	Filamin-C OS=Mus musculus (Mouse) OX=10090 GN=Flnc PE=1 SV=3	Q8VHX6	Flnc	291 kDa	0.034	KO Female NT high, WT Female NT low	0	0	2	0	0	0
45	Proteasome activator complex subunit 1 OS=Mus musculus (Mouse) OX=10090 GN=Psme1 PE=1 SV=2	P97371	Psme1	29 kDa	0.049	KO Female NT high, WT Female NT low	11	11	10	11	5	6
46	Band 4.1-like protein 2 OS=Mus musculus (Mouse) OX=10090 GN=Epb41l2 PE=1 SV=2	O70318	Epb41l2	110 kDa	0.0085	KO Female NT high, WT Female NT low	8	12	16	8	2	2

47	Tripeptidyl-peptidase 1 OS=Mus musculus (Mouse) OX=10090 GN=Tpp1 PE=1 SV=2	O89023	Tpp1	61 kDa	0.015	KO Female NT high, WT Female NT low	12	9	10	3	8	4
48	Synaptic vesicle membrane protein VAT-1 homolog OS=Mus musculus (Mouse) OX=10090 GN=Vat1 PE=1 SV=3	Q62465	Vat1	43 kDa	0.0041	KO Female NT high, WT Female NT low	19	14	18	1	1	4
49	Annexin A4 OS=Mus musculus (Mouse) OX=10090 GN=Anxa4 PE=1 SV=4	P97429	Anxa4	36 kDa	0.0061	KO Female NT high, WT Female NT low	11	12	10	7	8	5
50	Protein kinase C and casein kinase substrate in neurons protein 2 OS=Mus musculus (Mouse) OX=10090 GN=Pacsin2 PE=1 SV=1	Q9WVE8	Pacsin2	56 kDa	0.027	KO Female NT high, WT Female NT low	12	11	8	8	3	6
51	N-acetylglucosamine-6-sulfatase OS=Mus musculus (Mouse) OX=10090 GN=Gns PE=1 SV=1	Q8BFR4	Gns	61 kDa	0.00016	KO Female NT high, WT Female NT low	15	13	17	0	0	0
52	Legumain OS=Mus musculus (Mouse) OX=10090 GN=Lgmn PE=1 SV=1	O89017	Lgmn	49 kDa	0.024	KO Female NT high, WT Female NT low	9	8	10	3	5	4
53	Myosin light chain kinase, smooth muscle OS=Mus musculus (Mouse) OX=10090 GN=Mylk PE=1 SV=3	Q6PDN3	Mylk	213 kDa	0.015	KO Female NT high, WT Female NT low	6	8	10	1	3	1
54	Arylsulfatase B OS=Mus musculus (Mouse) OX=10090 GN=Arsb PE=1 SV=3	P50429	Arsb	60 kDa	0.0028	KO Female NT high, WT Female NT low	12	9	12	1	0	1
55	UDP-N-acetylhexosamine pyrophosphorylase-like protein 1 OS=Mus musculus (Mouse) OX=10090 GN=Uap1l1 PE=1 SV=1	Q3TW96	Uap1l1	57 kDa	0.00039	KO Female NT high, WT Female NT low	14	12	12	0	1	0

56	Heterogeneous nuclear ribonucleoproteins C1/C2 OS=Mus musculus (Mouse) OX=10090 GN=Hnrnpc PE=1 SV=1	Q9Z204	Hnrnpc	34 kDa	0.019	KO Female NT high, WT Female NT low	8	8	9	5	1	5
57	Retinoid-inducible serine carboxypeptidase OS=Mus musculus (Mouse) OX=10090 GN=Scpep1 PE=1 SV=2	Q920A5	Scpep1	51 kDa	< 0.00010	KO Female NT high, WT Female NT low	14	13	15	2	1	1
58	Lysosome-associated membrane glycoprotein 1 OS=Mus musculus (Mouse) OX=10090 GN=Lamp1 PE=1 SV=2	P11438	Lamp1	44 kDa	0.00039	KO Female NT high, WT Female NT low	10	7	9	1	2	0
59	Procathepsin L OS=Mus musculus (Mouse) OX=10090 GN=Ctsl PE=1 SV=2	P06797	Ctsl	38 kDa	0.0054	KO Female NT high, WT Female NT low	8	16	16	2	1	1
60	Beta-hexosaminidase subunit beta OS=Mus musculus (Mouse) OX=10090 GN=Hexb PE=1 SV=2	P20060	Hexb	61 kDa	0.0014	KO Female NT high, WT Female NT low	12	9	12	0	0	0
61	NAD(P)H dehydrogenase [quinone] 1 OS=Mus musculus (Mouse) OX=10090 GN=Nqo1 PE=1 SV=3	Q64669	Nqo1	31 kDa	0.015	KO Female NT high, WT Female NT low	6	5	4	3	1	2
62	Glutathione peroxidase 3 OS=Mus musculus (Mouse) OX=10090 GN=Gpx3 PE=1 SV=2	P46412	Gpx3	25 kDa	0.017	KO Female NT high, WT Female NT low	8	9	7	7	5	4
63	Galectin-3 OS=Mus musculus (Mouse) OX=10090 GN=Lgals3 PE=1 SV=3	P16110	Lgals3	28 kDa	0.015	KO Female NT high, WT Female NT low	14	9	9	0	1	3
64	Lysosomal protective protein OS=Mus musculus (Mouse) OX=10090 GN=Ctsa PE=1 SV=1	P16675	Ctsa	54 kDa	0.033	KO Female NT high, WT Female NT low	6	8	5	5	2	4
65	Acid ceramidase OS=Mus musculus (Mouse) OX=10090 GN=Asah1 PE=1 SV=1	Q9WV54	Asah1	45 kDa	0.0021	KO Female NT high, WT	15	10	14	0	0	1

						Female NT low						
66	Cysteine and glycine-rich protein 2 OS=Mus musculus (Mouse) OX=10090 GN=Csrp2 PE=1 SV=3	P97314	Csrp2	21 kDa	0.033	KO Female NT high, WT Female NT low	7	6	6	6	3	4
67	N-acetylneuraminate lyase OS=Mus musculus (Mouse) OX=10090 GN=Npl PE=1 SV=1	Q9DCJ9	Npl	35 kDa	0.026	KO Female NT high, WT Female NT low	8	8	7	5	5	5
68	High mobility group nucleosome-binding domain-containing protein 5 OS=Mus musculus (Mouse) OX=10090 GN=Hmgn5 PE=1 SV=2	Q9JL35	Hmgn5	45 kDa	0.02	KO Female NT high, WT Female NT low	5	6	7	3	3	6
69	Clusterin OS=Mus musculus (Mouse) OX=10090 GN=Clu PE=1 SV=1	Q06890	Clu	52 kDa	0.028	KO Female NT high, WT Female NT low	6	5	11	2	1	1
70	Cofilin-2 OS=Mus musculus (Mouse) OX=10090 GN=Cfl2 PE=1 SV=1	P45591	Cfl2	19 kDa	0.035	KO Female NT high, WT Female NT low	3	3	3	3	2	0
71	Lysosomal alpha-mannosidase OS=Mus musculus (Mouse) OX=10090 GN=Man2b1 PE=1 SV=4	O09159	Man2b1	115 kDa	0.0015	KO Female NT high, WT Female NT low	9	11	13	0	0	0
72	Beta-galactosidase OS=Mus musculus (Mouse) OX=10090 GN=Glb1 PE=1 SV=1	P23780	Glb1	73 kDa	0.0013	KO Female NT high, WT Female NT low	10	12	9	0	1	0
73	26S proteasome non-ATPase regulatory subunit 9 OS=Mus musculus (Mouse) OX=10090 GN=Psmd9 PE=1 SV=1	Q9CR00	Psmd9	25 kDa	0.035	KO Female NT high, WT Female NT low	7	8	9	6	3	3
74	Basement membrane-specific heparan sulfate proteoglycan core protein OS=Mus musculus (Mouse) OX=10090 GN=Hspg2 PE=1 SV=1	Q05793	Hspg2	398 kDa	0.031	KO Female NT high, WT Female NT low	7	4	4	2	2	0

75	Coronin-1C OS=Mus musculus (Mouse) OX=10090 GN=Coro1c PE=1 SV=2	Q9WUM4	Coro1c	53 kDa	0.036	KO Female NT high, WT Female NT low	7	6	5	5	4	3
76	Four and a half LIM domains protein 2 OS=Mus musculus (Mouse) OX=10090 GN=Fhl2 PE=1 SV=1	O70433	Fhl2	32 kDa	0.045	KO Female NT high, WT Female NT low	7	11	14	6	0	3
77	Acid sphingomyelinase-like phosphodiesterase 3a OS=Mus musculus (Mouse) OX=10090 GN=Smpdl3a PE=1 SV=2	P70158	Smpdl3a	50 kDa	0.00039	KO Female NT high, WT Female NT low	11	9	7	0	0	0
78	Lysosomal alpha-glucosidase OS=Mus musculus (Mouse) OX=10090 GN=Gaa PE=1 SV=2	P70699	Gaa	106 kDa	0.0021	KO Female NT high, WT Female NT low	8	6	6	1	0	1
79	Heterogeneous nuclear ribonucleoprotein M OS=Mus musculus (Mouse) OX=10090 GN=Hnrnpm PE=1 SV=3	Q9D0E1	Hnrnpm	78 kDa	0.0075	KO Female NT high, WT Female NT low	5	5	6	3	2	3
80	Alpha-N-acetylgalactosaminidase OS=Mus musculus (Mouse) OX=10090 GN=Naga PE=1 SV=2	Q9QWR8	Naga	47 kDa	0.00056	KO Female NT high, WT Female NT low	6	6	8	0	0	0
81	LIM domain and actin-binding protein 1 OS=Mus musculus (Mouse) OX=10090 GN=Lima1 PE=1 SV=3	Q9ERG0	Lima1	84 kDa	0.025	KO Female NT high, WT Female NT low	4	10	10	1	1	0
82	Fibrillin-1 OS=Mus musculus (Mouse) OX=10090 GN=Fbn1 PE=1 SV=2	Q61554	Fbn1	312 kDa	0.0011	KO Female NT high, WT Female NT low	5	7	5	0	0	1
83	Gamma-interferon-inducible lysosomal thiol reductase OS=Mus musculus (Mouse) OX=10090 GN=Ifi30 PE=1 SV=3	Q9ESY9	Ifi30	28 kDa	0.00039	KO Female NT high, WT Female NT low	10	9	7	0	0	0

84	Cluster of Lamina-associated polypeptide 2, isoforms alpha/zeta OS=Mus musculus (Mouse) OX=10090 GN=Tmpo PE=1 SV=4 (Q61033)	Q61033 [2]	Tmpo	75 kDa	0.0065	KO Female NT high, WT Female NT low	1	8	1	2	3	3
85	Cluster of ADP-ribosylation factor-like protein 8B OS=Mus musculus (Mouse) OX=10090 GN=Arl8b PE=1 SV=1 (Q9CQW2)	Q9CQW2 [2]	Arl8b	22 kDa	0.033	KO Female NT high, WT Female NT low	7	6	1	2	2	1
86	Dipeptidyl peptidase 2 OS=Mus musculus (Mouse) OX=10090 GN=Dpp7 PE=1 SV=2	Q9ET22	Dpp7	56 kDa	0.00039	KO Female NT high, WT Female NT low	10	8	8	0	0	0
87	Syntaxin-7 OS=Mus musculus (Mouse) OX=10090 GN=Stx7 PE=1 SV=3	O70439	Stx7	30 kDa	0.043	KO Female NT high, WT Female NT low	5	8	9	2	3	2
88	Lysosome-associated membrane glycoprotein 2 OS=Mus musculus (Mouse) OX=10090 GN=Lamp2 PE=1 SV=2	P17047	Lamp2	46 kDa	0.00045	KO Female NT high, WT Female NT low	4	4	5	0	1	0
89	Dipeptidyl peptidase 1 OS=Mus musculus (Mouse) OX=10090 GN=Ctsc PE=1 SV=1	P97821	Ctsc	52 kDa	0.016	KO Female NT high, WT Female NT low	7	5	3	0	0	0
90	Cluster of Chitinase-like protein 3 OS=Mus musculus (Mouse) OX=10090 GN=Chil3 PE=1 SV=2 (O35744)	O35744 [2]	Chil3	44 kDa	0.0083	KO Female NT high, WT Female NT low	3	6	4	1	1	0
91	Kallikrein-1 OS=Mus musculus (Mouse) OX=10090 GN=Klk1 PE=1 SV=3	P15947	Klk1	29 kDa	0.0061	KO Female NT high, WT Female NT low	4	5	6	1	1	1
92	Proteasome subunit alpha type-3 OS=Mus musculus (Mouse) OX=10090 GN=Psma3 PE=1 SV=3	O70435	Psma3	28 kDa	0.033	KO Female NT high, WT Female NT low	4	3	4	0	1	3

93	Actin-related protein 2/3 complex subunit 5 OS=Mus musculus (Mouse) OX=10090 GN=Arpc5 PE=1 SV=3	Q9CPW4	Arpc5	16 kDa	0.022	KO Female NT high, WT Female NT low	4	4	6	3	1	1
94	Ragulator complex protein LAMTOR5 OS=Mus musculus (Mouse) OX=10090 GN=Lamtor5 PE=1 SV=1	Q9D1L9	Lamtor5	10 kDa	0.0037	KO Female NT high, WT Female NT low	6	5	5	3	2	1
95	Di-N-acetylchitobiase OS=Mus musculus (Mouse) OX=10090 GN=Ctbs PE=1 SV=2	Q8R242	Ctbs	41 kDa	0.0065	KO Female NT high, WT Female NT low	9	6	8	0	0	0
96	Plasma alpha-L-fucosidase OS=Mus musculus (Mouse) OX=10090 GN=Fuca2 PE=1 SV=1	Q99KR8	Fuca2	54 kDa	0.035	KO Female NT high, WT Female NT low	9	5	3	0	0	0
97	Beta-hexosaminidase subunit alpha OS=Mus musculus (Mouse) OX=10090 GN=Hexa PE=1 SV=2	P29416	Hexa	61 kDa	0.003	KO Female NT high, WT Female NT low	5	4	5	0	0	0
98	LIM and SH3 domain protein 1 OS=Mus musculus (Mouse) OX=10090 GN=Lasp1 PE=1 SV=1	Q61792	Lasp1	30 kDa	0.023	KO Female NT high, WT Female NT low	3	5	5	1	1	2
99	Epididymis-specific alpha-mannosidase OS=Mus musculus (Mouse) OX=10090 GN=Man2b2 PE=1 SV=2	O54782	Man2b2	116 kDa	0.011	KO Female NT high, WT Female NT low	5	3	6	0	0	0
100	Mitochondrial import inner membrane translocase subunit TIM44 OS=Mus musculus (Mouse) OX=10090 GN=Timm44 PE=1 SV=2	O35857	Timm44	51 kDa	0.026	KO Female NT high, WT Female NT low	2	2	2	1	1	0
101	Galectin-3-binding protein OS=Mus musculus (Mouse) OX=10090 GN=Lgals3bp PE=1 SV=1	Q07797	Lgals3bp	64 kDa	0.003	KO Female NT high, WT Female NT low	5	4	6	0	0	0

102	Ras GTPase-activating protein-binding protein 1 OS=Mus musculus (Mouse) OX=10090 GN=G3bp1 PE=1 SV=1	P97855	G3bp1	52 kDa	0.0075	KO Female NT high, WT Female NT low	2	2	2	2	1	2
103	Transthyretin OS=Mus musculus (Mouse) OX=10090 GN=Ttr PE=1 SV=1	P07309	Ttr	16 kDa	0.00078	KO Female NT high, WT Female NT low	3	3	3	1	1	1
104	Tight junction protein ZO-1 OS=Mus musculus (Mouse) OX=10090 GN=Tjp1 PE=1 SV=2	P39447	Tjp1	195 kDa	0.00039	KO Female NT high, WT Female NT low	2	4	4	0	0	0
105	Transmembrane glycoprotein NMB OS=Mus musculus (Mouse) OX=10090 GN=Gpnmb PE=1 SV=2	Q99P91	Gpnmb	64 kDa	0.039	KO Female NT high, WT Female NT low	2	1	2	0	0	0
106	Osteoclast-stimulating factor 1 OS=Mus musculus (Mouse) OX=10090 GN=Ostf1 PE=1 SV=2	Q62422	Ostf1	24 kDa	0.013	KO Female NT high, WT Female NT low	4	3	3	2	2	2
107	Putative phospholipase B-like 2 OS=Mus musculus (Mouse) OX=10090 GN=Plbd2 PE=1 SV=2	Q3TCN2	Plbd2	66 kDa	0.023	KO Female NT high, WT Female NT low	5	5	2	0	0	0
108	ATPase GET3 OS=Mus musculus (Mouse) OX=10090 GN=Get3 PE=1 SV=2	O54984	Get3	39 kDa	0.0048	KO Female NT high, WT Female NT low	3	2	3	0	1	1
109	Tissue alpha-L-fucosidase OS=Mus musculus (Mouse) OX=10090 GN=Fuca1 PE=1 SV=1	Q99LJ1	Fuca1	52 kDa	0.038	KO Female NT high, WT Female NT low	6	2	3	0	0	0
110	Heterogeneous nuclear ribonucleoprotein A0 OS=Mus musculus (Mouse) OX=10090 GN=Hnrnpa0 PE=1 SV=1	Q9CX86	Hnrnpa0	31 kDa	0.023	KO Female NT high, WT Female NT low	4	3	4	1	1	3

111	Ubiquitin carboxyl-terminal hydrolase isozyme L1 OS=Mus musculus (Mouse) OX=10090 GN=Uchl1 PE=1 SV=1	Q9R0P9	Uchl1	25 kDa	0.047	KO Female NT high, WT Female NT low	2	3	4	1	0	1
112	RNA-binding protein 3 OS=Mus musculus (Mouse) OX=10090 GN=Rbm3 PE=1 SV=1	O89086	Rbm3	17 kDa	0.016	KO Female NT high, WT Female NT low	3	4	5	1	1	1
113	Protein Niban 1 OS=Mus musculus (Mouse) OX=10090 GN=Niban1 PE=1 SV=2	Q3UW53	Niban1	103 kDa	0.0023	KO Female NT high, WT Female NT low	4	2	3	0	0	0
114	Calponin-2 OS=Mus musculus (Mouse) OX=10090 GN=Cnn2 PE=1 SV=1	Q08093	Cnn2	33 kDa	0.026	KO Female NT high, WT Female NT low	2	1	3	0	0	0
115	Signal transducing adapter molecule 1 OS=Mus musculus (Mouse) OX=10090 GN=Stam PE=1 SV=3	P70297	Stam	60 kDa	0.0075	KO Female NT high, WT Female NT low	1	1	1	1	0	0
116	Cluster of GTP-binding protein SAR1b OS=Mus musculus (Mouse) OX=10090 GN=Sar1b PE=1 SV=1 (Q9CQC9)	Q9CQC9 [2]	Sar1b	22 kDa	0.024	KO Female NT high, WT Female NT low	3	1	2	0	1	1
117	Arylsulfatase A OS=Mus musculus (Mouse) OX=10090 GN=Arsa PE=1 SV=2	P50428	Arsa	54 kDa	0.017	KO Female NT high, WT Female NT low	4	5	7	0	0	0
118	Epidermal growth factor receptor substrate 15-like 1 OS=Mus musculus (Mouse) OX=10090 GN=Eps15l1 PE=1 SV=3	Q60902	Eps15l1	99 kDa	0.0075	KO Female NT high, WT Female NT low	3	6	6	0	0	0
119	Retinol-binding protein 1 OS=Mus musculus (Mouse) OX=10090 GN=Rbp1 PE=1 SV=2	Q00915	Rbp1	16 kDa	0.039	KO Female NT high, WT Female NT low	3	4	1	0	0	0

120	Ras GTPase-activating-like protein IQGAP2 OS=Mus musculus (Mouse) OX=10090 GN=Iqgap2 PE=1 SV=2	Q3UQ44	Iqgap2	181 kDa	0.016	KO Female NT high, WT Female NT low	1	2	1	0	0	0
121	SH3 domain-binding glutamic acid-rich-like protein 3 OS=Mus musculus (Mouse) OX=10090 GN=Sh3bgrl3 PE=1 SV=1	Q91VW3	Sh3bgrl3	10 kDa	0.016	KO Female NT high, WT Female NT low	2	5	3	0	0	0
122	Ragulator complex protein LAMTOR3 OS=Mus musculus (Mouse) OX=10090 GN=Lamtor3 PE=1 SV=1	O88653	Lamtor3	14 kDa	0.0053	KO Female NT high, WT Female NT low	3	3	2	0	0	0
123	Prefoldin subunit 2 OS=Mus musculus (Mouse) OX=10090 GN=Pfdn2 PE=1 SV=2	O70591	Pfdn2	17 kDa	0.033	KO Female NT high, WT Female NT low	2	2	4	1	0	0
124	H-2 class II histocompatibility antigen gamma chain OS=Mus musculus (Mouse) OX=10090 GN=Cd74 PE=1 SV=3	P04441	Cd74	32 kDa	0.031	KO Female NT high, WT Female NT low	1	1	2	0	0	0
125	Disks large homolog 1 OS=Mus musculus (Mouse) OX=10090 GN=Dlg1 PE=1 SV=1	Q811D0	Dlg1	100 kDa	0.016	KO Female NT high, WT Female NT low	2	4	5	0	0	0
126	NADP-dependent malic enzyme, mitochondrial OS=Mus musculus (Mouse) OX=10090 GN=Me3 PE=1 SV=2	Q8BMF3	Me3	67 kDa	0.0075	KO Female NT high, WT Female NT low	2	2	2	0	0	1
127	CD63 antigen OS=Mus musculus (Mouse) OX=10090 GN=Cd63 PE=1 SV=2	P41731	Cd63	26 kDa	0.0075	KO Female NT high, WT Female NT low	2	2	1	0	0	0
128	Eukaryotic translation initiation factor 4H OS=Mus musculus (Mouse) OX=10090 GN=Eif4h PE=1 SV=3	Q9WUK2	Eif4h	27 kDa	0.025	KO Female NT high, WT Female NT low	1	3	3	0	0	0

129	Eukaryotic translation initiation factor 5 OS=Mus musculus (Mouse) OX=10090 GN=Eif5 PE=1 SV=1	P59325	Eif5	49 kDa	0.0078	KO Female NT high, WT Female NT low	2	3	3	1	0	0
130	Pulmonary surfactant-associated protein B OS=Mus musculus (Mouse) OX=10090 GN=Sftpb PE=1 SV=1	P50405	Sftpb	42 kDa	0.0065	KO Female NT high, WT Female NT low	2	3	2	0	0	0
131	Guanylate kinase OS=Mus musculus (Mouse) OX=10090 GN=Guk1 PE=1 SV=2	Q64520	Guk1	22 kDa	0.025	KO Female NT high, WT Female NT low	4	3	2	1	1	0
132	Apoptosis-associated speck-like protein containing a CARD OS=Mus musculus (Mouse) OX=10090 GN=Pycard PE=1 SV=1	Q9EPB4	Pycard	21 kDa	0.016	KO Female NT high, WT Female NT low	2	2	5	0	0	0
133	Nidogen-1 OS=Mus musculus (Mouse) OX=10090 GN=Nid1 PE=1 SV=2	P10493	Nid1	137 kDa	0.016	KO Female NT high, WT Female NT low	3	2	2	0	1	0
134	Tax1-binding protein 3 OS=Mus musculus (Mouse) OX=10090 GN=Tax1bp3 PE=1 SV=1	Q9DBG9	Tax1bp3	14 kDa	0.0075	KO Female NT high, WT Female NT low	2	2	2	1	0	0
135	Ferritin light chain 1 OS=Mus musculus (Mouse) OX=10090 GN=Ftl1 PE=1 SV=2	P29391	Ftl1	21 kDa	0.0013	KO Female NT high, WT Female NT low	3	3	3	0	1	0
136	Complement factor I OS=Mus musculus (Mouse) OX=10090 GN=Cfi PE=1 SV=3	Q61129	Cfi	67 kDa	0.024	KO Female NT high, WT Female NT low	2	4	4	1	0	0
137	Glycosylated lysosomal membrane protein OS=Mus musculus (Mouse) OX=10090 GN=Glmp PE=1 SV=1	Q9JHJ3	Glmp	44 kDa	0.0022	KO Female NT high, WT Female NT low	2	2	2	0	0	0
138	Splicing factor 3B subunit 1 OS=Mus musculus (Mouse) OX=10090 GN=Sf3b1 PE=1 SV=1	Q99NB9	Sf3b1	146 kDa	0.016	KO Female NT high, WT	1	2	1	0	0	0

						Female NT low						
139	Epidermal growth factor receptor substrate 15 OS=Mus musculus (Mouse) OX=10090 GN=Eps15 PE=1 SV=1	P42567	Eps15	98 kDa	0.0022	KO Female NT high, WT Female NT low	2	2	3	0	0	0
140	Protein SGT1 homolog OS=Mus musculus (Mouse) OX=10090 GN=Sugt1 PE=1 SV=3	Q9CX34	Sugt1	38 kDa	0.024	KO Female NT high, WT Female NT low	4	2	5	0	0	1
141	Chloride intracellular channel protein 6 OS=Mus musculus (Mouse) OX=10090 GN=Clic6 PE=1 SV=1	Q8BHB9	Clic6	63 kDa	0.00056	KO Female NT high, WT Female NT low	2	2	2	0	0	0
142	STE20/SPS1-related proline-alanine-rich protein kinase OS=Mus musculus (Mouse) OX=10090 GN=Stk39 PE=1 SV=1	Q9Z1W9	Stk39	60 kDa	0.0022	KO Female NT high, WT Female NT low	2	2	3	0	0	0
143	Small nuclear ribonucleoprotein Sm D3 OS=Mus musculus (Mouse) OX=10090 GN=Snrpd3 PE=1 SV=1	P62320	Snrpd3	14 kDa	0.024	KO Female NT high, WT Female NT low	1	1	2	1	1	0
144	Sialate O-acetylesterase OS=Mus musculus (Mouse) OX=10090 GN=Siae PE=1 SV=3	P70665	Siae	61 kDa	0.026	KO Female NT high, WT Female NT low	1	1	2	0	0	0
145	Serine/threonine-protein phosphatase 6 regulatory subunit 3 OS=Mus musculus (Mouse) OX=10090 GN=Ppp6r3 PE=1 SV=1	Q922D4	Ppp6r3	95 kDa	0.0075	KO Female NT high, WT Female NT low	1	2	2	0	0	0
146	Syntaxin-12 OS=Mus musculus (Mouse) OX=10090 GN=Stx12 PE=1 SV=1	Q9ER00	Stx12	31 kDa	0.0075	KO Female NT high, WT Female NT low	1	2	2	0	0	0
147	Regulator of microtubule dynamics protein 1 OS=Mus musculus (Mouse) OX=10090 GN=Rmdn1 PE=1 SV=2	Q9DCV4	Rmdn1	35 kDa	0.047	KO Female NT high, WT Female NT low	2	2	1	1	0	0

148	Phosphoribosyl pyrophosphate synthase-associated protein 1 OS=Mus musculus (Mouse) OX=10090 GN=Prpsap1 PE=1 SV=1	Q9D0M1	Prpsap1	39 kDa	0.047	KO Female NT high, WT Female NT low	0	1	0	0	0	0
149	Methyl-CpG-binding protein 2 OS=Mus musculus (Mouse) OX=10090 GN=Mecp2 PE=1 SV=1	Q9Z2D6	Mecp2	52 kDa	0.047	KO Female NT high, WT Female NT low	1	2	2	1	0	0
150	Osteopontin OS=Mus musculus (Mouse) OX=10090 GN=Spp1 PE=1 SV=1	P10923	Spp1	32 kDa	0.0075	KO Female NT high, WT Female NT low	1	1	1	0	0	0

List of proteins reduced in kidney of 4-month-old male *Neu1$^{\Delta Ex3}$* mice.							Exclusive unique peptide count					
#	Identified Proteins (1841)	Accession Number	Alternate ID	MW	T-Test (p-value): ($p < 0.05$)	Quantitative Profile	KO1	KO2	KO3	WT1	WT2	WT3
1	60 kDa heat shock protein, mitochondrial OS=Mus musculus (Mouse) OX=10090 GN=Hspd1 PE=1 SV=1	P63038	Hspd1	61 kDa	0.00049	KO Male NT low, WT Male NT high	65	71	57	85	84	81
2	Dihydrolipoyl dehydrogenase, mitochondrial OS=Mus musculus (Mouse) OX=10090 GN=Dld PE=1 SV=2	O08749	Dld	54 kDa	0.024	KO Male NT low, WT Male NT high	39	39	39	46	60	50
3	Kynurenine/alpha-aminoadipate aminotransferase, mitochondrial OS=Mus musculus (Mouse) OX=10090 GN=Aadat PE=1 SV=1	Q9WVM8	Aadat	48 kDa	0.048	KO Male NT low, WT Male NT high	8	10	9	15	12	20
4	Cluster of Alpha-1-antitrypsin 1-2 OS=Mus musculus (Mouse) OX=10090 GN=Serpina1b PE=1 SV=2 (P22599)	P22599 [5]	Serpina1b	46 kDa	0.00094	KO Male NT low, WT Male NT high	6	7	3	39	15	39
5	Apolipoprotein A-I OS=Mus musculus (Mouse) OX=10090 GN=Apoa1 PE=1 SV=2	Q00623	Apoa1	31 kDa	0.048	KO Male NT low, WT Male NT high	17	29	23	33	34	31
6	Meprin A subunit alpha OS=Mus musculus (Mouse) OX=10090 GN=Mep1a PE=1 SV=4	P28825	Mep1a	84 kDa	0.021	KO Male NT low, WT Male NT high	21	18	18	29	29	32
7	Probable D-lactate dehydrogenase, mitochondrial OS=Mus musculus (Mouse) OX=10090 GN=Ldhd PE=1 SV=1	Q7TNG8	Ldhd	52 kDa	0.042	KO Male NT low, WT Male NT high	25	36	20	41	41	53
8	Cytochrome c oxidase subunit 5A, mitochondrial OS=Mus musculus	P12787	Cox5a	16 kDa	0.025	KO Male NT low, WT Male NT high	18	17	18	35	38	23

	(Mouse) OX=10090 GN=Cox5a PE=1 SV=2											
9	Endoribonuclease LACTB2 OS=Mus musculus (Mouse) OX=10090 GN=Lactb2 PE=1 SV=1	Q99KR3	Lactb2	33 kDa	0.02	KO Male NT low, WT Male NT high	19	20	20	25	27	24
10	Cluster of Cytochrome c, somatic OS=Mus musculus (Mouse) OX=10090 GN=Cycs PE=1 SV=2 (P62897)	P62897 [2]	Cycs	12 kDa	0.015	KO Male NT low, WT Male NT high	18	18	18	23	26	23
11	ATP synthase subunit d, mitochondrial OS=Mus musculus (Mouse) OX=10090 GN=Atp5pd PE=1 SV=3	Q9DCX2	Atp5pd	19 kDa	0.016	KO Male NT low, WT Male NT high	13	11	14	27	40	21
12	Homogentisate 1,2-dioxygenase OS=Mus musculus (Mouse) OX=10090 GN=Hgd PE=1 SV=2	O09173	Hgd	50 kDa	0.014	KO Male NT low, WT Male NT high	20	23	24	30	35	31
13	NADP-dependent malic enzyme OS=Mus musculus (Mouse) OX=10090 GN=Me1 PE=1 SV=2	P06801	Me1	64 kDa	0.0041	KO Male NT low, WT Male NT high	18	22	15	34	32	34
14	Cytochrome b-c1 complex subunit 6, mitochondrial OS=Mus musculus (Mouse) OX=10090 GN=Uqcrh PE=1 SV=2	P99028	Uqcrh	10 kDa	0.0074	KO Male NT low, WT Male NT high	9	10	10	13	19	16
15	Alcohol dehydrogenase 1 OS=Mus musculus (Mouse) OX=10090 GN=Adh1 PE=1 SV=2	P00329	Adh1	40 kDa	0.033	KO Male NT low, WT Male NT high	16	21	15	25	23	29
16	Alpha-2-macroglobulin receptor-associated protein OS=Mus musculus (Mouse) OX=10090 GN=Lrpap1 PE=1 SV=1	P55302	Lrpap1	42 kDa	0.042	KO Male NT low, WT Male NT high	7	11	5	26	32	17

17	Cluster of Histone H1.3 OS=Mus musculus (Mouse) OX=10090 GN=H1-3 PE=1 SV=2 (P43277)	P43277 [3]	H1-3	22 kDa	0.043	KO Male NT low, WT Male NT high	6	0	4	14	9	5
18	Peroxisomal carnitine O-octanoyltransferase OS=Mus musculus (Mouse) OX=10090 GN=Crot PE=1 SV=1	Q9DC50	Crot	70 kDa	0.0024	KO Male NT low, WT Male NT high	11	15	14	26	26	31
19	Aminoacylase-1 OS=Mus musculus (Mouse) OX=10090 GN=Acy1 PE=1 SV=1	Q99JW2	Acy1	46 kDa	0.014	KO Male NT low, WT Male NT high	6	10	5	17	17	14
20	Glutathione hydrolase 1 proenzyme OS=Mus musculus (Mouse) OX=10090 GN=Ggt1 PE=1 SV=1	Q60928	Ggt1	62 kDa	0.0089	KO Male NT low, WT Male NT high	9	7	12	14	15	20
21	Anionic trypsin-2 OS=Mus musculus (Mouse) OX=10090 GN=Prss2 PE=1 SV=1	P07146	Prss2	26 kDa	0.017	KO Male NT low, WT Male NT high	8	8	7	16	17	16
22	Meprin A subunit beta OS=Mus musculus (Mouse) OX=10090 GN=Mep1b PE=1 SV=2	Q61847	Mep1b	80 kDa	0.0099	KO Male NT low, WT Male NT high	10	10	8	18	15	15
23	Cluster of Cysteine sulfinic acid decarboxylase OS=Mus musculus (Mouse) OX=10090 GN=Csad PE=1 SV=1 (Q9DBE0)	Q9DBE0 [2]	Csad	55 kDa	0.027	KO Male NT low, WT Male NT high	8	12	10	18	14	18
24	Cytidine deaminase OS=Mus musculus (Mouse) OX=10090 GN=Cda PE=1 SV=2	P56389	Cda	16 kDa	0.043	KO Male NT low, WT Male NT high	8	12	10	15	22	16
25	Acyl-coenzyme A synthetase ACSM3, mitochondrial OS=Mus musculus (Mouse) OX=10090 GN=Acsm3 PE=1 SV=2	Q3UNX5	Acsm3	66 kDa	0.00021	KO Male NT low, WT Male NT high	7	12	8	38	41	37

26	Nascent polypeptide-associated complex subunit alpha, muscle-specific form OS=Mus musculus (Mouse) OX=10090 GN=Naca PE=1 SV=2	P70670	Naca	220 kDa	0.0031	KO Male NT low, WT Male NT high	4	4	5	6	6	6
27	Serine/arginine-rich splicing factor 2 OS=Mus musculus (Mouse) OX=10090 GN=Srsf2 PE=1 SV=4	Q62093	Srsf2	25 kDa	0.036	KO Male NT low, WT Male NT high	2	3	4	6	7	2
28	Cytochrome c oxidase subunit 6B1 OS=Mus musculus (Mouse) OX=10090 GN=Cox6b1 PE=1 SV=2	P56391	Cox6b1	10 kDa	0.017	KO Male NT low, WT Male NT high	3	4	6	7	8	10
29	Peroxisomal acyl-coenzyme A oxidase 2 OS=Mus musculus (Mouse) OX=10090 GN=Acox2 PE=1 SV=2	Q9QXD1	Acox2	77 kDa	0.046	KO Male NT low, WT Male NT high	5	4	2	11	6	12
30	Electrogenic sodium bicarbonate cotransporter 1 OS=Mus musculus (Mouse) OX=10090 GN=Slc4a4 PE=1 SV=2	O88343	Slc4a4	121 kDa	0.02	KO Male NT low, WT Male NT high	6	8	3	11	12	10
31	Nucleophosmin OS=Mus musculus (Mouse) OX=10090 GN=Npm1 PE=1 SV=1	Q61937	Npm1	33 kDa	0.049	KO Male NT low, WT Male NT high	4	4	7	11	10	8
32	Protein 4.1 OS=Mus musculus (Mouse) OX=10090 GN=Epb41 PE=1 SV=2	P48193	Epb41	96 kDa	0.036	KO Male NT low, WT Male NT high	5	3	2	10	14	6
33	Adenylate kinase 4, mitochondrial OS=Mus musculus (Mouse) OX=10090 GN=Ak4 PE=1 SV=1	Q9WUR9	Ak4	25 kDa	0.026	KO Male NT low, WT Male NT high	2	5	1	11	7	8
34	3-hydroxyanthranilate 3,4-dioxygenase OS=Mus musculus (Mouse) OX=10090 GN=Haao PE=1 SV=1	Q78JT3	Haao	33 kDa	0.0088	KO Male NT low, WT Male NT high	7	6	1	15	18	13

35	Cluster of High mobility group protein B1 OS=Mus musculus (Mouse) OX=10090 GN=Hmgb1 PE=1 SV=2 (P63158)	P63158 [2]	Hmgb1	25 kDa	0.029	KO Male NT low, WT Male NT high	3	1	4	9	11	7
36	Plasminogen activator inhibitor 1 RNA-binding protein OS=Mus musculus (Mouse) OX=10090 GN=Serbp1 PE=1 SV=2	Q9CY58	Serbp1	45 kDa	0.048	KO Male NT low, WT Male NT high	0	0	0	16	24	7
37	Peroxisomal acyl-coenzyme A oxidase 3 OS=Mus musculus (Mouse) OX=10090 GN=Acox3 PE=1 SV=2	Q9EPL9	Acox3	78 kDa	0.032	KO Male NT low, WT Male NT high	6	8	1	16	12	22
38	Cytochrome b-c1 complex subunit Rieske, mitochondrial OS=Mus musculus (Mouse) OX=10090 GN=Uqcrfs1 PE=1 SV=1	Q9CR68	Uqcrfs1	29 kDa	0.0022	KO Male NT low, WT Male NT high	3	4	2	10	9	8
39	Cluster of Y-box-binding protein 1 OS=Mus musculus (Mouse) OX=10090 GN=Ybx1 PE=1 SV=3 (P62960)	P62960 [2]	Ybx1	36 kDa	0.021	KO Male NT low, WT Male NT high	7	2	7	8	4	8
40	Cluster of Ubiquitin-40S ribosomal protein S27a OS=Mus musculus (Mouse) OX=10090 GN=Rps27a PE=1 SV=2 (P62983)	P62983 [3]	Rps27a	18 kDa	0.0075	KO Male NT low, WT Male NT high	1	1	2	5	1	3
41	Nicotinate-nucleotide pyrophosphorylase [carboxylating] OS=Mus musculus (Mouse) OX=10090 GN=Qprt PE=1 SV=1	Q91X91	Qprt	32 kDa	0.05	KO Male NT low, WT Male NT high	4	5	7	11	7	7
42	Prohibitin-2 OS=Mus musculus (Mouse) OX=10090 GN=Phb2 PE=1 SV=1	O35129	Phb2	33 kDa	0.011	KO Male NT low, WT Male NT high	1	3	1	6	6	8
43	Cytochrome c oxidase subunit 7A2, mitochondrial OS=Mus musculus (Mouse) OX=10090 GN=Cox7a2 PE=1 SV=2	P48771	Cox7a2	9 kDa	0.04	KO Male NT low, WT Male NT high	4	4	4	6	5	5

44	Deaminated glutathione amidase OS=Mus musculus (Mouse) OX=10090 GN=Nit1 PE=1 SV=2	Q8VDK1	Nit1	36 kDa	0.0072	KO Male NT low, WT Male NT high	5	6	2	10	10	7
45	Enoyl-CoA delta isomerase 3, peroxisomal OS=Mus musculus (Mouse) OX=10090 GN=Eci3 PE=1 SV=1	Q78JN3	Eci3	35 kDa	0.0033	KO Male NT low, WT Male NT high	3	3	3	3	4	4
46	Glutaredoxin-related protein 5, mitochondrial OS=Mus musculus (Mouse) OX=10090 GN=Glrx5 PE=1 SV=2	Q80Y14	Glrx5	16 kDa	0.039	KO Male NT low, WT Male NT high	5	4	5	7	9	5
47	Acyl carrier protein, mitochondrial OS=Mus musculus (Mouse) OX=10090 GN=Ndufab1 PE=1 SV=1	Q9CR21	Ndufab1	17 kDa	0.015	KO Male NT low, WT Male NT high	2	3	3	10	11	7
48	Clusterin OS=Mus musculus (Mouse) OX=10090 GN=Clu PE=1 SV=1	Q06890	Clu	52 kDa	0.035	KO Male NT low, WT Male NT high	3	2	3	11	12	5
49	Ubiquinone biosynthesis protein COQ9, mitochondrial OS=Mus musculus (Mouse) OX=10090 GN=Coq9 PE=1 SV=1	Q8K1Z0	Coq9	35 kDa	0.016	KO Male NT low, WT Male NT high	4	3	4	8	6	10
50	Heat shock protein 105 kDa OS=Mus musculus (Mouse) OX=10090 GN=Hsph1 PE=1 SV=2	Q61699	Hsph1	96 kDa	0.015	KO Male NT low, WT Male NT high	0	2	2	5	5	6
51	NADH dehydrogenase [ubiquinone] 1 beta subcomplex subunit 10 OS=Mus musculus (Mouse) OX=10090 GN=Ndufb10 PE=1 SV=3	Q9DCS9	Ndufb10	21 kDa	0.025	KO Male NT low, WT Male NT high	4	2	4	5	5	6
52	Caprin-1 OS=Mus musculus (Mouse) OX=10090 GN=Caprin1 PE=1 SV=2	Q60865	Caprin1	78 kDa	0.042	KO Male NT low, WT Male NT high	2	5	3	8	10	6

53	EH domain-containing protein 4 OS=Mus musculus (Mouse) OX=10090 GN=Ehd4 PE=1 SV=1	Q9EQP2	Ehd4	61 kDa	0.047	KO Male NT low, WT Male NT high	1	2	1	3	1	2
54	NADH dehydrogenase [ubiquinone] iron-sulfur protein 4, mitochondrial OS=Mus musculus (Mouse) OX=10090 GN=Ndufs4 PE=1 SV=3	Q9CXZ1	Ndufs4	20 kDa	0.041	KO Male NT low, WT Male NT high	5	2	2	7	6	4
55	Cytochrome c oxidase subunit NDUFA4 OS=Mus musculus (Mouse) OX=10090 GN=Ndufa4 PE=1 SV=2	Q62425	Ndufa4	9 kDa	0.041	KO Male NT low, WT Male NT high	1	2	1	8	6	2
56	Band 4.1-like protein 1 OS=Mus musculus (Mouse) OX=10090 GN=Epb41l1 PE=1 SV=2	Q9Z2H5	Epb41l1	98 kDa	0.025	KO Male NT low, WT Male NT high	1	1	1	4	4	1
57	NADH dehydrogenase [ubiquinone] 1 subunit C2 OS=Mus musculus (Mouse) OX=10090 GN=Ndufc2 PE=1 SV=1	Q9CQ54	Ndufc2	14 kDa	0.014	KO Male NT low, WT Male NT high	2	0	2	4	6	4
58	Glycine cleavage system H protein, mitochondrial OS=Mus musculus (Mouse) OX=10090 GN=Gcsh PE=1 SV=2	Q91WK5	Gcsh	19 kDa	0.039	KO Male NT low, WT Male NT high	3	3	2	5	6	5
59	NADH dehydrogenase [ubiquinone] 1 alpha subcomplex subunit 5 OS=Mus musculus (Mouse) OX=10090 GN=Ndufa5 PE=1 SV=3	Q9CPP6	Ndufa5	13 kDa	0.045	KO Male NT low, WT Male NT high	1	1	1	6	7	3
60	Cytochrome b-c1 complex subunit 8 OS=Mus musculus (Mouse) OX=10090 GN=Uqcrq PE=1 SV=3	Q9CQ69	Uqcrq	10 kDa	0.047	KO Male NT low, WT Male NT high	2	2	2	2	3	4
61	Histone H1.0 OS=Mus musculus (Mouse) OX=10090 GN=H1-0 PE=2 SV=4	P10922	H1-0	21 kDa	0.016	KO Male NT low, WT Male NT high	1	0	3	5	4	5

62	NADH dehydrogenase [ubiquinone] iron-sulfur protein 5 OS=Mus musculus (Mouse) OX=10090 GN=Ndufs5 PE=1 SV=3	Q99LY9	Ndufs5	13 kDa	0.039	KO Male NT low, WT Male NT high	1	1	1	3	4	2
63	D-dopachrome decarboxylase OS=Mus musculus (Mouse) OX=10090 GN=Ddt PE=1 SV=3	O35215	Ddt	13 kDa	0.041	KO Male NT low, WT Male NT high	0	0	1	5	11	5
64	UV excision repair protein RAD23 homolog A OS=Mus musculus (Mouse) OX=10090 GN=Rad23a PE=1 SV=2	P54726	Rad23a	40 kDa	0.019	KO Male NT low, WT Male NT high	0	0	0	2	3	1
65	Eukaryotic translation initiation factor 3 subunit J-A OS=Mus musculus (Mouse) OX=10090 GN=Eif3j1 PE=2 SV=1	Q3UGC7 (+1)	Eif3j1	29 kDa	0.0075	KO Male NT low, WT Male NT high	1	2	2	5	6	4
66	Endoplasmic reticulum resident protein 29 OS=Mus musculus (Mouse) OX=10090 GN=Erp29 PE=1 SV=2	P57759	Erp29	29 kDa	0.00039	KO Male NT low, WT Male NT high	0	0	0	4	4	2
67	Signal transducing adapter molecule 1 OS=Mus musculus (Mouse) OX=10090 GN=Stam PE=1 SV=3	P70297	Stam	60 kDa	0.047	KO Male NT low, WT Male NT high	0	0	1	1	1	1
68	NADH dehydrogenase [ubiquinone] 1 alpha subcomplex subunit 2 OS=Mus musculus (Mouse) OX=10090 GN=Ndufa2 PE=1 SV=3	Q9CQ75	Ndufa2	11 kDa	0.047	KO Male NT low, WT Male NT high	1	1	1	3	2	2
69	Transcription factor BTF3 OS=Mus musculus (Mouse) OX=10090 GN=Btf3 PE=1 SV=3	Q64152	Btf3	22 kDa	0.0022	KO Male NT low, WT Male NT high	0	0	0	2	3	1
70	Acylphosphatase-2 OS=Mus musculus (Mouse) OX=10090 GN=Acyp2 PE=1 SV=2	P56375	Acyp2	12 kDa	0.0075	KO Male NT low, WT Male NT high	1	1	1	3	3	2

71	Mitochondrial fission 1 protein OS=Mus musculus (Mouse) OX=10090 GN=Fis1 PE=1 SV=1	Q9CQ92	Fis1	17 kDa	0.0075	KO Male NT low, WT Male NT high	1	1	1	2	3	2
72	ATP-dependent Clp protease ATP-binding subunit clpX-like, mitochondrial OS=Mus musculus (Mouse) OX=10090 GN=Clpx PE=1 SV=2	Q9JHS4	Clpx	69 kDa	0.047	KO Male NT low, WT Male NT high	0	1	0	2	1	2
73	Proteasome subunit beta type-6 OS=Mus musculus (Mouse) OX=10090 GN=Psmb6 PE=1 SV=3	Q60692	Psmb6	25 kDa	0.016	KO Male NT low, WT Male NT high	1	1	1	3	1	2
74	CDGSH iron-sulfur domain-containing protein 3, mitochondrial OS=Mus musculus (Mouse) OX=10090 GN=Cisd3 PE=1 SV=1	B1AR13	Cisd3	16 kDa	0.0075	KO Male NT low, WT Male NT high	0	0	0	2	1	2
75	NADH dehydrogenase [ubiquinone] 1 alpha subcomplex subunit 3 OS=Mus musculus (Mouse) OX=10090 GN=Ndufa3 PE=1 SV=1	Q9CQ91	Ndufa3	9 kDa	0.0075	KO Male NT low, WT Male NT high	0	0	0	1	2	2

List of proteins increased in kidney of 4-month-old male *Neu1*$^{\Delta Ex3}$ mice.							Exclusive unique peptide count					
#	Identified Proteins (1841)	Accession Number	Alternate ID	MW	T-Test (p-value): ($p < 0.05$)	Quantitative Profile	KO1	KO2	KO3	WT1	WT2	WT3
1	Albumin OS=Mus musculus (Mouse) OX=10090 GN=Alb PE=1 SV=3	P07724	Alb	69 kDa	0.043	KO Male NT high, WT Male NT low	226	209	238	186	181	210
2	Cluster of Actin, cytoplasmic 2 OS=Mus musculus (Mouse) OX=10090 GN=Actg1 PE=1 SV=1 (P63260)	P63260 [7]	Actg1	42 kDa	0.022	KO Male NT high, WT Male NT low	80	130	76	61	42	71
3	Spectrin alpha chain, non-erythrocytic 1 OS=Mus musculus (Mouse) OX=10090 GN=Sptan1 PE=1 SV=4	P16546	Sptan1	285 kDa	0.012	KO Male NT high, WT Male NT low	119	116	138	93	98	111
4	Myosin-9 OS=Mus musculus (Mouse) OX=10090 GN=Myh9 PE=1 SV=4	Q8VDD5	Myh9	226 kDa	0.017	KO Male NT high, WT Male NT low	87	89	104	82	76	70
5	Spectrin beta chain, non-erythrocytic 1 OS=Mus musculus (Mouse) OX=10090 GN=Sptbn1 PE=1 SV=2	Q62261	Sptbn1	274 kDa	0.036	KO Male NT high, WT Male NT low	71	74	89	70	59	76
6	Cluster of Lysozyme C-2 OS=Mus musculus (Mouse) OX=10090 GN=Lyz2 PE=1 SV=2 (P08905)	P08905 [2]	Lyz2	17 kDa	< 0.00010	KO Male NT high, WT Male NT low	68	63	61	1	2	2
7	Cluster of Tubulin alpha-1C chain OS=Mus musculus (Mouse) OX=10090 GN=Tuba1c PE=1 SV=1 (P68373)	P68373 [5]	Tuba1c	50 kDa	0.031	KO Male NT high, WT Male NT low	58	51	58	28	35	39
8	Cluster of Alpha-actinin-4 OS=Mus musculus (Mouse) OX=10090 GN=Actn4 PE=1 SV=1 (P57780)	P57780 [3]	Actn4	105 kDa	0.012	KO Male NT high, WT Male NT low	62	58	68	40	38	45
9	Filamin-B OS=Mus musculus (Mouse) OX=10090 GN=Flnb PE=1 SV=3	Q80X90	Flnb	278 kDa	0.018	KO Male NT high, WT Male NT low	42	38	49	31	23	24

10	Cluster of Ras-related protein Rab-1A OS=Mus musculus (Mouse) OX=10090 GN=Rab1A PE=1 SV=3 (P62821)	P62821 [21]	Rab1A	23 kDa	0.028	KO Male NT high, WT Male NT low	61	59	67	33	11	44
11	Pyruvate kinase PKM OS=Mus musculus (Mouse) OX=10090 GN=Pkm PE=1 SV=4	P52480	Pkm	58 kDa	0.0029	KO Male NT high, WT Male NT low	57	51	56	30	22	41
12	Elongation factor 2 OS=Mus musculus (Mouse) OX=10090 GN=Eef2 PE=1 SV=2	P58252	Eef2	95 kDa	0.045	KO Male NT high, WT Male NT low	37	40	42	27	14	33
13	Cytoplasmic dynein 1 heavy chain 1 OS=Mus musculus (Mouse) OX=10090 GN=Dync1h1 PE=1 SV=2	Q9JHU4	Dync1h1	532 kDa	0.0028	KO Male NT high, WT Male NT low	36	40	45	9	0	16
14	Talin-1 OS=Mus musculus (Mouse) OX=10090 GN=Tln1 PE=1 SV=2	P26039	Tln1	270 kDa	0.027	KO Male NT high, WT Male NT low	26	30	33	20	15	21
15	Glucose-6-phosphate isomerase OS=Mus musculus (Mouse) OX=10090 GN=Gpi PE=1 SV=4	P06745	Gpi	63 kDa	0.022	KO Male NT high, WT Male NT low	35	37	36	17	10	29
16	Moesin OS=Mus musculus (Mouse) OX=10090 GN=Msn PE=1 SV=3	P26041	Msn	68 kDa	0.04	KO Male NT high, WT Male NT low	23	20	25	7	10	18
17	Filamin-A OS=Mus musculus (Mouse) OX=10090 GN=Flna PE=1 SV=5	Q8BTM8	Flna	281 kDa	0.039	KO Male NT high, WT Male NT low	43	37	68	20	21	14
18	V-type proton ATPase catalytic subunit A OS=Mus musculus (Mouse) OX=10090 GN=Atp6v1a PE=1 SV=2	P50516	Atp6v1a	68 kDa	0.047	KO Male NT high, WT Male NT low	55	55	52	23	18	41
19	Vinculin OS=Mus musculus (Mouse) OX=10090 GN=Vcl PE=1 SV=4	Q64727	Vcl	117 kDa	0.033	KO Male NT high, WT Male NT low	30	30	44	22	14	22
20	Cathepsin D OS=Mus musculus (Mouse) OX=10090 GN=Ctsd PE=1 SV=1	P18242	Ctsd	45 kDa	0.0015	KO Male NT high, WT Male NT low	39	28	42	6	6	11

21	Progranulin OS=Mus musculus (Mouse) OX=10090 GN=Grn PE=1 SV=2	P28798	Grn	63 kDa	0.0035	KO Male NT high, WT Male NT low	45	41	48	18	27	11
22	V-type proton ATPase subunit B, brain isoform OS=Mus musculus (Mouse) OX=10090 GN=Atp6v1b2 PE=1 SV=1	P62814	Atp6v1b2	57 kDa	0.049	KO Male NT high, WT Male NT low	43	36	30	20	19	34
23	Hydroxyacid oxidase 2 OS=Mus musculus (Mouse) OX=10090 GN=Hao2 PE=1 SV=1	Q9NYQ2	Hao2	39 kDa	0.0064	KO Male NT high, WT Male NT low	25	23	25	10	14	16
24	Cathepsin B OS=Mus musculus (Mouse) OX=10090 GN=Ctsb PE=1 SV=2	P10605	Ctsb	37 kDa	0.00071	KO Male NT high, WT Male NT low	39	35	40	8	9	13
25	Cluster of Heat shock 70 kDa protein 1A OS=Mus musculus (Mouse) OX=10090 GN=Hspa1a PE=1 SV=2 (Q61696)	Q61696 [2]	Hspa1a	70 kDa	0.038	KO Male NT high, WT Male NT low	11	13	11	10	4	9
26	Complement C3 OS=Mus musculus (Mouse) OX=10090 GN=C3 PE=1 SV=3	P01027	C3	186 kDa	0.0031	KO Male NT high, WT Male NT low	27	30	26	18	11	7
27	Heterogeneous nuclear ribonucleoprotein A3 OS=Mus musculus (Mouse) OX=10090 GN=Hnrnpa3 PE=1 SV=1	Q8BG05	Hnrnpa3	40 kDa	0.043	KO Male NT high, WT Male NT low	29	26	25	18	18	17
28	Cofilin-1 OS=Mus musculus (Mouse) OX=10090 GN=Cfl1 PE=1 SV=3	P18760	Cfl1	19 kDa	0.019	KO Male NT high, WT Male NT low	15	19	22	15	17	13
29	Uromodulin OS=Mus musculus (Mouse) OX=10090 GN=Umod PE=1 SV=1	Q91X17	Umod	71 kDa	0.0028	KO Male NT high, WT Male NT low	45	33	41	4	6	3
30	Heat shock 70 kDa protein 4 OS=Mus musculus (Mouse) OX=10090 GN=Hspa4 PE=1 SV=1	Q61316	Hspa4	94 kDa	0.0067	KO Male NT high, WT Male NT low	22	23	21	15	11	15
31	Ubiquitin-like modifier-activating enzyme 1 OS=Mus musculus (Mouse) OX=10090 GN=Uba1 PE=1 SV=1	Q02053	Uba1	118 kDa	0.019	KO Male NT high, WT Male NT low	30	30	27	12	6	19

32	Peroxiredoxin-2 OS=Mus musculus (Mouse) OX=10090 GN=Prdx2 PE=1 SV=3	Q61171	Prdx2	22 kDa	0.022	KO Male NT high, WT Male NT low	22	19	20	14	15	15
33	Acetyl-coenzyme A synthetase 2-like, mitochondrial OS=Mus musculus (Mouse) OX=10090 GN=Acss1 PE=1 SV=1	Q99NB1	Acss1	75 kDa	0.049	KO Male NT high, WT Male NT low	22	23	23	14	8	19
34	Cluster of Glutathione S-transferase A2 OS=Mus musculus (Mouse) OX=10090 GN=Gsta2 PE=1 SV=3 (P10648)	P10648 [2]	Gsta2	26 kDa	0.025	KO Male NT high, WT Male NT low	0	0	11	1	1	0
35	Cluster of Acyl-coenzyme A thioesterase 1 OS=Mus musculus (Mouse) OX=10090 GN=Acot1 PE=1 SV=1 (O55137)	O55137 [4]	Acot1	46 kDa	0.017	KO Male NT high, WT Male NT low	20	17	27	12	7	13
36	Beta-mannosidase OS=Mus musculus (Mouse) OX=10090 GN=Manba PE=1 SV=1	Q8K2I4	Manba	101 kDa	0.00069	KO Male NT high, WT Male NT low	37	30	36	0	2	0
37	14-3-3 protein theta OS=Mus musculus (Mouse) OX=10090 GN=Ywhaq PE=1 SV=1	P68254	Ywhaq	28 kDa	0.0061	KO Male NT high, WT Male NT low	12	13	10	7	6	10
38	Ganglioside GM2 activator OS=Mus musculus (Mouse) OX=10090 GN=Gm2a PE=1 SV=2	Q60648	Gm2a	21 kDa	0.0075	KO Male NT high, WT Male NT low	25	23	30	12	15	7
39	Gelsolin OS=Mus musculus (Mouse) OX=10090 GN=Gsn PE=1 SV=3	P13020	Gsn	86 kDa	0.019	KO Male NT high, WT Male NT low	22	17	21	12	10	13
40	Cluster of Carbonyl reductase [NADPH] 1 OS=Mus musculus (Mouse) OX=10090 GN=Cbr1 PE=1 SV=3 (P48758)	P48758 [2]	Cbr1	31 kDa	0.043	KO Male NT high, WT Male NT low	9	12	18	4	3	5
41	Plastin-3 OS=Mus musculus (Mouse) OX=10090 GN=Pls3 PE=1 SV=3	Q99K51	Pls3	71 kDa	0.0059	KO Male NT high, WT Male NT low	16	15	16	7	3	10
42	Plastin-2 OS=Mus musculus (Mouse) OX=10090 GN=Lcp1 PE=1 SV=4	Q61233	Lcp1	70 kDa	0.0012	KO Male NT high, WT Male NT low	24	19	26	3	0	4

43	Pro-cathepsin H OS=Mus musculus (Mouse) OX=10090 GN=Ctsh PE=1 SV=2	P49935	Ctsh	37 kDa	0.001	KO Male NT high, WT Male NT low	25	24	23	11	14	11
44	Annexin A5 OS=Mus musculus (Mouse) OX=10090 GN=Anxa5 PE=1 SV=1	P48036	Anxa5	36 kDa	0.017	KO Male NT high, WT Male NT low	20	23	23	15	13	15
45	Carbonic anhydrase 3 OS=Mus musculus (Mouse) OX=10090 GN=Ca3 PE=1 SV=3	P16015	Ca3	29 kDa	0.024	KO Male NT high, WT Male NT low	20	14	18	10	10	13
46	Adenylyl cyclase-associated protein 1 OS=Mus musculus (Mouse) OX=10090 GN=Cap1 PE=1 SV=4	P40124	Cap1	52 kDa	0.0083	KO Male NT high, WT Male NT low	21	19	19	10	8	12
47	Profilin-1 OS=Mus musculus (Mouse) OX=10090 GN=Pfn1 PE=1 SV=2	P62962	Pfn1	15 kDa	0.016	KO Male NT high, WT Male NT low	25	19	22	12	8	16
48	Unconventional myosin-VI OS=Mus musculus (Mouse) OX=10090 GN=Myo6 PE=1 SV=1	Q64331	Myo6	146 kDa	0.0023	KO Male NT high, WT Male NT low	13	12	12	5	2	5
49	Rho GDP-dissociation inhibitor 1 OS=Mus musculus (Mouse) OX=10090 GN=Arhgdia PE=1 SV=3	Q99PT1	Arhgdia	23 kDa	0.046	KO Male NT high, WT Male NT low	15	18	23	10	5	15
50	WD repeat-containing protein 1 OS=Mus musculus (Mouse) OX=10090 GN=Wdr1 PE=1 SV=3	O88342	Wdr1	66 kDa	0.0034	KO Male NT high, WT Male NT low	17	18	15	5	4	10
51	Ras GTPase-activating-like protein IQGAP1 OS=Mus musculus (Mouse) OX=10090 GN=Iqgap1 PE=1 SV=2	Q9JKF1	Iqgap1	189 kDa	0.015	KO Male NT high, WT Male NT low	16	11	20	1	1	6
52	6-phosphogluconate dehydrogenase, decarboxylating OS=Mus musculus (Mouse) OX=10090 GN=Pgd PE=1 SV=3	Q9DCD0	Pgd	53 kDa	0.05	KO Male NT high, WT Male NT low	13	19	20	12	7	13
53	Annexin A6 OS=Mus musculus (Mouse) OX=10090 GN=Anxa6 PE=1 SV=3	P14824	Anxa6	76 kDa	0.0029	KO Male NT high, WT Male NT low	15	12	17	3	0	2

54	Bifunctional 3'-phosphoadenosine 5'-phosphosulfate synthase 2 OS=Mus musculus (Mouse) OX=10090 GN=Papss2 PE=1 SV=2	O88428	Papss2	70 kDa	0.033	KO Male NT high, WT Male NT low	12	12	9	3	1	8
55	Cluster of Dihydropyrimidinase-related protein 2 OS=Mus musculus (Mouse) OX=10090 GN=Dpysl2 PE=1 SV=2 (O08553)	O08553 [2]	Dpysl2	62 kDa	0.01	KO Male NT high, WT Male NT low	11	13	15	4	2	8
56	Destrin OS=Mus musculus (Mouse) OX=10090 GN=Dstn PE=1 SV=3	Q9R0P5	Dstn	19 kDa	0.0019	KO Male NT high, WT Male NT low	12	13	16	9	12	11
57	Cathepsin Z OS=Mus musculus (Mouse) OX=10090 GN=Ctsz PE=1 SV=1	Q9WUU7	Ctsz	34 kDa	< 0.00010	KO Male NT high, WT Male NT low	20	19	21	3	4	3
58	Acyl-coenzyme A thioesterase 13 OS=Mus musculus (Mouse) OX=10090 GN=Acot13 PE=1 SV=1	Q9CQR4	Acot13	15 kDa	0.017	KO Male NT high, WT Male NT low	6	8	6	4	4	7
59	Annexin A2 OS=Mus musculus (Mouse) OX=10090 GN=Anxa2 PE=1 SV=2	P07356	Anxa2	39 kDa	0.029	KO Male NT high, WT Male NT low	18	11	13	9	6	8
60	Plastin-1 OS=Mus musculus (Mouse) OX=10090 GN=Pls1 PE=1 SV=1	Q3V0K9	Pls1	70 kDa	0.0018	KO Male NT high, WT Male NT low	7	6	8	3	3	2
61	Fatty acid-binding protein 5 OS=Mus musculus (Mouse) OX=10090 GN=Fabp5 PE=1 SV=3	Q05816	Fabp5	15 kDa	0.018	KO Male NT high, WT Male NT low	22	16	21	4	12	1
62	Branched-chain-amino-acid aminotransferase, mitochondrial OS=Mus musculus (Mouse) OX=10090 GN=Bcat2 PE=1 SV=2	O35855	Bcat2	44 kDa	0.0061	KO Male NT high, WT Male NT low	11	12	10	8	7	7
63	Glutathione S-transferase A3 OS=Mus musculus (Mouse) OX=10090 GN=Gsta3 PE=1 SV=2	P30115	Gsta3	25 kDa	0.0044	KO Male NT high, WT Male NT low	3	4	4	4	2	3
64	Polyadenylate-binding protein 1 OS=Mus musculus (Mouse) OX=10090 GN=Pabpc1 PE=1 SV=2	P29341	Pabpc1	71 kDa	0.02	KO Male NT high, WT Male NT low	15	13	13	10	2	8

65	Cluster of AP-1 complex subunit beta-1 OS=Mus musculus (Mouse) OX=10090 GN=Ap1b1 PE=1 SV=2 (O35643)	O35643 [2]	Ap1b1	104 kDa	0.0083	KO Male NT high, WT Male NT low	13	14	16	1	0	2
66	Leukotriene A-4 hydrolase OS=Mus musculus (Mouse) OX=10090 GN=Lta4h PE=1 SV=4	P24527	Lta4h	69 kDa	0.0045	KO Male NT high, WT Male NT low	13	16	12	3	0	5
67	Macrophage migration inhibitory factor OS=Mus musculus (Mouse) OX=10090 GN=Mif PE=1 SV=2	P34884	Mif	13 kDa	0.029	KO Male NT high, WT Male NT low	9	11	10	8	9	7
68	Serine/threonine-protein phosphatase 2A 65 kDa regulatory subunit A alpha isoform OS=Mus musculus (Mouse) OX=10090 GN=Ppp2r1a PE=1 SV=3	Q76MZ3	Ppp2r1a	65 kDa	0.02	KO Male NT high, WT Male NT low	9	13	8	4	2	4
69	Cluster of Actin-related protein 3 OS=Mus musculus (Mouse) OX=10090 GN=Actr3 PE=1 SV=3 (Q99JY9)	Q99JY9 [2]	Actr3	47 kDa	0.034	KO Male NT high, WT Male NT low	12	11	12	7	3	10
70	Programmed cell death 6-interacting protein OS=Mus musculus (Mouse) OX=10090 GN=Pdcd6ip PE=1 SV=3	Q9WU78	Pdcd6ip	96 kDa	0.0022	KO Male NT high, WT Male NT low	11	13	10	2	2	5
71	F-actin-capping protein subunit beta OS=Mus musculus (Mouse) OX=10090 GN=Capzb PE=1 SV=3	P47757	Capzb	31 kDa	0.0075	KO Male NT high, WT Male NT low	9	11	11	6	4	7
72	Talin-2 OS=Mus musculus (Mouse) OX=10090 GN=Tln2 PE=1 SV=3	Q71LX4	Tln2	254 kDa	0.024	KO Male NT high, WT Male NT low	4	3	3	1	0	0
73	Rab GDP dissociation inhibitor alpha OS=Mus musculus (Mouse) OX=10090 GN=Gdi1 PE=1 SV=3	P50396	Gdi1	51 kDa	0.028	KO Male NT high, WT Male NT low	7	8	7	1	0	3
74	Glutathione S-transferase theta-1 OS=Mus musculus (Mouse) OX=10090 GN=Gstt1 PE=1 SV=4	Q64471	Gstt1	27 kDa	0.034	KO Male NT high, WT Male NT low	8	12	10	4	4	7
75	Proteasome activator complex subunit 1 OS=Mus musculus	P97371	Psme1	29 kDa	0.043	KO Male NT high, WT Male NT low	9	9	12	7	6	7

	(Mouse) OX=10090 GN=Psme1 PE=1 SV=2											
76	Tripeptidyl-peptidase 1 OS=Mus musculus (Mouse) OX=10090 GN=Tpp1 PE=1 SV=2	O89023	Tpp1	61 kDa	0.0006	KO Male NT high, WT Male NT low	17	15	15	1	1	6
77	Lipopolysaccharide-responsive and beige-like anchor protein OS=Mus musculus (Mouse) OX=10090 GN=Lrba PE=1 SV=1	Q9ESE1	Lrba	317 kDa	0.007	KO Male NT high, WT Male NT low	6	7	9	2	0	2
78	Synaptic vesicle membrane protein VAT-1 homolog OS=Mus musculus (Mouse) OX=10090 GN=Vat1 PE=1 SV=3	Q62465	Vat1	43 kDa	0.00088	KO Male NT high, WT Male NT low	20	17	27	0	0	1
79	Annexin A4 OS=Mus musculus (Mouse) OX=10090 GN=Anxa4 PE=1 SV=4	P97429	Anxa4	36 kDa	0.019	KO Male NT high, WT Male NT low	12	9	13	6	4	2
80	Elongation factor 1-gamma OS=Mus musculus (Mouse) OX=10090 GN=Eef1g PE=1 SV=3	Q9D8N0	Eef1g	50 kDa	0.045	KO Male NT high, WT Male NT low	8	11	10	6	4	7
81	Ubiquitin carboxyl-terminal hydrolase 5 OS=Mus musculus (Mouse) OX=10090 GN=Usp5 PE=1 SV=1	P56399	Usp5	96 kDa	0.029	KO Male NT high, WT Male NT low	8	11	8	4	1	6
82	N-acetylglucosamine-6-sulfatase OS=Mus musculus (Mouse) OX=10090 GN=Gns PE=1 SV=1	Q8BFR4	Gns	61 kDa	0.00048	KO Male NT high, WT Male NT low	13	16	18	0	0	0
83	Voltage-dependent anion-selective channel protein 2 OS=Mus musculus (Mouse) OX=10090 GN=Vdac2 PE=1 SV=2	Q60930	Vdac2	32 kDa	0.044	KO Male NT high, WT Male NT low	10	7	8	5	2	4
84	Glyoxalase domain-containing protein 4 OS=Mus musculus (Mouse) OX=10090 GN=Glod4 PE=1 SV=1	Q9CPV4	Glod4	33 kDa	0.039	KO Male NT high, WT Male NT low	9	11	11	7	4	9
85	Legumain OS=Mus musculus (Mouse) OX=10090 GN=Lgmn PE=1 SV=1	O89017	Lgmn	49 kDa	0.0028	KO Male NT high, WT Male NT low	15	12	17	4	3	5

86	Sulfotransferase 1C2 OS=Mus musculus (Mouse) OX=10090 GN=Sult1c2 PE=1 SV=1	Q9D939	Sult1c2	35 kDa	0.017	KO Male NT high, WT Male NT low	9	9	8	4	0	5
87	Myosin light chain kinase, smooth muscle OS=Mus musculus (Mouse) OX=10090 GN=Mylk PE=1 SV=3	Q6PDN3	Mylk	213 kDa	0.012	KO Male NT high, WT Male NT low	8	6	7	4	2	2
88	Arylsulfatase B OS=Mus musculus (Mouse) OX=10090 GN=Arsb PE=1 SV=3	P50429	Arsb	60 kDa	0.00066	KO Male NT high, WT Male NT low	17	14	18	1	1	1
89	UDP-N-acetylhexosamine pyrophosphorylase-like protein 1 OS=Mus musculus (Mouse) OX=10090 GN=Uap1l1 PE=1 SV=1	Q3TW96	Uap1l1	57 kDa	< 0.00010	KO Male NT high, WT Male NT low	11	10	15	0	0	1
90	Cluster of Guanine nucleotide-binding protein G(i) subunit alpha-2 OS=Mus musculus (Mouse) OX=10090 GN=Gnai2 PE=1 SV=5 (P08752)	P08752 [4]	Gnai2	40 kDa	0.027	KO Male NT high, WT Male NT low	16	7	9	3	0	3
91	Actin-related protein 2/3 complex subunit 1B OS=Mus musculus (Mouse) OX=10090 GN=Arpc1b PE=1 SV=4	Q9WV32	Arpc1b	41 kDa	0.013	KO Male NT high, WT Male NT low	8	8	7	1	0	5
92	T-complex protein 1 subunit theta OS=Mus musculus (Mouse) OX=10090 GN=Cct8 PE=1 SV=3	P42932	Cct8	60 kDa	0.046	KO Male NT high, WT Male NT low	9	11	8	3	0	6
93	V-type proton ATPase subunit D OS=Mus musculus (Mouse) OX=10090 GN=Atp6v1d PE=1 SV=1	P57746	Atp6v1d	28 kDa	0.027	KO Male NT high, WT Male NT low	7	9	8	5	3	5
94	Cluster of Putative adenosylhomocysteinase 3 OS=Mus musculus (Mouse) OX=10090 GN=Ahcyl2 PE=1 SV=1 (Q68FL4)	Q68FL4 [2]	Ahcyl2	67 kDa	0.045	KO Male NT high, WT Male NT low	10	4	3	3	2	6
95	Retinoid-inducible serine carboxypeptidase OS=Mus musculus (Mouse) OX=10090 GN=Scpep1 PE=1 SV=2	Q920A5	Scpep1	51 kDa	0.00071	KO Male NT high, WT Male NT low	11	14	14	1	1	0

96	Lysosome-associated membrane glycoprotein 1 OS=Mus musculus (Mouse) OX=10090 GN=Lamp1 PE=1 SV=2	P11438	Lamp1	44 kDa	0.0017	KO Male NT high, WT Male NT low	10	7	11	0	1	0
97	Isoamyl acetate-hydrolyzing esterase 1 homolog OS=Mus musculus (Mouse) OX=10090 GN=Iah1 PE=1 SV=1	Q9DB29	Iah1	28 kDa	0.027	KO Male NT high, WT Male NT low	12	11	7	3	1	6
98	Procathepsin L OS=Mus musculus (Mouse) OX=10090 GN=Ctsl PE=1 SV=2	P06797	Ctsl	38 kDa	0.012	KO Male NT high, WT Male NT low	7	5	11	3	4	2
99	Beta-hexosaminidase subunit beta OS=Mus musculus (Mouse) OX=10090 GN=Hexb PE=1 SV=2	P20060	Hexb	61 kDa	0.00056	KO Male NT high, WT Male NT low	14	12	12	0	0	0
100	Galactose mutarotase OS=Mus musculus (Mouse) OX=10090 GN=Galm PE=1 SV=1	Q8K157	Galm	38 kDa	0.013	KO Male NT high, WT Male NT low	10	7	6	3	2	4
101	Adseverin OS=Mus musculus (Mouse) OX=10090 GN=Scin PE=1 SV=3	Q60604	Scin	80 kDa	0.022	KO Male NT high, WT Male NT low	9	6	10	4	1	1
102	26S proteasome non-ATPase regulatory subunit 2 OS=Mus musculus (Mouse) OX=10090 GN=Psmd2 PE=1 SV=1	Q8VDM4	Psmd2	100 kDa	0.02	KO Male NT high, WT Male NT low	5	7	9	4	1	3
103	NAD(P)H dehydrogenase [quinone] 1 OS=Mus musculus (Mouse) OX=10090 GN=Nqo1 PE=1 SV=3	Q64669	Nqo1	31 kDa	0.03	KO Male NT high, WT Male NT low	6	6	6	2	0	2
104	26S proteasome non-ATPase regulatory subunit 1 OS=Mus musculus (Mouse) OX=10090 GN=Psmd1 PE=1 SV=1	Q3TXS7	Psmd1	106 kDa	0.023	KO Male NT high, WT Male NT low	7	6	3	1	0	3
105	Coactosin-like protein OS=Mus musculus (Mouse) OX=10090 GN=Cotl1 PE=1 SV=3	Q9CQI6	Cotl1	16 kDa	0.0043	KO Male NT high, WT Male NT low	7	10	12	3	4	4
106	Glucosamine-6-phosphate isomerase 1 OS=Mus musculus (Mouse) OX=10090 GN=Gnpda1 PE=1 SV=3	O88958	Gnpda1	33 kDa	0.011	KO Male NT high, WT Male NT low	7	9	7	4	0	2

107	F-actin-capping protein subunit alpha-2 OS=Mus musculus (Mouse) OX=10090 GN=Capza2 PE=1 SV=3	P47754	Capza2	33 kDa	0.024	KO Male NT high, WT Male NT low	10	4	10	2	1	3
108	Galectin-3 OS=Mus musculus (Mouse) OX=10090 GN=Lgals3 PE=1 SV=3	P16110	Lgals3	28 kDa	0.0001	KO Male NT high, WT Male NT low	9	12	15	0	0	0
109	Coatomer subunit alpha OS=Mus musculus (Mouse) OX=10090 GN=Copa PE=1 SV=2	Q8CIE6	Copa	138 kDa	0.0065	KO Male NT high, WT Male NT low	7	8	5	2	0	4
110	Napsin-A OS=Mus musculus (Mouse) OX=10090 GN=Napsa PE=1 SV=1	O09043	Napsa	46 kDa	0.0002	KO Male NT high, WT Male NT low	5	4	4	1	0	1
111	Septin-7 OS=Mus musculus (Mouse) OX=10090 GN=Septin7 PE=1 SV=1	O55131	Septin7	51 kDa	0.009	KO Male NT high, WT Male NT low	4	5	6	0	0	3
112	Calpain-2 catalytic subunit OS=Mus musculus (Mouse) OX=10090 GN=Capn2 PE=1 SV=4	O08529	Capn2	80 kDa	0.011	KO Male NT high, WT Male NT low	10	6	9	2	0	3
113	Septin-2 OS=Mus musculus (Mouse) OX=10090 GN=Septin2 PE=1 SV=2	P42208	Septin2	42 kDa	0.0018	KO Male NT high, WT Male NT low	8	7	10	3	2	3
114	Lysosomal protective protein OS=Mus musculus (Mouse) OX=10090 GN=Ctsa PE=1 SV=1	P16675	Ctsa	54 kDa	< 0.00010	KO Male NT high, WT Male NT low	10	8	10	2	3	3
115	Actin-related protein 2 OS=Mus musculus (Mouse) OX=10090 GN=Actr2 PE=1 SV=1	P61161	Actr2	45 kDa	0.029	KO Male NT high, WT Male NT low	9	9	7	5	2	2
116	Actin-related protein 2/3 complex subunit 2 OS=Mus musculus (Mouse) OX=10090 GN=Arpc2 PE=1 SV=3	Q9CVB6	Arpc2	34 kDa	0.033	KO Male NT high, WT Male NT low	7	5	8	3	3	4
117	Acid ceramidase OS=Mus musculus (Mouse) OX=10090 GN=Asah1 PE=1 SV=1	Q9WV54	Asah1	45 kDa	0.0051	KO Male NT high, WT Male NT low	7	7	11	0	0	0
118	Septin-11 OS=Mus musculus (Mouse) OX=10090 GN=Septin11 PE=1 SV=4	Q8C1B7	Septin11	50 kDa	0.033	KO Male NT high, WT Male NT low	6	8	5	3	0	5

119	Coatomer subunit beta OS=Mus musculus (Mouse) OX=10090 GN=Copb1 PE=1 SV=1	Q9JIF7	Copb1	107 kDa	0.023	KO Male NT high, WT Male NT low	5	5	4	2	0	4
120	N-acetylneuraminate lyase OS=Mus musculus (Mouse) OX=10090 GN=Npl PE=1 SV=1	Q9DCJ9	Npl	35 kDa	0.022	KO Male NT high, WT Male NT low	6	9	8	4	2	4
121	Apolipoprotein E OS=Mus musculus (Mouse) OX=10090 GN=Apoe PE=1 SV=2	P08226	Apoe	36 kDa	0.0023	KO Male NT high, WT Male NT low	6	8	7	2	0	1
122	Macrophage-capping protein OS=Mus musculus (Mouse) OX=10090 GN=Capg PE=1 SV=2	P24452	Capg	39 kDa	0.0037	KO Male NT high, WT Male NT low	7	5	8	0	0	0
123	Dynactin subunit 1 OS=Mus musculus (Mouse) OX=10090 GN=Dctn1 PE=1 SV=3	O08788	Dctn1	142 kDa	0.0029	KO Male NT high, WT Male NT low	5	6	6	3	1	2
124	Lysosomal alpha-mannosidase OS=Mus musculus (Mouse) OX=10090 GN=Man2b1 PE=1 SV=4	O09159	Man2b1	115 kDa	0.05	KO Male NT high, WT Male NT low	7	8	19	0	0	0
125	Beta-galactosidase OS=Mus musculus (Mouse) OX=10090 GN=Glb1 PE=1 SV=1	P23780	Glb1	73 kDa	0.035	KO Male NT high, WT Male NT low	12	8	7	1	0	0
126	Rho GDP-dissociation inhibitor 2 OS=Mus musculus (Mouse) OX=10090 GN=Arhgdib PE=1 SV=3	Q61599	Arhgdib	23 kDa	0.0038	KO Male NT high, WT Male NT low	7	6	7	2	3	1
127	V-type proton ATPase subunit C 1 OS=Mus musculus (Mouse) OX=10090 GN=Atp6v1c1 PE=1 SV=4	Q9Z1G3	Atp6v1c1	44 kDa	0.024	KO Male NT high, WT Male NT low	5	4	3	1	1	2
128	Coronin-1C OS=Mus musculus (Mouse) OX=10090 GN=Coro1c PE=1 SV=2	Q9WUM4	Coro1c	53 kDa	0.02	KO Male NT high, WT Male NT low	7	6	9	4	2	1
129	ATP-dependent 6-phosphofructokinase, platelet type OS=Mus musculus (Mouse) OX=10090 GN=Pfkp PE=1 SV=1	Q9WUA3	Pfkp	85 kDa	0.011	KO Male NT high, WT Male NT low	2	3	3	0	0	0

130	40S ribosomal protein S13 OS=Mus musculus (Mouse) OX=10090 GN=Rps13 PE=1 SV=2	P62301	Rps13	17 kDa	0.0075	KO Male NT high, WT Male NT low	6	4	5	4	3	3
131	T-complex protein 1 subunit alpha OS=Mus musculus (Mouse) OX=10090 GN=Tcp1 PE=1 SV=3	P11983	Tcp1	60 kDa	0.0022	KO Male NT high, WT Male NT low	3	6	6	1	0	2
132	Glutathione S-transferase omega-1 OS=Mus musculus (Mouse) OX=10090 GN=Gsto1 PE=1 SV=2	O09131	Gsto1	27 kDa	0.011	KO Male NT high, WT Male NT low	8	4	5	1	0	1
133	Acid sphingomyelinase-like phosphodiesterase 3a OS=Mus musculus (Mouse) OX=10090 GN=Smpdl3a PE=1 SV=2	P70158	Smpdl3a	50 kDa	0.0064	KO Male NT high, WT Male NT low	8	10	12	1	0	0
134	Coronin-1B OS=Mus musculus (Mouse) OX=10090 GN=Coro1b PE=1 SV=1	Q9WUM3	Coro1b	54 kDa	0.031	KO Male NT high, WT Male NT low	4	4	7	1	1	1
135	Glycogen phosphorylase, brain form OS=Mus musculus (Mouse) OX=10090 GN=Pygb PE=1 SV=3	Q8CI94	Pygb	97 kDa	0.002	KO Male NT high, WT Male NT low	4	7	4	0	0	0
136	Bifunctional purine biosynthesis protein ATIC OS=Mus musculus (Mouse) OX=10090 GN=Atic PE=1 SV=2	Q9CWJ9	Atic	64 kDa	0.044	KO Male NT high, WT Male NT low	4	7	5	3	0	4
137	Insulin-degrading enzyme OS=Mus musculus (Mouse) OX=10090 GN=Ide PE=1 SV=1	Q9JHR7	Ide	118 kDa	0.044	KO Male NT high, WT Male NT low	5	7	3	0	0	4
138	26S proteasome regulatory subunit 6B OS=Mus musculus (Mouse) OX=10090 GN=Psmc4 PE=1 SV=2	P54775	Psmc4	47 kDa	0.026	KO Male NT high, WT Male NT low	5	3	4	2	2	2
139	Lysosomal alpha-glucosidase OS=Mus musculus (Mouse) OX=10090 GN=Gaa PE=1 SV=2	P70699	Gaa	106 kDa	0.0065	KO Male NT high, WT Male NT low	4	5	7	0	0	0
140	6-phosphogluconolactonase OS=Mus musculus (Mouse) OX=10090 GN=Pgls PE=1 SV=1	Q9CQ60	Pgls	27 kDa	0.043	KO Male NT high, WT Male NT low	5	3	6	1	0	3
141	Alpha-N-acetylgalactosaminidase OS=Mus musculus (Mouse) OX=10090 GN=Naga PE=1 SV=2	Q9QWR8	Naga	47 kDa	0.0032	KO Male NT high, WT Male NT low	10	7	11	1	0	0

142	Gamma-interferon-inducible lysosomal thiol reductase OS=Mus musculus (Mouse) OX=10090 GN=Ifi30 PE=1 SV=3	Q9ESY9	Ifi30	28 kDa	< 0.00010	KO Male NT high, WT Male NT low	10	9	12	0	0	0
143	Creatine kinase U-type, mitochondrial OS=Mus musculus (Mouse) OX=10090 GN=Ckmt1 PE=1 SV=1	P30275	Ckmt1	47 kDa	0.017	KO Male NT high, WT Male NT low	7	4	7	2	1	3
144	Cluster of ADP-ribosylation factor-like protein 8B OS=Mus musculus (Mouse) OX=10090 GN=Arl8b PE=1 SV=1 (Q9CQW2)	Q9CQW2 [2]	Arl8b	22 kDa	0.0009	KO Male NT high, WT Male NT low	4	3	8	0	0	1
145	Dipeptidyl peptidase 2 OS=Mus musculus (Mouse) OX=10090 GN=Dpp7 PE=1 SV=2	Q9ET22	Dpp7	56 kDa	0.0045	KO Male NT high, WT Male NT low	9	11	14	0	0	0
146	Lysophospholipase-like protein 1 OS=Mus musculus (Mouse) OX=10090 GN=Lyplal1 PE=1 SV=3	Q3UFF7	Lyplal1	26 kDa	0.003	KO Male NT high, WT Male NT low	6	4	4	0	0	0
147	ATP-dependent RNA helicase DDX1 OS=Mus musculus (Mouse) OX=10090 GN=Ddx1 PE=1 SV=1	Q91VR5	Ddx1	83 kDa	0.025	KO Male NT high, WT Male NT low	5	4	3	1	0	3
148	Alkaline phosphatase, tissue-nonspecific isozyme OS=Mus musculus (Mouse) OX=10090 GN=Alpl PE=1 SV=2	P09242	Alpl	58 kDa	0.00078	KO Male NT high, WT Male NT low	5	5	4	0	0	1
149	T-complex protein 1 subunit eta OS=Mus musculus (Mouse) OX=10090 GN=Cct7 PE=1 SV=1	P80313	Cct7	60 kDa	0.033	KO Male NT high, WT Male NT low	5	5	5	3	1	4
150	Eukaryotic translation initiation factor 3 subunit C OS=Mus musculus (Mouse) OX=10090 GN=Eif3c PE=1 SV=1	Q8R1B4	Eif3c	106 kDa	0.025	KO Male NT high, WT Male NT low	3	4	3	1	0	2
151	T-complex protein 1 subunit delta OS=Mus musculus (Mouse) OX=10090 GN=Cct4 PE=1 SV=3	P80315	Cct4	58 kDa	0.034	KO Male NT high, WT Male NT low	4	7	4	2	1	2

152	Vacuolar protein sorting-associated protein 29 OS=Mus musculus (Mouse) OX=10090 GN=Vps29 PE=1 SV=1	Q9QZ88	Vps29	20 kDa	0.0026	KO Male NT high, WT Male NT low	5	4	5	1	1	2
153	4F2 cell-surface antigen heavy chain OS=Mus musculus (Mouse) OX=10090 GN=Slc3a2 PE=1 SV=1	P10852	Slc3a2	58 kDa	0.0065	KO Male NT high, WT Male NT low	4	4	4	1	0	2
154	Annexin A11 OS=Mus musculus (Mouse) OX=10090 GN=Anxa11 PE=1 SV=2	P97384	Anxa11	54 kDa	0.0053	KO Male NT high, WT Male NT low	5	4	5	2	0	1
155	V-type proton ATPase 116 kDa subunit a isoform 4 OS=Mus musculus (Mouse) OX=10090 GN=Atp6v0a4 PE=1 SV=1	Q920R6	Atp6v0a4	96 kDa	0.008	KO Male NT high, WT Male NT low	8	5	5	1	0	0
156	Lysosome-associated membrane glycoprotein 2 OS=Mus musculus (Mouse) OX=10090 GN=Lamp2 PE=1 SV=2	P17047	Lamp2	46 kDa	0.0023	KO Male NT high, WT Male NT low	3	3	4	0	0	0
157	SH3 domain-binding glutamic acid-rich-like protein OS=Mus musculus (Mouse) OX=10090 GN=Sh3bgrl PE=1 SV=1	Q9JJU8	Sh3bgrl	13 kDa	0.034	KO Male NT high, WT Male NT low	5	4	7	3	3	2
158	60S ribosomal protein L23 OS=Mus musculus (Mouse) OX=10090 GN=Rpl23 PE=1 SV=1	P62830	Rpl23	15 kDa	0.033	KO Male NT high, WT Male NT low	5	4	4	2	0	3
159	V-type proton ATPase subunit d 1 OS=Mus musculus (Mouse) OX=10090 GN=Atp6v0d1 PE=1 SV=2	P51863	Atp6v0d1	40 kDa	0.0072	KO Male NT high, WT Male NT low	6	7	6	3	0	3
160	Dipeptidyl peptidase 1 OS=Mus musculus (Mouse) OX=10090 GN=Ctsc PE=1 SV=1	P97821	Ctsc	52 kDa	0.019	KO Male NT high, WT Male NT low	9	4	13	0	0	0
161	Cluster of Chitinase-like protein 3 OS=Mus musculus (Mouse) OX=10090 GN=Chil3 PE=1 SV=2 (O35744)	O35744 [2]	Chil3	44 kDa	0.018	KO Male NT high, WT Male NT low	6	4	4	2	2	1

162	Glutaredoxin-3 OS=Mus musculus (Mouse) OX=10090 GN=Glrx3 PE=1 SV=1	Q9CQM9	Glrx3	38 kDa	0.0013	KO Male NT high, WT Male NT low	6	4	6	3	3	3
163	26S proteasome regulatory subunit 4 OS=Mus musculus (Mouse) OX=10090 GN=Psmc1 PE=1 SV=1	P62192	Psmc1	49 kDa	0.034	KO Male NT high, WT Male NT low	3	4	5	1	1	4
164	Proteasome subunit beta type-2 OS=Mus musculus (Mouse) OX=10090 GN=Psmb2 PE=1 SV=1	Q9R1P3	Psmb2	23 kDa	0.025	KO Male NT high, WT Male NT low	2	3	3	0	1	2
165	Proliferation-associated protein 2G4 OS=Mus musculus (Mouse) OX=10090 GN=Pa2g4 PE=1 SV=3	P50580	Pa2g4	44 kDa	0.047	KO Male NT high, WT Male NT low	2	3	4	0	0	2
166	Di-N-acetylchitobiase OS=Mus musculus (Mouse) OX=10090 GN=Ctbs PE=1 SV=2	Q8R242	Ctbs	41 kDa	0.011	KO Male NT high, WT Male NT low	6	5	9	0	0	0
167	TAR DNA-binding protein 43 OS=Mus musculus (Mouse) OX=10090 GN=Tardbp PE=1 SV=1	Q921F2	Tardbp	45 kDa	0.033	KO Male NT high, WT Male NT low	5	4	3	2	0	2
168	26S proteasome non-ATPase regulatory subunit 7 OS=Mus musculus (Mouse) OX=10090 GN=Psmd7 PE=1 SV=2	P26516	Psmd7	37 kDa	0.017	KO Male NT high, WT Male NT low	4	5	3	1	0	2
169	Fructose-1,6-bisphosphatase isozyme 2 OS=Mus musculus (Mouse) OX=10090 GN=Fbp2 PE=1 SV=2	P70695	Fbp2	37 kDa	0.039	KO Male NT high, WT Male NT low	1	3	2	0	0	0
170	Plasma alpha-L-fucosidase OS=Mus musculus (Mouse) OX=10090 GN=Fuca2 PE=1 SV=1	Q99KR8	Fuca2	54 kDa	0.019	KO Male NT high, WT Male NT low	4	6	7	0	0	0
171	ATP-dependent 6-phosphofructokinase, liver type OS=Mus musculus (Mouse) OX=10090 GN=Pfkl PE=1 SV=4	P12382	Pfkl	85 kDa	0.038	KO Male NT high, WT Male NT low	3	2	1	0	0	1
172	F-actin-capping protein subunit alpha-1 OS=Mus musculus (Mouse) OX=10090 GN=Capza1 PE=1 SV=4	P47753	Capza1	33 kDa	0.0053	KO Male NT high, WT Male NT low	3	3	3	1	1	0

173	Ras-related protein Rab-2A OS=Mus musculus (Mouse) OX=10090 GN=Rab2a PE=1 SV=1	P53994	Rab2a	24 kDa	0.024	KO Male NT high, WT Male NT low	6	5	4	3	2	2
174	Beta-hexosaminidase subunit alpha OS=Mus musculus (Mouse) OX=10090 GN=Hexa PE=1 SV=2	P29416	Hexa	61 kDa	0.0002	KO Male NT high, WT Male NT low	5	6	7	0	0	0
175	60S ribosomal protein L9 OS=Mus musculus (Mouse) OX=10090 GN=Rpl9 PE=2 SV=2	P51410	Rpl9	22 kDa	0.021	KO Male NT high, WT Male NT low	5	4	4	2	1	3
176	Pyruvate kinase PKLR OS=Mus musculus (Mouse) OX=10090 GN=Pklr PE=1 SV=1	P53657	Pklr	62 kDa	0.0013	KO Male NT high, WT Male NT low	4	4	4	0	0	1
177	Epididymis-specific alpha-mannosidase OS=Mus musculus (Mouse) OX=10090 GN=Man2b2 PE=1 SV=2	O54782	Man2b2	116 kDa	0.003	KO Male NT high, WT Male NT low	6	3	5	0	0	0
178	26S proteasome non-ATPase regulatory subunit 6 OS=Mus musculus (Mouse) OX=10090 GN=Psmd6 PE=1 SV=1	Q99JI4	Psmd6	46 kDa	0.024	KO Male NT high, WT Male NT low	3	3	4	1	0	2
179	Serine/threonine-protein kinase PAK 2 OS=Mus musculus (Mouse) OX=10090 GN=Pak2 PE=1 SV=1	Q8CIN4	Pak2	58 kDa	0.013	KO Male NT high, WT Male NT low	2	3	3	1	1	1
180	N-acylglucosamine 2-epimerase OS=Mus musculus (Mouse) OX=10090 GN=Renbp PE=1 SV=3	P82343	Renbp	50 kDa	0.034	KO Male NT high, WT Male NT low	4	4	4	0	0	3
181	Cluster of Ribose-phosphate pyrophosphokinase 2 OS=Mus musculus (Mouse) OX=10090 GN=Prps2 PE=1 SV=4 (Q9CS42)	Q9CS42 [2]	Prps2	35 kDa	0.0078	KO Male NT high, WT Male NT low	3	3	2	0	0	1
182	Transmembrane glycoprotein NMB OS=Mus musculus (Mouse) OX=10090 GN=Gpnmb PE=1 SV=2	Q99P91	Gpnmb	64 kDa	0.0075	KO Male NT high, WT Male NT low	2	1	1	0	0	0
183	Beta-centractin OS=Mus musculus (Mouse) OX=10090 GN=Actr1b PE=1 SV=1	Q8R5C5	Actr1b	42 kDa	0.035	KO Male NT high, WT Male NT low	2	2	2	1	0	1

184	cAMP-dependent protein kinase type II-alpha regulatory subunit OS=Mus musculus (Mouse) OX=10090 GN=Prkar2a PE=1 SV=2	P12367	Prkar2a	45 kDa	0.016	KO Male NT high, WT Male NT low	1	2	4	0	0	1
185	Importin subunit beta-1 OS=Mus musculus (Mouse) OX=10090 GN=Kpnb1 PE=1 SV=2	P70168	Kpnb1	97 kDa	0.0011	KO Male NT high, WT Male NT low	4	4	4	0	0	1
186	Dynamin-2 OS=Mus musculus (Mouse) OX=10090 GN=Dnm2 PE=1 SV=2	P39054	Dnm2	98 kDa	0.035	KO Male NT high, WT Male NT low	3	7	5	0	0	2
187	Putative phospholipase B-like 2 OS=Mus musculus (Mouse) OX=10090 GN=Plbd2 PE=1 SV=2	Q3TCN2	Plbd2	66 kDa	0.00056	KO Male NT high, WT Male NT low	3	4	3	0	0	0
188	Proteasome activator complex subunit 2 OS=Mus musculus (Mouse) OX=10090 GN=Psme2 PE=1 SV=4	P97372	Psme2	27 kDa	0.0031	KO Male NT high, WT Male NT low	4	4	5	0	0	1
189	ATPase GET3 OS=Mus musculus (Mouse) OX=10090 GN=Get3 PE=1 SV=2	O54984	Get3	39 kDa	0.00098	KO Male NT high, WT Male NT low	4	5	4	0	0	0
190	Tissue alpha-L-fucosidase OS=Mus musculus (Mouse) OX=10090 GN=Fuca1 PE=1 SV=1	Q99LJ1	Fuca1	52 kDa	0.003	KO Male NT high, WT Male NT low	4	6	6	0	0	0
191	Heterogeneous nuclear ribonucleoprotein A0 OS=Mus musculus (Mouse) OX=10090 GN=Hnrnpa0 PE=1 SV=1	Q9CX86	Hnrnpa0	31 kDa	0.013	KO Male NT high, WT Male NT low	3	2	3	1	0	1
192	ATP-dependent (S)-NAD(P)H-hydrate dehydratase OS=Mus musculus (Mouse) OX=10090 GN=Naxd PE=1 SV=1	Q9CZ42	Naxd	37 kDa	0.035	KO Male NT high, WT Male NT low	3	4	3	2	0	2
193	Cluster of Histone-binding protein RBBP4 OS=Mus musculus (Mouse) OX=10090 GN=Rbbp4 PE=1 SV=5 (Q60972)	Q60972 [2]	Rbbp4	48 kDa	0.026	KO Male NT high, WT Male NT low	2	3	4	1	1	1

194	Cluster of Glucose-6-phosphate 1-dehydrogenase X OS=Mus musculus (Mouse) OX=10090 GN=G6pdx PE=1 SV=3 (Q00612)	Q00612 [2]	G6pdx	59 kDa	0.013	KO Male NT high, WT Male NT low	2	3	5	1	0	0
195	60S ribosomal protein L27a OS=Mus musculus (Mouse) OX=10090 GN=Rpl27a PE=1 SV=5	P14115	Rpl27a	17 kDa	0.016	KO Male NT high, WT Male NT low	3	3	2	2	2	2
196	Medium-chain acyl-CoA ligase ACSF2, mitochondrial OS=Mus musculus (Mouse) OX=10090 GN=Acsf2 PE=1 SV=1	Q8VCW8	Acsf2	68 kDa	0.013	KO Male NT high, WT Male NT low	6	3	4	0	0	1
197	Septin-9 OS=Mus musculus (Mouse) OX=10090 GN=Septin9 PE=1 SV=1	Q80UG5	Septin9	66 kDa	0.0065	KO Male NT high, WT Male NT low	1	1	2	0	0	0
198	Phosphoglucomutase-2 OS=Mus musculus (Mouse) OX=10090 GN=Pgm2 PE=1 SV=1	Q7TSV4	Pgm2	69 kDa	0.035	KO Male NT high, WT Male NT low	2	2	4	0	0	1
199	Arginine--tRNA ligase, cytoplasmic OS=Mus musculus (Mouse) OX=10090 GN=Rars1 PE=1 SV=2	Q9D0I9	Rars1	76 kDa	0.0011	KO Male NT high, WT Male NT low	4	4	5	1	0	1
200	Calponin-2 OS=Mus musculus (Mouse) OX=10090 GN=Cnn2 PE=1 SV=1	Q08093	Cnn2	33 kDa	0.013	KO Male NT high, WT Male NT low	3	2	2	1	0	0
201	Perilipin-3 OS=Mus musculus (Mouse) OX=10090 GN=Plin3 PE=1 SV=1	Q9DBG5	Plin3	47 kDa	0.016	KO Male NT high, WT Male NT low	1	1	2	0	1	0
202	Resistin OS=Mus musculus (Mouse) OX=10090 GN=Retn PE=1 SV=1	Q99P87	Retn	12 kDa	0.026	KO Male NT high, WT Male NT low	2	1	3	0	0	0
203	Dihydropyrimidinase-related protein 3 OS=Mus musculus (Mouse) OX=10090 GN=Dpysl3 PE=1 SV=1	Q62188	Dpysl3	62 kDa	0.0053	KO Male NT high, WT Male NT low	1	1	3	0	0	0
204	Corticosteroid 11-beta-dehydrogenase isozyme 2 OS=Mus musculus (Mouse) OX=10090 GN=Hsd11b2 PE=1 SV=2	P51661	Hsd11b2	42 kDa	0.011	KO Male NT high, WT Male NT low	3	2	2	0	0	1

205	Aminopeptidase B OS=Mus musculus (Mouse) OX=10090 GN=Rnpep PE=1 SV=2	Q8VCT3	Rnpep	72 kDa	0.019	KO Male NT high, WT Male NT low	5	2	2	0	0	0
206	Fetuin-B OS=Mus musculus (Mouse) OX=10090 GN=Fetub PE=1 SV=1	Q9QXC1	Fetub	43 kDa	0.018	KO Male NT high, WT Male NT low	4	3	5	1	1	0
207	Platelet-activating factor acetylhydrolase IB subunit beta OS=Mus musculus (Mouse) OX=10090 GN=Pafah1b2 PE=1 SV=2	Q61206	Pafah1b2	26 kDa	0.025	KO Male NT high, WT Male NT low	1	3	3	0	0	0
208	Elongation factor Ts, mitochondrial OS=Mus musculus (Mouse) OX=10090 GN=Tsfm PE=1 SV=1	Q9CZR8	Tsfm	35 kDa	0.016	KO Male NT high, WT Male NT low	1	1	2	0	0	0
209	60S ribosomal protein L21 OS=Mus musculus (Mouse) OX=10090 GN=Rpl21 PE=1 SV=3	O09167	Rpl21	19 kDa	0.013	KO Male NT high, WT Male NT low	2	2	1	1	0	0
210	Valine--tRNA ligase OS=Mus musculus (Mouse) OX=10090 GN=Vars1 PE=1 SV=1	Q9Z1Q9	Vars1	140 kDa	0.033	KO Male NT high, WT Male NT low	2	2	4	1	0	1
211	Major vault protein OS=Mus musculus (Mouse) OX=10090 GN=Mvp PE=1 SV=4	Q9EQK5	Mvp	96 kDa	0.00015	KO Male NT high, WT Male NT low	4	5	4	0	0	0
212	S-methyl-5'-thioadenosine phosphorylase OS=Mus musculus (Mouse) OX=10090 GN=Mtap PE=1 SV=1	Q9CQ65	Mtap	31 kDa	0.016	KO Male NT high, WT Male NT low	3	2	2	0	0	0
213	Vacuolar protein sorting-associated protein 26A OS=Mus musculus (Mouse) OX=10090 GN=Vps26a PE=1 SV=1	P40336	Vps26a	38 kDa	0.013	KO Male NT high, WT Male NT low	2	3	2	0	0	1
214	Pre-mRNA-processing factor 19 OS=Mus musculus (Mouse) OX=10090 GN=Prpf19 PE=1 SV=1	Q99KP6	Prpf19	55 kDa	0.024	KO Male NT high, WT Male NT low	2	2	2	1	0	1
215	26S proteasome non-ATPase regulatory subunit 13 OS=Mus musculus (Mouse) OX=10090 GN=Psmd13 PE=1 SV=1	Q9WVJ2	Psmd13	43 kDa	0.029	KO Male NT high, WT Male NT low	4	5	3	0	0	2

216	Collagen alpha-2(VI) chain OS=Mus musculus (Mouse) OX=10090 GN=Col6a2 PE=1 SV=3	Q02788	Col6a2	110 kDa	0.026	KO Male NT high, WT Male NT low	3	1	2	0	0	0
217	Cluster of Mitogen-activated protein kinase 3 OS=Mus musculus (Mouse) OX=10090 GN=Mapk3 PE=1 SV=5 (Q63844)	Q63844 [2]	Mapk3	43 kDa	0.016	KO Male NT high, WT Male NT low	4	2	2	0	0	1
218	Ragulator complex protein LAMTOR3 OS=Mus musculus (Mouse) OX=10090 GN=Lamtor3 PE=1 SV=1	O88653	Lamtor3	14 kDa	0.00056	KO Male NT high, WT Male NT low	2	3	2	0	0	0
219	H-2 class II histocompatibility antigen gamma chain OS=Mus musculus (Mouse) OX=10090 GN=Cd74 PE=1 SV=3	P04441	Cd74	32 kDa	0.0013	KO Male NT high, WT Male NT low	1	1	1	0	0	0
220	26S proteasome non-ATPase regulatory subunit 12 OS=Mus musculus (Mouse) OX=10090 GN=Psmd12 PE=1 SV=4	Q9D8W5	Psmd12	53 kDa	0.019	KO Male NT high, WT Male NT low	2	5	3	0	0	0
221	Collagen alpha-1(XVIII) chain OS=Mus musculus (Mouse) OX=10090 GN=Col18a1 PE=1 SV=4	P39061	Col18a1	182 kDa	0.0075	KO Male NT high, WT Male NT low	2	2	1	0	0	0
222	Calcium/calmodulin-dependent protein kinase type II subunit delta OS=Mus musculus (Mouse) OX=10090 GN=Camk2d PE=1 SV=1	Q6PHZ2	Camk2d	56 kDa	0.0075	KO Male NT high, WT Male NT low	2	2	1	0	0	0
223	Pulmonary surfactant-associated protein B OS=Mus musculus (Mouse) OX=10090 GN=Sftpb PE=1 SV=1	P50405	Sftpb	42 kDa	0.0013	KO Male NT high, WT Male NT low	3	2	3	0	0	0
224	Dual specificity mitogen-activated protein kinase kinase 1 OS=Mus musculus (Mouse) OX=10090 GN=Map2k1 PE=1 SV=2	P31938	Map2k1	43 kDa	0.026	KO Male NT high, WT Male NT low	1	2	0	0	0	0

225	Platelet glycoprotein 4 OS=Mus musculus (Mouse) OX=10090 GN=Cd36 PE=1 SV=2	Q08857	Cd36	53 kDa	0.047	KO Male NT high, WT Male NT low	1	1	1	1	0	0
226	Lupus La protein homolog OS=Mus musculus (Mouse) OX=10090 GN=Ssb PE=1 SV=1	P32067	Ssb	48 kDa	0.047	KO Male NT high, WT Male NT low	2	1	1	0	0	1
227	Syntaxin-binding protein 2 OS=Mus musculus (Mouse) OX=10090 GN=Stxbp2 PE=1 SV=1	Q64324	Stxbp2	66 kDa	0.016	KO Male NT high, WT Male NT low	1	1	2	0	0	0
228	Methylthioribose-1-phosphate isomerase OS=Mus musculus (Mouse) OX=10090 GN=Mri1 PE=1 SV=1	Q9CQT1	Mri1	39 kDa	0.0078	KO Male NT high, WT Male NT low	3	3	1	0	0	1
229	Protein CREG1 OS=Mus musculus (Mouse) OX=10090 GN=Creg1 PE=1 SV=1	O88668	Creg1	24 kDa	0.0075	KO Male NT high, WT Male NT low	2	1	2	0	0	0
230	LIM and senescent cell antigen-like-containing domain protein 1 OS=Mus musculus (Mouse) OX=10090 GN=Lims1 PE=1 SV=3	Q99JW4	Lims1	37 kDa	0.0022	KO Male NT high, WT Male NT low	3	2	2	0	0	0
231	Ribonuclease T2-A OS=Mus musculus (Mouse) OX=10090 GN=Rnaset2a PE=1 SV=1	C0HKG5 (+1)	Rnaset2a	30 kDa	0.016	KO Male NT high, WT Male NT low	2	2	2	1	1	0
232	Beta-2-glycoprotein 1 OS=Mus musculus (Mouse) OX=10090 GN=Apoh PE=1 SV=1	Q01339	Apoh	39 kDa	0.0075	KO Male NT high, WT Male NT low	2	2	1	0	0	0
233	Glycerate kinase OS=Mus musculus (Mouse) OX=10090 GN=Glyctk PE=1 SV=1	Q8QZY2	Glyctk	55 kDa	0.047	KO Male NT high, WT Male NT low	2	2	1	0	0	1
234	STE20/SPS1-related proline-alanine-rich protein kinase OS=Mus musculus (Mouse) OX=10090 GN=Stk39 PE=1 SV=1	Q9Z1W9	Stk39	60 kDa	0.016	KO Male NT high, WT Male NT low	1	2	1	0	0	0
235	Protein transport protein Sec23A OS=Mus musculus (Mouse) OX=10090 GN=Sec23a PE=1 SV=2	Q01405	Sec23a	86 kDa	0.047	KO Male NT high, WT Male NT low	1	1	2	0	0	1

236	Cystatin-C OS=Mus musculus (Mouse) OX=10090 GN=Cst3 PE=1 SV=2	P21460	Cst3	16 kDa	0.016	KO Male NT high, WT Male NT low	2	2	3	0	0	0
237	Biglycan OS=Mus musculus (Mouse) OX=10090 GN=Bgn PE=1 SV=1	P28653	Bgn	42 kDa	0.026	KO Male NT high, WT Male NT low	2	1	1	0	0	0
238	Sialate O-acetylesterase OS=Mus musculus (Mouse) OX=10090 GN=Siae PE=1 SV=3	P70665	Siae	61 kDa	0.0075	KO Male NT high, WT Male NT low	1	2	2	0	0	0
239	Retinol-binding protein 4 OS=Mus musculus (Mouse) OX=10090 GN=Rbp4 PE=1 SV=2	Q00724	Rbp4	23 kDa	0.047	KO Male NT high, WT Male NT low	1	2	2	1	0	0
240	COP9 signalosome complex subunit 2 OS=Mus musculus (Mouse) OX=10090 GN=Cops2 PE=1 SV=1	P61202	Cops2	52 kDa	0.016	KO Male NT high, WT Male NT low	2	1	1	0	0	0
241	Ras-related protein Ral-B OS=Mus musculus (Mouse) OX=10090 GN=Ralb PE=1 SV=1	Q9JIW9	Ralb	23 kDa	0.016	KO Male NT high, WT Male NT low	1	2	1	0	0	0
242	N-acetylglucosamine-6-phosphate deacetylase OS=Mus musculus (Mouse) OX=10090 GN=Amdhd2 PE=1 SV=1	Q8JZV7	Amdhd2	44 kDa	0.013	KO Male NT high, WT Male NT low	2	2	3	0	0	1
243	ADP-ribosylation factor-like protein 6-interacting protein 1 OS=Mus musculus (Mouse) OX=10090 GN=Arl6ip1 PE=1 SV=1	Q9JKW0	Arl6ip1	23 kDa	0.016	KO Male NT high, WT Male NT low	1	2	1	0	0	0

m/z	Structure	*m/z*	Structure	*m/z*	Structure
**1240.60**	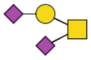	**2403.18**	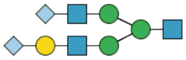 **and** 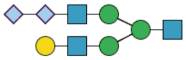	**3357.66**	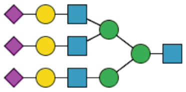
**1532.76**	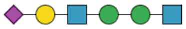				
**1562.77**	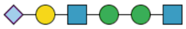	**2547.26**	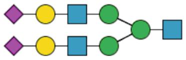	**3447.69**	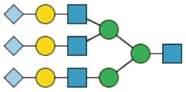
**1981.98**	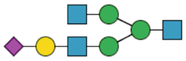	**2577.27**	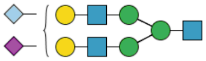	**3806.88**	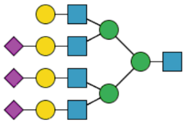
**2011.99**	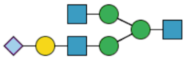	**2607.28**	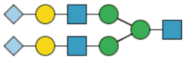	**3838.87**	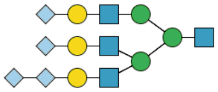 **and** 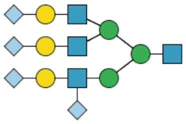
**2186.08**	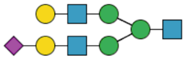	**2998.46**	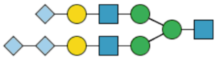 **and** 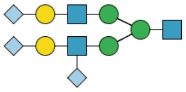		
**2216.09**	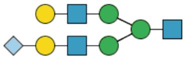				
**2343.16**	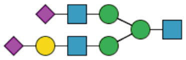 **and** 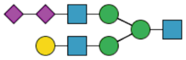	**3056.50**	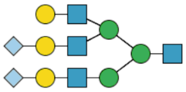		

Supplementary Table 3. Complete list of sialylated free oligosaccharides identified by MALDI TOF MS and MS/MS, in the water-soluble fraction of proteins extracted from kidney tissues of *Neu1*$^{\Delta Ex3}$ mice.

m/z	Structure	WT (n.3) Relative area mean± SD	*$Neu1^{\Delta Ex3}$* (n.4) Relative area mean± SD	*p-value*
1981.98	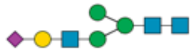	0.024 ± 0.008	0.232 ± 0.093	**<u>1.11E-06</u>**
2156.07	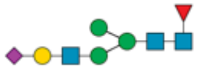	0.050 ± 0.015	0.478 ± 0.244	**<u>2.40E-05</u>**
2390.18	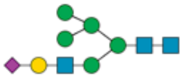	0.066 ± 0.022	0.212 ± 0.107	**<u>4.06E-04</u>**
2401.20	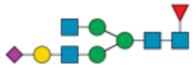	0.130 ± 0.038	0.379 ± 0.218	**<u>1.66E-03</u>**
2431.21	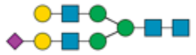	0.183 ± 0.042	0.424 ± 0.231	**<u>3.15E-03</u>**
2605.30	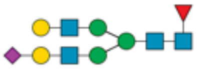	0.408 ± 0.076	0.982 ± 0.517	**<u>1.96E-03</u>**
2635.31	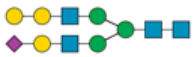	0.620 ± 0.118	0.671 ± 0.333	3.31E-01
2809.40	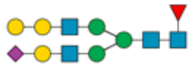	0.312 ± 0.098	0.438 ± 0.304	1.25E-01
2966.47	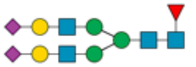	0.218 ± 0.040	0.728 ± 0.313	**<u>5.91E-05</u>**
3054.52	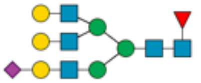	0.337 ± 0.027	0.376 ± 0.213	2.93E-01
3415.70	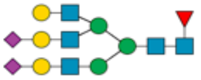	0.101 ± 0.023	0.222 ± 0.181	**<u>3.11E-02</u>**
3776.87	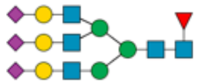	0.085 ± 0.024	0.175 ± 0.123	**<u>2.17E-02</u>**
Total sialylated structures		2.534 ± 0.269	5.318 ± 2.566	**<u>2.25E-03</u>**

Supplementary Table 4. Main N-linked sialylated glycans are abnormally abundant in *$Neu1^{\Delta Ex3}$* mouse kidney tissues.

N-glycan structures were identified by MALDI MS/MS, and the corresponding peak areas normalized for total areas of MS signals within the mass range of 1000-4000 Da to make measurements comparable across different samples. Statistically significant differences between WT and *$Neu1^{\Delta Ex3}$* glycans are shown in bold font and underlined.
